# Combining gene mutation with gene expression data improves outcome prediction in myelodysplastic syndromes

**DOI:** 10.1038/ncomms6901

**Published:** 2015-01-09

**Authors:** Moritz Gerstung, Andrea Pellagatti, Luca Malcovati, Aristoteles Giagounidis, Matteo G Della Porta, Martin Jädersten, Hamid Dolatshad, Amit Verma, Nicholas C. P. Cross, Paresh Vyas, Sally Killick, Eva Hellström-Lindberg, Mario Cazzola, Elli Papaemmanuil, Peter J. Campbell, Jacqueline Boultwood

**Affiliations:** 1 Wellcome Trust Sanger Institute, Hinxton CB10 1SA, UK; 2 LLR Molecular Haematology Unit, NDCLS, RDM, University of Oxford, Oxford OX3 9DU, UK; 3 Department of Hematology Oncology, Fondazione IRCCS Policlinico San Matteo, 27100 Pavia, Italy; 4 Departments of Molecular Medicine and Internal Medicine and Medical Therapy, University of Pavia, 27100 Pavia, Italy; 5 Department of Hematology, Oncology, and Palliative Care, Marienhospital Düsseldorf, 40479 Düsseldorf, Germany; 6 Department of Internal Medicine and Medical Therapy, University of Pavia, 27100 Pavia, Italy; 7 Division of Hematology, Department of Medicine, Karolinska Institutet, SE-171 76 Stockholm, Sweden; 8 Albert Einstein College of Medicine, Bronx, New York 10461, USA; 9 National Genetics Reference Laboratory, Salisbury NHS Foundation Trust, Salisbury SP2 8BJ, UK; 10 MRC Molecular Haematology Unit, Weatherall Institute of Molecular Medicine, University of Oxford, Oxford OX3 9DS, UK; 11 Department of Haematology, Royal Bournemouth Hospital, Bournemouth BH7 7DW, UK

## Abstract

Cancer is a genetic disease, but two patients rarely have identical genotypes. Similarly, patients differ in their clinicopathological parameters, but how genotypic and phenotypic heterogeneity are interconnected is not well understood. Here we build statistical models to disentangle the effect of 12 recurrently mutated genes and 4 cytogenetic alterations on gene expression, diagnostic clinical variables and outcome in 124 patients with myelodysplastic syndromes. Overall, one or more genetic lesions correlate with expression levels of ~20% of all genes, explaining 20–65% of observed expression variability. Differential expression patterns vary between mutations and reflect the underlying biology, such as aberrant polycomb repression for *ASXL1* and *EZH2* mutations or perturbed gene dosage for copy-number changes. In predicting survival, genomic, transcriptomic and diagnostic clinical variables all have utility, with the largest contribution from the transcriptome. Similar observations are made on the TCGA acute myeloid leukaemia cohort, confirming the general trends reported here.

## Abstract (web summary)

The myelodysplastic syndromes (MDS) are a heterogeneous group of chronic blood cancers. Here, the authors analyse genomic and gene expression data from MDS patients to investigate how driver mutations alter gene expression, diagnostic clinical variables and survival.

## Graphical abstract

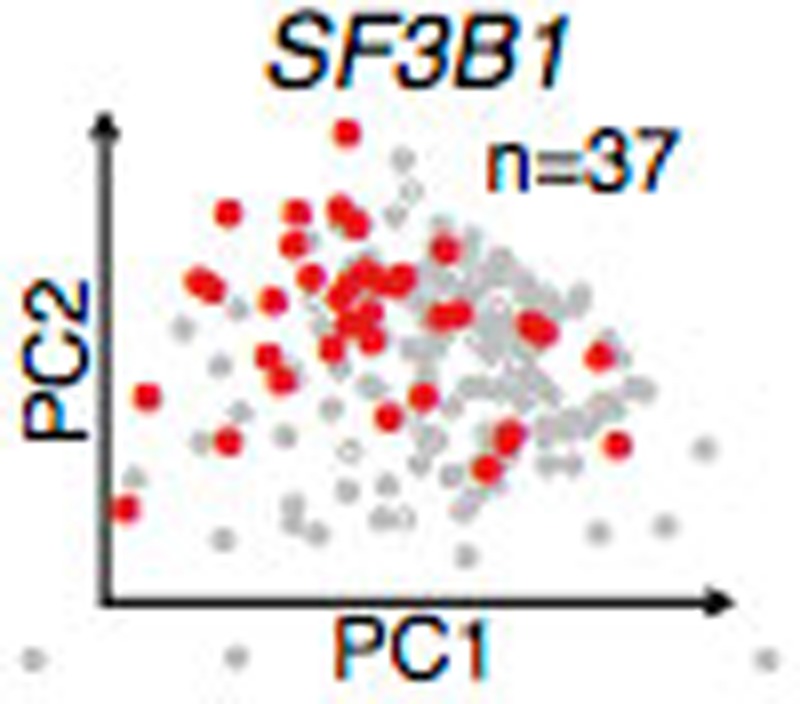

It is well recognized that patients diagnosed with the same cancer often have different constellations of pathological parameters, frequently associated with distinct clinical and prognostic features. In the last decade, molecular testing has increasingly penetrated clinical practice, largely through screening for driver mutations. Gene expression profiling has uncovered many systematic differences between cancer and normal cells and has enabled the definition of new, clinically relevant disease subtypes. From a diagnostic standpoint, such expression profiles are used as ‘biomarkers’, with no intent to address causality: it suffices to correlate with the clinical feature of interest. Genomic information occupies a fundamentally different niche in that the screen assays causal, or driver, genes^1^,^2^. The vehicle by which driver mutations cause cancer, however, is transcription acting through an intricate cellular signalling circuitry that links the genomic variants to the clinical phenotype of a cancer.

The myelodysplastic syndromes (MDS) represent a heterogeneous group of chronic blood cancers. MDS is characterized by ineffective haematopoiesis resulting in peripheral cytopenias, and patients typically have a hypercellular bone marrow^3^,^4^. About 30–40% of patients evolve to acute myeloid leukaemia (AML) over months to years after diagnosis. The molecular pathogenesis of MDS is increasingly well understood, charting the sets of commonly mutated genes, chromosomal aberrations and gene expression changes^5^,^6^,^7^. The most commonly mutated genes in MDS are regulators of RNA splicing and epigenetic modifiers, but signal transduction pathways and transcription factors are also frequent targets^6^,^8^,^9^,^10^,^11^. In addition, MDS is characterized by frequent cytogenetic aberrations such as deletions on the long arms on chromosomes 5, 7 and 20, as well as more complex karyotypes.

Many of the consequences of genetic and cytogenetic alterations will affect gene expression by means of aberrant transcription, epigenetic regulation, cell signalling and gene dosage effects. We and others have identified many deregulated genes and gene pathways in MDS using gene expression profiling^12^,^13^,^14^,^15^,^16^,^17^. A fundamental limitation of these studies is the unknown genetic background of the samples. As the recurrence of mutations is typically lower than 10%, expression changes in such small and unknown subgroups could not be reliably mapped.

We recently published a large mutation screen of 111 cancer genes in 738 MDS patients presenting a comprehensive map of the mutational landscape of myelodysplasia^11^. Here we link these genomic data with gene expression microarray data for 159 MDS cases and 17 normal samples, which extend an existing cohort of 116 MDS cases previously published without mutation data^12^. We deconvolute the expression of genes into contributions stemming from each genetic and cytogenetic mutation, which provides deep insights into how driver mutations interfere with the transcriptomic state. We model the influence of mutations and expression changes on diagnostic clinical variables as well as survival and find that the transcriptome appears to be the most powerful predictor of outcome.

## Results

### Mutation patterns correlate with global gene expression

Variation in gene expression across patients with a particular type of cancer may result from a number of factors. For this study, we were primarily interested in isolating and defining the aggregate effects of driver mutations on the transcriptome, noting that age, sex, germline genetic background and other host factors may also contribute as nuisance factors. We combined data from genomic profiling of 111 relevant cancer genes^11^ with microarray gene expression data from CD34+ bone marrow cells of 159 MDS patients and 17 normal individuals in total. Combined expression and mutation data were available for 124/159 MDS patients (Table 1; Supplementary Data 1). Outcome data were also available and are released with the gene expression data (GEO accession GSE58831). In addition, we release all code associated with implementing the detailed statistical analyses that follow (Supplementary Data 2), in order that this study can be replicated on this data set and extended to other tumour types.

**Figure 1:**
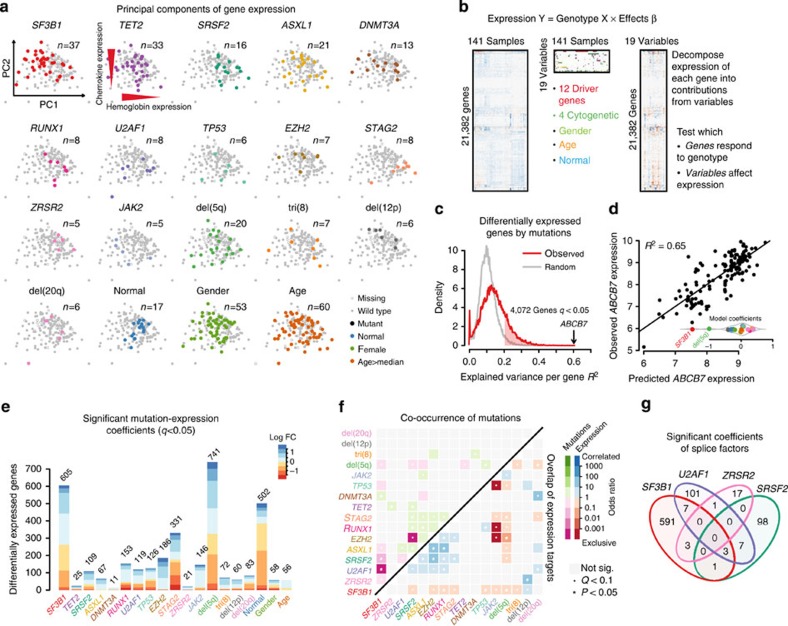
Patterns of mutation and differential expression. (**a**) Scatter plot of the first two principal components of gene expression data of 159 MDS and 17 normal samples overlaid with mutation status of the 12 most frequent point mutations, 4 cytogenetic alterations, as well as normal status, gender and age above or below median (67 years). (**b**) Schematic linear decomposition of expression data by driver mutations and demographic variables. (**c**) Distribution of the variance explained by genetic and cytogenetic alterations across genes (moderated *F*-test; FDR<0.05; *n*=141). (**d**) Scatter plot of expression predictions for the *ABCB7* gene versus observed expression values. The inset shows the model coefficients indicating the predicted magnitudes of expression changes when a given alteration is present. (**e**) Statistically significant mutation expression interaction terms (moderated *t*-test; FDR<0.05; *n*=141) for each alteration and demographic variable. The associated logarithmic expression fold change is indicated by colour. (**f**) Heatmap of observed pairwise mutation patterns (odds ratio; upper triangle) and overlap of differentially expressed genes associated with each alteration (lower triangle). Green/blue colours denote preferential co-mutation/high overlap, while pink/red colours indicate mutual exclusivity. (**g**) Venn diagram of differentially expressed genes associated with spliceosome mutations.

To obtain an overview of the main patterns of expression changes, we first performed a principal components analysis (PCA; Fig. 1a). This multivariate statistical technique collapses multidimensional correlated data such as gene expression of more than 20,000 genes into a smaller set of mutually uncorrelated variables, ordered such that the first few principal components explain the greatest amount of variation in the data. The PCA was computed on all 176 cases with expression data to maximize the stability of the components. In our data, the first two principal components (PCs), respectively, account for 14.4% and 7.8% of the total variability in gene expression; the first 20 PCs cumulatively explain 67% of the variance (Supplementary Fig. 1). The expression changes associated with PC1 are dominated by genes related to haematopoietic differentiation genes; for example, the stem cell factors *KIT*, *CD34* and also *FLT3* have positive values in PC1 while the monocyte-specific antigens *CD14* and *CD163* as well as members of the α and β globin gene clusters have negative values. The second component PC2 had low levels of multiple chemokines and high levels of eosinophil- and neutrophil-related genes as well as the haematopoietic transcription factor *KLF1*. Notably, the observed principal components did not lead to clearly separated groups of patients, but rather to a continuum of expression changes (Fig. 1a).

Strikingly, overlaying the status of 12 recurrent (≥5 patients) genetic and 4 cytogenetic alterations on to the first two principal components demonstrated that driver mutations are correlated with general gene expression profiles (Fig. 1a). For example, patients with mutations in the RNA splicing factor *SF3B1* tend to have low scores on the first principal component, whereas patients with mutations in two other splicing factors, *SRSF2* and *ZRSR2*, have high scores. Similarly, *STAG2* mutations coincide with extremely high values in the first principal component. These general associations, however, do not necessarily imply causation—*STAG2* mutations tend to co-occur with *SRSF2* mutations and more frequently in refractory cytopenia with multilineage dysplasia and refractory anaemia with excess blasts^18^, either of which may explain the correlation with a given PC.

### A linear model to deconvolute gene expression and mutations

To explore predictors of gene expression in a multivariate framework, we developed a linear modelling approach that measures the association of expression levels on a gene-by-gene basis with a number of potential predictors, including driver mutations and nuisance variables (Fig. 1b). The normal samples were included to identify changes common to all MDS samples. Somatically acquired mutations and cytogenetic lesions were encoded as being present/absent. The model assumes that each mutation is associated with a certain set of expression changes and that the expression pattern in cases with a complex genotype comprising multiple alterations is the sum of the changes induced by each mutation. We chose a linear model due to its interpretability and established statistical methods, enabling us to test which transcripts are deregulated in the presence of specific alterations, after correcting for other confounding variables such as the nuisance factors and coexisting driver mutations. The additivity assumption ignores potential interactions between genetic lesions that may arise from the cellular signalling circuitry. However, inferring these interactions systematically from the data would require many more cases, as the number of gene:gene interaction pairs is quadratic and yields combinatorially many terms for higher order interactions. Hence, this assumption appears necessary for statistically robust inference in a data set of this size.

The transcriptome of MDS is globally perturbed by genetic and cytogenetic driver mutations, with expression levels of 4,072/21,382 (19%) genes significantly associated with at least one driver mutation, after correction for multiple hypothesis testing (FDR-adjusted moderated *F*-statistic<0.05; Supplementary Data 3). For these genes, genomic alterations accounted for at least *R*^2^=20.3% of the observed inter-patient gene expression variability; the strongest association of *R*^2^=65% between mutations and expression changes was observed for the iron transporter *ABCB7* (Fig. 1c). The observed variability can be largely explained by the presence of *SF3B1* mutations and del(5q) leading to strong downregulation of *ABCB7* mRNA (Fig. 1d). Inherited missense mutations in *ABCB7* have been linked to hereditary X-linked sideroblastic anaemia and ataxia (OMIM #301310)^19^, and the mechanistic role of *ABCB7* in refractory anaemia with ring sideroblasts (RARS) has been demonstrated^20^. Similarly, somatic *SF3B1* mutations were found to correlate strongly with the presence of ring sideroblasts^21^, and our data suggest an interesting three-way association across *SF3B1* mutation, *ABCB7* downregulation and the occurrence of ring sideroblasts.

Our linear model also allows us to take a mutation-centric view, specifying the set of gene expression changes that correlate with a given driver alteration. The number of target genes whose expression is differentially affected varies widely across the different driver variants. For example, del(5q) is independently correlated with expression levels of 741 genes; there were 605 target genes for patients with *SF3B1* mutations, whereas driver mutations in *TET2* and *DNMT3A* altered expression of only 25 and 11 genes, respectively (Fig. 1e; Supplementary Data 3). These striking differences cannot be explained by variable statistical power, as all four of these genetic lesions are among the six most common driver mutations in MDS^11^. Compared with normal samples, 502 genes change in expression in MDS samples without being attributable to a distinct driver mutation. Finally, the expression of 58 genes correlated with sex, with the most significant effect occurring in the *XIST* gene responsible for X chromosome dosage compensation.

The genomic landscape of myeloid malignancies is characterized by a secondary structure with striking patterns of co-occurrence or mutual exclusivity with other driver genes^11^,^22^,^23^,^24^. The most frequently invoked explanation for a pair of genes exhibiting mutual exclusivity is functional redundancy of the two genes, potentially because they act in the same pathway^25^,^26^. We therefore compared, for each pair of driver mutations, the extent to which their sets of target genes overlapped (Fig. 1f). Gene pairs that tend to be mutated together also show greater degrees of overlap in downstream transcriptional changes than expected by chance, and vice versa for abnormalities that tend to be mutually exclusive. Four genes involved in RNA splicing are mutated in MDS: *SF3B1*, *SRSF2*, *U2AF1* and *ZRSR2*. Despite these genes showing highly significant patterns of mutually exclusive mutation^11^,^23^,^24^, we see a remarkably small overlap in their consequences on gene expression (Fig. 1g). Thus, although mutations in different genes within the same pathway may exhibit similar oncogenic potential, this does not necessarily imply that their transcriptional consequences will be equivalent. Indeed, the lack of overlap between the transcriptional consequences of mutations in different splicing factors may explain why they have such profoundly different clinical phenotypes, with *SF3B1* mutations driving a benign MDS associated with ring sideroblasts and *SRSF2* mutations driving a more aggressive myelomonocytic disease. These data further suggest that the reason mutations in splicing factors tend to be mutually exclusive may not be because they are functionally redundant. It is possible, for example, that more than one mutated splicing gene results in a functional disadvantage.

### Genetic mechanisms of mutation-induced expression changes

**Figure 2:**
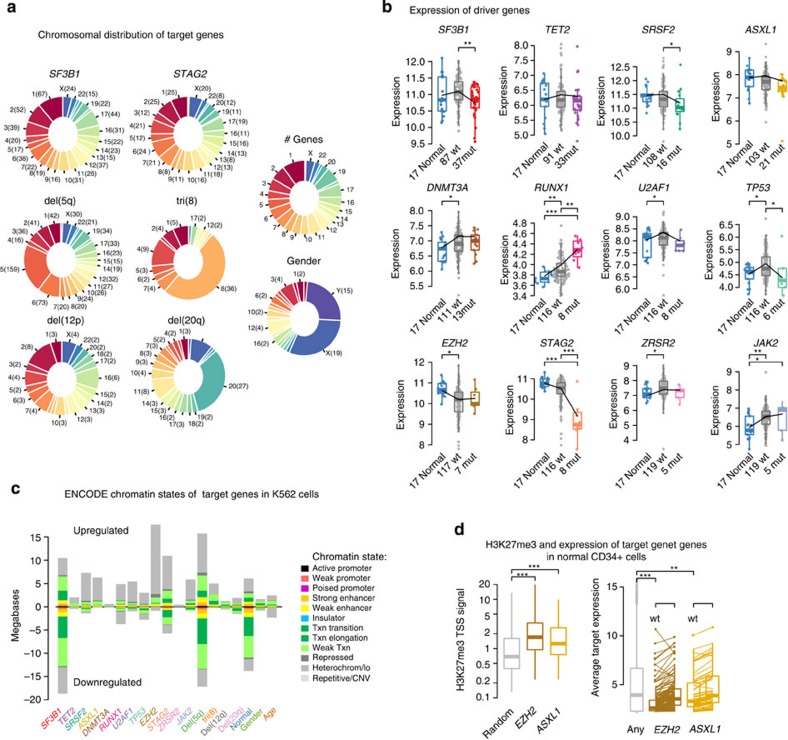
Genetic mechanisms of differential expression. (**a**) Pie charts illustrating the chromosomal locations of differentially expressed genes. (**b**) Expression boxplots of mutated genes in normal, wild-type MDS and MDS samples in which the indicated gene is found to be mutated. The lines denote the expression predicted by the linear model and account for multivariate effects. Stars indicate significance, adjusted to the number of shown comparisons. (***P<10^−3^, **P<10^−2^, *P<10^−1^; FDR-adjusted moderated *t*-test; *n*=141). (**c**) ENCODE chromosome states in K562 myeloid leukaemia cells for differentially expressed genes for each driver alteration. The *y*-axis denotes the sum of the lengths of all differentially expressed genes in Megabases. (**d**) Left: NIH roadmap H3K27me3 ChIP-seq enrichment at transcription start sites of genes differentially expressed in *EZH2* and *ASXL1* mutant MDS. Right: observed expression changes associated with *EZH2* and *ASXL1* compared with 1,000 randomly selected genes.

The nature of differentially expressed genes associated with a given driver abnormality may provide insight into its mode of action. For driver point mutations, the number of differentially expressed genes per chromosome broadly follows the gene density on the autosomes (Fig. 2a). In contrast, for cytogenetic abnormalities, the largest share of expression changes occurs at the deleted or amplified genomic locus as a result of the altered gene dosage. We also find, however, an appreciable set of secondary, *trans* effects on other chromosomes. Reassuringly, sex-specific effects predominantly localized to the X and Y chromosomes.

The expression of the mutated gene itself also carries clues about the pathogenicity and whether alternative mechanisms such as epigenetic silencing or deregulation are acting in those cases where the driver is not mutated. We observed lower expression levels of *SF3B1*, *SRSF2*, *TP53* and *STAG2* in mutated MDS cases compared with wild-type cases, consistent with previous observations^27^ (Fig. 2b). The effect was most striking for *STAG2;* a reduced expression of the STAG2 protein in mutant cases has been reported in myeloid cell lines, but restricted to the chromatin-associated protein fraction^18^. Conversely, *RUNX1* was more strongly expressed when mutated, which may indicate either that *RUNX1* mutations more frequently occur in cell types with high RUNX1 activity, or that *RUNX1* mutations perturb the autocatalytic RUNX1 signalling network^28^.

The ENCODE consortium has provided a rich molecular annotation of the genome in multiple cell types including K562 chronic myeloid leukaemia cells and the Gm12787 lymphoblastoid haematopoietic progenitor cell line. We explored the distribution of genomic states among differentially regulated genes, using ENCODE data on K562 cells^29^ (Fig. 2c). Downregulation typically occurs in transcriptionally active genes, whereas upregulation can also affect genes found to be silenced and heterochromatic. This latter was especially pronounced for the set of transcriptional changes associated with *ASXL1* and *EZH2* mutations. The histone-lysine N-methyltransferase EZH2 is part of the Polycomb Repressive complex 2 (PRC2) and trimethylates histone H3 lysine 27 (H3K27) to silence chromatin; this process is facilitated by ASXL1 (ref. 30). We find that genes deregulated by *EZH2* and *ASXL1* mutations are only weakly expressed in normal cells and show an enrichment of repressive H3K27me3 signal in data of normal CD34+ cells obtained from the NIH epigenome roadmap consortium^31^ (Fig. 2d). Thus, driver mutations in *EZH2* and *ASXL1* lead to a derepression of certain polycomb group target loci leading to an increased expression in mutated cases. The loss of PRC2 repression in *ASXL1* mutant cell lines has been functionally demonstrated in cancer cell lines^32^, as well as in human CD34+ cells following *ASXL1* knockdown^33^, and it is compelling to observe this mechanism in primary patient samples.

### Prediction of blood counts by expression and mutations

Blood counts and bone marrow features are important diagnostic and prognostic variables. In MDS, they are a phenotype, in the sense that they imperfectly reflect the underlying biology of the disease, and hence the genome and transcriptome state. To explore these inter-relationships, we used generalized linear models to quantify the association of common genetic and cytogenetic alterations, as well as the first 20 principal components of the transcriptome, with blood counts, bone marrow blast fractions, myeloid:erythroid ratio, ring sideroblast counts, serum ferritin, sex and age. These models enable us to identify the most important genetic and transcriptomic variables for predicting each variable and measure the aggregated contribution of genetic, cytogenetic and transcriptomic variables.

**Figure 3:**
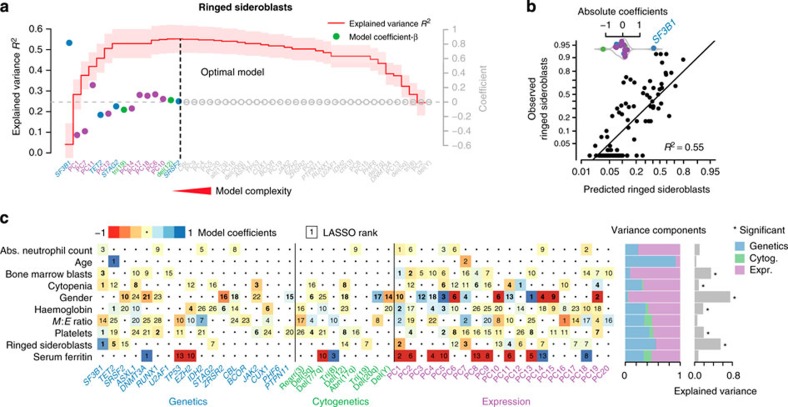
Prediction of blood and bone marrow counts. (**a**) Variance explained by selected driver genes, cytogenetic lesions and first 20 transcriptome principal components (red line±1 s.d.; fivefold cross validation) ordered by their occurrence in a LASSO penalized model. The optimal model maximizes the explained variance *R*^2^. The right axis indicates the effect of each standardized covariate in the optimal model. (**b**) Scatter plot of predicted and observed amounts of ringed sideroblasts on a double logit axis. The inset shows the model coefficients indicating the magnitude of each fold change of driver alterations or a unit fold change in the expression components. (**c**) Heatmap of optimal model coefficients for eight blood and bone marrow counts plus gender and age. LASSO-selected coefficients are coloured. The numbers on each tile denote the order in which variables are included indicating their relative importance. Bold fonts are used for highly significant coefficients in which the explained variance is one s.d. below the maximum. The right bar plot shows the estimated distribution of variance explained by genetic, cytogenetic and transcriptomic variables. Stars (*) denote models where *R*^2^ is greater than zero by a margin of more than one s.d.

We exemplify these analyses with the use of genomic and transcriptomic variables to predict the fraction of ring sideroblasts in the marrow (Fig. 3a). The two strongest predictors were the presence of *SF3B1* mutations and principal component 1 from the gene expression data. Including additional factors further improves the cross-validated predictive accuracy *R*^2^, before starting to overfit, leading to a decline in *R*^2^. The optimal model had a predictive accuracy of *R*^2^=55% indicating a good agreement between predictions and observations (Fig. 3b). As expected, *SF3B1* mutations were positively correlated with the number of ring sideroblasts and, together with the smaller negative contributions of *TET2, STAG2* and *SRSF2* mutations, accounted for 31% of the observed variance (Fig. 3c). A further 25% of the variance was explained by the expression data.

Similarly, we could model 35% of the variability in bone marrow blast counts using PC1, PC2 and *SF3B1* mutations, with additional small contributions from mutations in *ASXL1*, *DNMT3A* and *U2AF1* (Fig. 3c). The largest contribution to the explained variance stemmed from the expression data, in total accounting for 32% of the explained variance, compared with 3% attributed to genetic variables. The residual variance of 100%—*R*^2^=65% indicates that there are fluctuations in the abundance of blast counts that are caused by factors, which are either not included in our data set, such as somatic mutations in genes that have not been sequenced, germline genetics or epigenetics, or of technical nature.

In general, our ability to predict haematological variables from genomic and transcriptomic data varied greatly. We were able to model 18% of variance in haemoglobin levels and 19% of variance in platelet counts, with broadly equal contributions from gene mutation and gene expression data (Fig. 3c). No significant association was found between our data and absolute neutrophil counts, myeloid:erythroid ratio or serum ferritin levels, likely because of the relatively high number of missing cases for the latter two variables that limit our ability to train predictive models (Table 1).

### Prognostic power of expression, mutations and clinical data

An important clinical aspiration is to accurately ascertain a patient’s prognosis. With a high degree of interdependency among genetic, cytogenetic, transcriptomic and haematological variables, it is a challenge to derive the best combination of predictors to calculate a patient’s risk. Currently in clinical practice, the IPSS score is used for prognostication in MDS^34^. Here, we used multivariate Cox proportional hazards models to predict leukaemia-free survival. We were especially interested in the extent of prognostic information contained by different classes of predictor variables, such as genetic or transcriptomic data, as this may provide which data types are best suited for developing novel prediction schemes.

**Figure 4:**
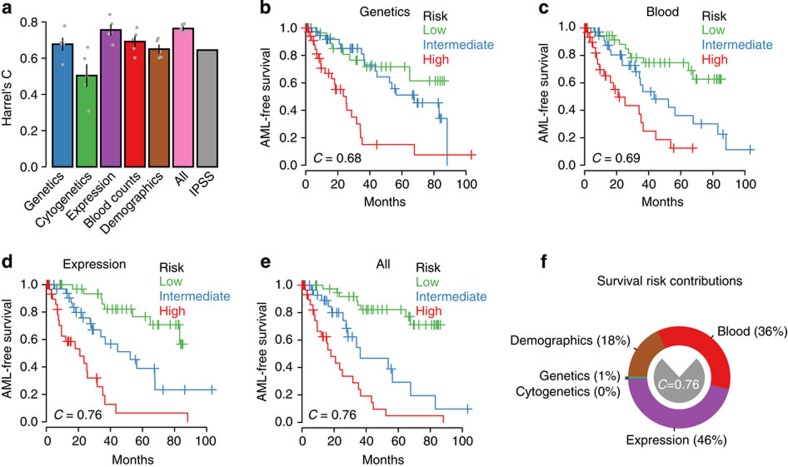
Prediction of survival. (**a**) Barplot of Harrel’s *C*-statistic for multivariate Cox proportional hazards models using genetic, cytogenetic, expression, blood and bone marrow counts, demographics, or all variables as well as IPSS. Values are fivefold cross-validated (grey points) with the bar showing the average across five splits; error bars denote the s.d. of the mean. (**b**–**e**) Kaplan–Meier curves for risk terciles of multivariate survival model for different categories of predictive variables. (**f**) Distribution of risk contributions in the survival model using all covariates. The relative contribution of genetics and cytogenetics is small, despite their marginal predictive potential.

We therefore grouped the available data into five classes of variables: gene mutations, gene expression, cytogenetics, diagnostic blood counts and demographic variables (gender and age). To estimate the predictive accuracy of a given class of predictor variables in an unbiased way, we used fivefold cross-validation using four-fifths of the patients to train a multivariate Cox proportional hazards model. We then apply this to predict outcome on the remaining fifth of the patients, using Harrel’s *C* statistic to measure the concordance between the predicted risk and the observed outcome. A value of *C*=50% is equivalent to random guessing and a value of 100% indicates that the risk ranks the survival times of all patients correctly (Fig. 4a).

First, we separately evaluated the prognostic power of each data type. The accuracy of genetics alone was *C*=68% (Fig. 4b), that of blood counts and bone marrow features was *C*=69% (Fig. 4c), somewhat inferior to that obtained by using expression data (based on the first 20 PCs) of *C*=76% (Fig. 4d). Although we had only a small data set, which generally reduces the cross-validated model accuracy as test and training data splits may differ substantially and also leads to an uncertainty of these estimates in the order of a few percent, it is notable that our models achieved a value greater than the current standard, the IPSS score (*C*=64%). It therefore seems that these data categories individually possess reasonable potential for prognostication that warrants future investigation and validation in larger cohorts.

Combination of the variable classes resulted in a modest increase of the predictive accuracy to *C*=76%, similar to the value obtained by expression data alone (Fig. 4e). Decomposing the contributions to the risk prediction by each category showed that the relative contribution of cytogenetics and genetics did not add measurably to the risk estimates (Fig. 4f). This behaviour was confirmed by using random survival forests as a complementary modelling approach (Supplementary Fig. 2). It therefore appears as if the prognostic information present in genetics and cytogenetics is, at least at the resolution possible with this cohort, mostly contained in expression and blood count data, and does not add independent prognostic information. It may also be that the global transcriptomic changes capture biological variability caused by genomic alterations or other factors, which we have not assessed in our gene screen.

### Validation on TCGA AML data

**Figure 5:**
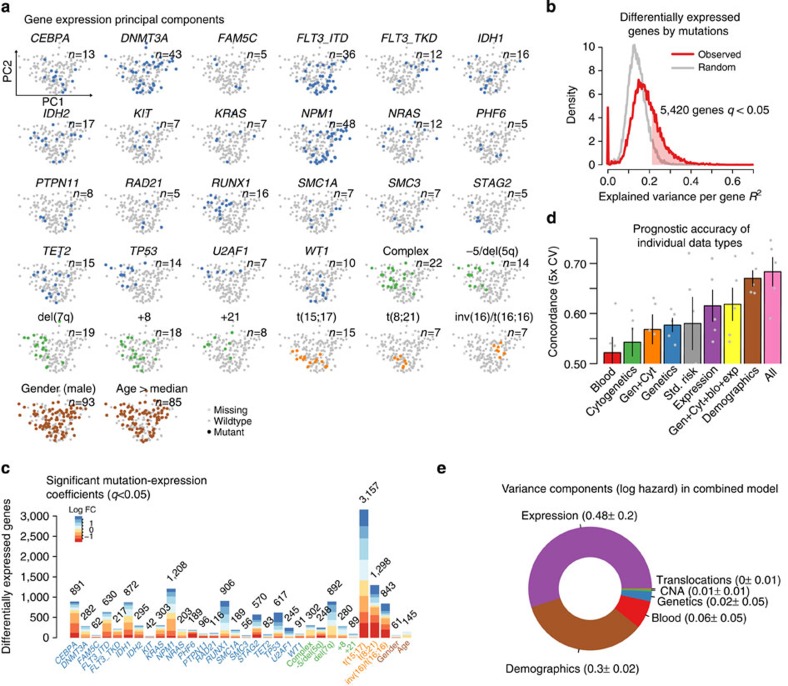
TCGA AML cohort. (**a**) Principal components 1 and 2 of 173 AML cases with RNA-seq data, overlaid by mutation status of 30 recurrent genomic lesions (green: point mutations and small indels; blue: copy-number alterations; orange: balanced translocations) as well as gender (male highlighted in brown) and age (above median of 57.5 years in brown). (**b**) Distribution of gene expression variance explained by genetic and cytogenetic factors (moderated *F*-test; FDR<0.05; *n*=173). (**c**) Number of differentially expressed genes per genetic lesion (moderated *t*-test; FDR<0.05; *n*=173). (**d**) Prognostic accuracy of different data types and their combinations measured by a fivefold cross-validated *C*-statistic. Error bars denote the s.d. of the mean. (**e**) Variance decomposition of the log-hazard predictions in a model comprising all data types. Values denoted by ± are the mean prediction errors in each group.

To investigate whether the observed findings were specific to our cohort of MDS samples and to demonstrate the applicability of our methods to other cancer types, we downloaded RNA-seq, mutations, cytogenetic and clinical data from The Cancer Genome Atlas (TCGA) AML cohort^27^ (Supplementary Data 4 and Supplementary Data 5). Combined data were available for 173 patients and contained mutation data for 30 recurrent (*n*≥5) lesions. The correspondence of gene expression and recurrent alterations was very similar to our observations in MDS. The first principal component of AML gene expression showed a remarkable association with *NPM1* and *FLT3* mutations in samples with high values of PC1; mutations in *TP53* and *RUNX1*, deletions of 5/5q and 7q, and trisomy 8 coincide with low values of PC1. The recurrent balanced translocations t(15;17) and t(8;21) occur predominantly in samples with low levels of PC2 (Fig. 5a).

These descriptive observations translate to 5,420/18,214=30% genes significantly associated with recurrent mutations and explained variances *R*^2^ ranging from 21 to 69% (Fig. 5b). The top 50 genes with the highest *R*^2^ contained many genes of the *HOX* family, which are important regulators of development and haematopoietic differentiation (Supplementary Data 4). The number of associated expression changes per lesion ranged from 42 (*KIT*) to 3,157 for t(15;17), with a general trend of a high number of differentially expressed genes in AML cases with balanced translocations (Fig. 5c).

Prognostic blood counts, demographics, genetics, cytogenetics and gene expression were found to posses prognostic power (Fig. 5d). Although the uncertainty does not allow us to derive definitive conclusions, we observed that the overall accuracy of models fit to genetics and cytogenetics data appeared surprisingly low (*C*=58% and *C*=54%, respectively), close to that of the established cytogenetic risk classification (*C*=58%). The prognostic value of expression data was higher (*C*=62%), but by far the most influential factor for survival in this cohort was patient demographics (age, gender, *C*=67%).

Combing all data types in one prognostic model did not significantly increase the concordance (*C*=68%) with the most influential data components being expression and demographics, confirming our findings in MDS. Taken together these observations support the notion that a substantial proportion of genetic variability translates into distinct transcriptional states, which appear at least as informative for predicting outcome as the genetic data alone.

## Discussion

We have performed a comprehensive analysis to study the relationships between mutations in 12 genes frequently mutated in MDS, common cytogenetic aberrations, gene expression profiles from bone marrow CD34+ cells, diagnostic clinical variables as well as outcome in 124 MDS patients. Our analysis allowed for systematically testing for associations across driver alterations, expression changes, clinical variables and outcome.

Some of the findings revealed by our systematic analysis confirm known mechanisms, while other findings are more surprising. First of all, it was somewhat unexpected that the number of differentially expressed genes in MDS varied so extensively among driver alterations (11 for *DNMT3A* to 741 in del(5q)). The ability to detect transcription changes attributable to a driver mutation may depend on the number of cases with that alteration, although we did not observe a general trend in our data. The second largest number of differentially expressed genes was, for example, associated with *STAG2* mutations, which were observed in only eight cases. Analysing larger cohorts will refine these estimates and should generally increase the number of significant expression changes as more subtle differences can be detected.

When analysing observational data, it is generally difficult to discern the underlying causality. Here, that means that the associated differences in expression patterns between different mutant genotypes could, in principle, also indicate that mutations occur preferentially in certain cell types. Ultimately, the given data do not allow us to rule out this possibility. For many examples discussed here, however, there exists additional evidence from functional experiments illustrating the mechanistic link between mutations and transcription changes, such as the association between *SF3B1* mutations, *ABCB7* downregulation and a sideroblastic phenotype, or the loss of PRC repression at *ASXL1* and *EZH2* target genes.

**Figure 6:**
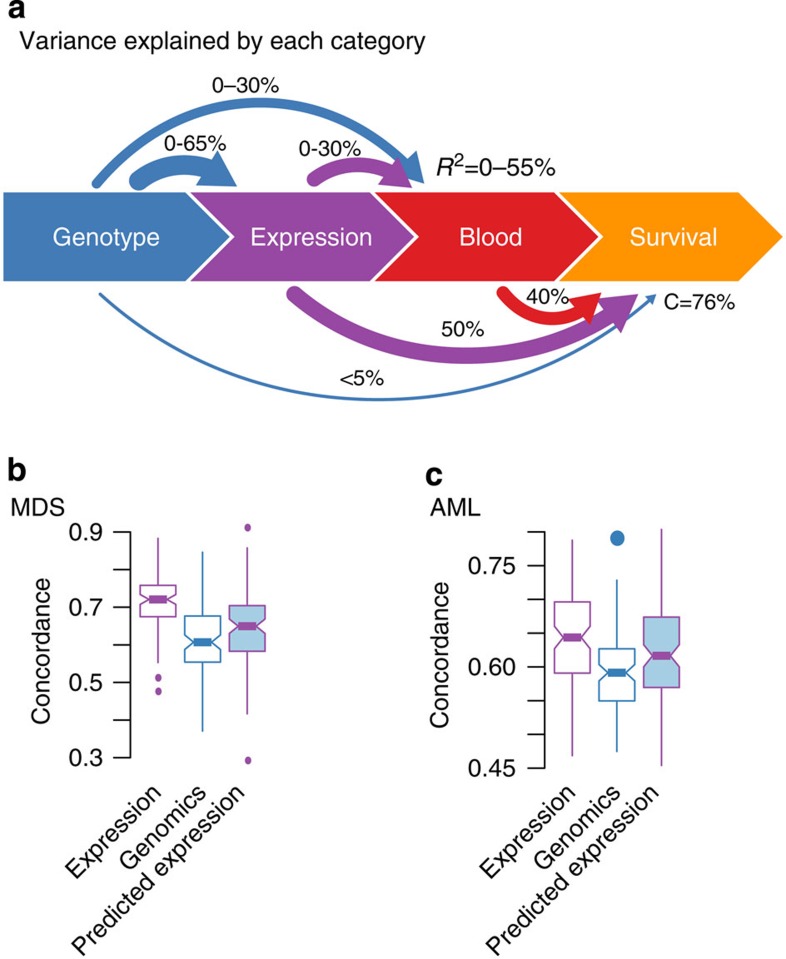
Summary of findings. (**a**) Flow chart indicating explained variance in MDS for different associations from genotype to survival. Values on each arrow are the relative contribution to the explained variance stemming from the variable category on the foot of the arrow in multivariate model predicting the variable at the arrowhead using all incoming variables. We observe a tendency to find stronger associations between successive categories than between distal ones. (**b**,**c**) Prognostic value of observed and predicted expression data and genomics in MDS and AML. Shown are results from 100 test/training splits fitting a model to 4/5 of the data and evaluating the predictions on the remainder.

Overall, our results can be broadly summarized as in Fig. 6a: there is a considerable influence of the genotype on expression data accounting for up to 65% of the observed expression variability in some genes, which is remarkable given the technical noise found in gene expression data. Both genotype and expression data have a similar predictive contribution for modelling blood counts. Genetics, clinical diagnostic variables and demographics were all found to contain information for predicting survival. When combining all available data types in a multivariable survival model, prognostic accuracy is greater than that based on individual data sets. This indicates that the accuracy of prognostic models for MDS can benefit from incorporating multiple data types, including gene expression data. The main contributions to predict risk were attributed to expression and blood counts; the contribution of genetics and cytogenetics was smaller.

Very similar phenomena were observed when analysing the TCGA AML cohort, where a comparable number and strength of associations between gene expression and mutations were found. When analysing outcome, we again observed that expression data appeared at least as powerful in predicting survival as mutation data, which is surprising given the established prognostic importance of genetics and cytogenetics in AML.

Although it is generally difficult to disentangle the effect of multicollinear covariates, our findings are compatible with the notion of a hierarchy in which the driver mutations in the genome dictate intermediate phenotypes, such as gene expression and blood counts, which ultimately determine outcome (Fig. 6a). Hence, the genome indirectly controls survival, and this information is largely contained in the intermediate phenotypes, making these influential proxies for outcome. Conversely, understanding the interrelationship of genotype and phenotypes may help decipher the prognostic information that is genetically encoded. If we, in hindsight, compute the predicted values of the expression principal components, based on the linear expression models defined earlier (using only genomic variables), we observe an improved accuracy in outcome predictions compared with pure genomic data (Fig. 6b,c). It thus appears as if mapping the genotypes to expression data accentuates the genetic data such that the prognostic information becomes easier to extract. The observed expression data, however, still seems to be slightly superior in its predictive performance, likely as it comprises the effects of many more somatic and germline variants than the 17 mutations that were analysed here.

We would expect that more comprehensive sequencing data and a better understanding of the underlying genetic and epigenetic mechanisms will allow us to explain a large fraction of the residual phenotypic variability and ultimately also to more accurately predict survival. In this analysis we have not utilized that mutations are sometimes present in only a subset of cells, which may explain part of the observed phenotypic variability. The reason for this was that individual estimates of the variant allele frequencies of point mutations can be unreliable and were not available for cytogenetic lesions. With whole-genome or single-cell sequencing data one will be able to more precisely reconstruct the clonal architecture of bone marrow cells; accounting for subclonal heterogeneity may then increase the fraction of explained phenotypic variance even further.

Transcriptomic and phenotypic heterogeneity in MDS has been recognized for many years, but it has been unclear what the genetic roots of this inter-patient variability are. Here we have systematically decomposed the relation between genetic and cytogenetic alterations, gene expression, blood and bone marrow counts and survival. As we move towards integrating genomic and transcriptional screens into the clinical management of patients with cancer, these interconnected streams of data will require careful modelling to ensure optimal predictive performance.

## Methods

### Samples

The study was approved by the ethics committees (Oxford C00.196, Bournemouth 9991/03/E, Duisburg 2283/03, Stockholm 410/03, Pavia 26264/2002) and informed patient consent was obtained. A total of 159 MDS samples and 17 healthy controls were studied; no samples were excluded in the statistical analysis. Gene expression data of 43 bone marrow samples from MDS patients without published expression data were obtained using the protocol described in ref. 12. In brief, CD34+ cells were enriched from mononuclear cells using CD34 MicroBeads (Miltenyi Biotec, Bergisch Gladbach, Germany). RNA was extracted using TRIZOL (Invitrogen, Paisley, UK). Fifty nanograms of RNA was subsequently amplified and biotin labelled. A total of 10 μg of labelled cRNA was hybridized to Affymetrix GeneChip Human Genome U133 Plus 2.0 arrays (Affymetrix, Santa Clara, CA, USA), which were scanned on an Affymetrix GeneChip Scanner 3000 (ref. 12).

### Statistical computations

All calculations were performed using R version 3.0.1 (ref. 35). A detailed report of all analysis steps can be found in Supplementary Data 2 and 4.

### Modelling gene expression

Probe intensity values were normalized using the gcrma bioconductor package. Normalized probe intensities were then average for each gene in the case of multiple probes. Principal components were computed using the prcomp() function.

Linear expression models were fit with the limma bioconductor package^36^. Here the expression of gene *k* in patient *i*, *Y*_*ik*_ is modelled by the following equation




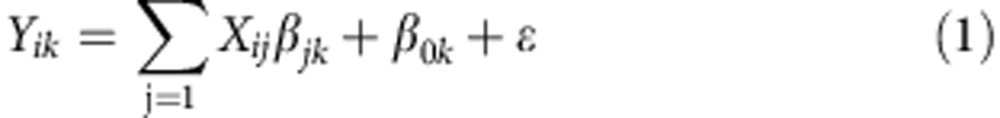




*X*_*ij*_ is the mutation matrix for patient *i* and mutation *j*, with entries *X*_*ij*_=1 indicating that patient *i* has an oncogenic mutation *j* and 0 otherwise. Similarly, for *j* being gender *X*_*ij*_=1 denotes female sex; for *j* being age *X*_*ij*_ takes integer values. The coefficients *β*_*jk*_ measure the expression change in gene *k* induced by the presence of a mutation j. The entry *β*_0*k*_ denotes the baseline expression level of gene *k*. The symbol *p* denotes the number of covariates.

Using this model, finding significant effects amounts to testing whether the coefficients *β*_*jk*_ are different from zero. The limma package uses a moderated *t*-statistic for these tests with a prior variance shared across genes. Similarly, one can test for an overall association of any variable with a given gene using a moderated *F*-test. We used the Benjamini-Hochberg correction for multiple testing^37^. The accuracy of the applied tests and correction schemes was verified using a permutation approach in which each covariate was randomly permuted thereby breaking all correlations between genotype and expression.

### Modelling blood counts

Blood and bone marrow counts were modelled by regularized generalized linear models using the glmnet R package^38^,^39^. The approach is to model the value of the blood count *k*, *Z*_*ik*_ in patient *i* by the generalized linear function




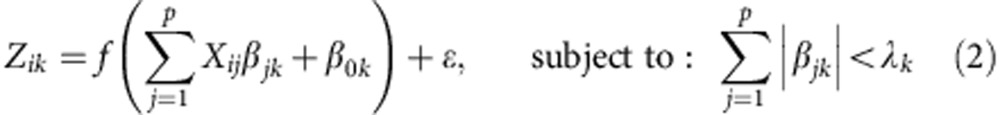




The function *f* is a transformation depending on the range of **Z**_.*k*_, denoting the *k*-th column of the matrix **Z**. For real **Z**_.*k*_ it is the identity, for positive **Z**_.*k*_ the logarithm, and for **Z**_.*k*_ in [0,1] or dichotomous **Z**_.*k*_ it is the logit transform. The optimal penalty *λ*_*k*_ was chosen to be the value maximizing the fivefold cross-validated generalized coefficient of determination *R*_*k*_^2^, which is defined as




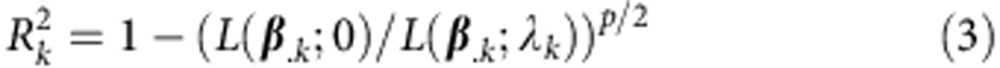




where *L*(***β***_.*k*_;*λ*_*k*_) denotes the likelihood function.

### Survival models

Survival models were fitted using the Cox proportional hazards model^40^. Here the hazard is modelled by the function




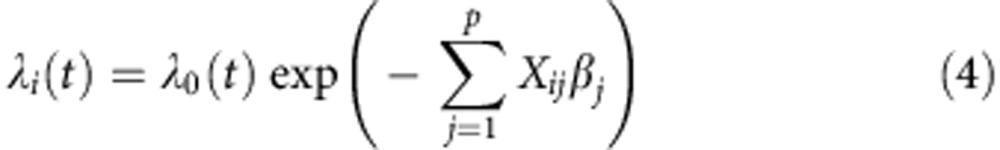




The parameters *β*_*j*_ quantify the changes in the hazard rate imposed by mutation *j*. To increase the stability we used a shared prior on the variances of the risk coefficients.

We evaluated the prognostic accuracy of survival models using Harrel’s *C* statistic, as implemented in the Hmisc R package^41^. This statistic measures the fraction of pairs of patients with concordant risk predictions, exp(–Σ_*j*=1_^*p*^ *X*_*ij*_ *β*_*j*_), and outcome similarly to the area under the receiver operating characteristic curve. To reduce the bias on the estimated risk, we used a fivefold cross-validating scheme, in which the data is split into five parts of approximately same size. Repeating five times, one part of the data was left out for training the model and the *C* statistic was evaluated on the set aside test partition. From the five resulting estimates of *C* we then report the average.

Suppose the *p* covariates can be partitioned into *g* groups, such as genetics, cytogenetics, transcriptomics, and so on. The risk (log hazard) *r*_i_ of patient i can be decomposed into contributions from each group,




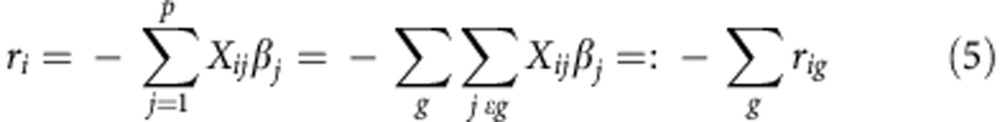




In general the risk components *r*_*ig*_ are correlated and the variance of the risk **r**. (taken across patients) cannot be decomposed into positive variance components. However, we may write




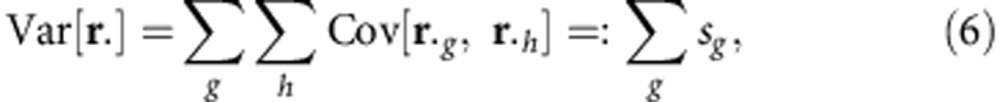




where *s*_*g*_=Σ_*h*_ Cov[**r**_.*g*_, **r**_.*h*_] is a generalized variance component of **r**, which reduces to *s*_*g*_=Var[**r**_.g_] if the covariance matrix Cov[**r**_.*g*_, **r**_.*h*_] is diagonal. The interpretation of *s*_*g*_ is that it measures the residual variance component stemming from group *g* plus correlations from other groups.

Random survival forests were used as an alternative approach for predicting outcome and measuring variable importance^42^. These are implemented in the randomForestSRC R package.

## Additional information

**Accession codes**: Gene expression array data for 176 MDS and normal samples have been deposited in the Gene Expression Omnibus under the accession code GSE58831.

**How to cite this article:** Gerstung, M. *et al.* Combining gene mutation with gene expression data improves outcome prediction in myelodysplastic syndromes. *Nat. Commun.* 6:5901 doi: 10.1038/ncomms6901 (2015).

## Supplementary Material

Supplementary Figures Supplementary Figures 1-2

Supplementary Data 1 Clinical and sequencing data

Supplementary Data 2 MDS analysis report

Supplementary Data 3 Gene-level expression fold changes and test results

Supplementary Data 4 TCGA AML analysis report

Supplementary Data 5 Curated TCGA AML data

## Figures and Tables

**Table 1:** Clinical characteristics of the cohort.

*Samples*	
MDS with expression data	159
MDS with sequencing data	124/159
Normals	17
	
*Demographics*	
Gender	102 male; 57 female
Age	67 years median; 19–87 years range; 5 missing
	
*MDS classification*	
RA	13
RARS	14
RARS-T	6
RCMD	27
RCMD-RS	22
RAEB	56
5q-	6
CMML	7
MDS-AML	7
missing	1
	
*Blood and bone marrow counts at diagnosis*	
Haemoglobin	9.5 g dl^−1^ median; 4.5–14.6 g dl^−1^ range; 15 missing
Platelets	154 × 10^9^ l^−1^ median; 10–45,000 × 10^9^ l^−1^ range; 8 missing
Absolute neutrophil count	1.8 × 10^9^ l^−1^ median; 0.08–920 × 10^9^ l^−1^ range; 20 missing
Bone marrow blasts	4% median; 0–63% range; 17 missing
Ring sideroblasts	0.5% median; 0–94% range; 31 missing
Serum ferritin	543 ng ml^−1^ median; 8–11,300 ng ml^−1^ range; 64 missing
M:E ratio	2.03 median; 0.33–9 range; 70 missing
	
*Outcome and follow-up*	
Follow up time	1,040 days median; 0–3,141 days range; 36 missing
Outcome	82 alive; 41 died; 36 missing
AML transformation	14 positive; 101 negative; 44 missing

*Mutations*					
Number of recurrent cytogenetic lesions per patient	**0**	**1**	**2**	**3**	**Missing**
	81	33	3	0	7
Number of recurrent point mutations and indels per patient	**0**	**1**	**2**	**3**	**4**
	31	42	30	19	2
Variant allele fraction of point mutations and indels	**Min**	**25%**	**50%**	**75%**	**Max**
	0.04	0.27	0.38	0.45	0.97
