# Supplementary material for: Combining gene mutation with gene expression data improves outcome prediction in myelodysplastic syndromes
Source: Nat Commun. 2015 Jan 9;6:5901. doi: 10.1038/ncomms6901 (PMC4338540; doi:10.1038/ncomms6901)
Supplement: Supplementary Figures — 1-2 [file ncomms6901-s1.pdf]

Supplementary Information

Supplementary Figure 1

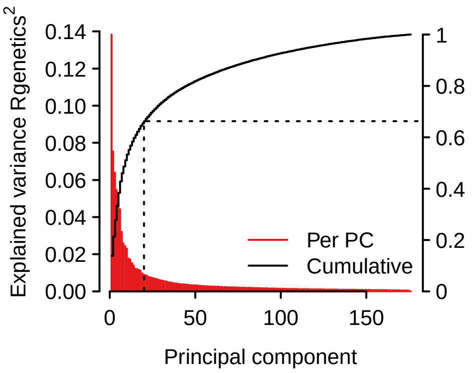

Explained variance (red; left axis) as a function of principal components (PCs) 1-176. The cumulative value is shown by the black curve (right axis). The first 20 PCs account for 67% of the total variation.

Supplementary Figure 2

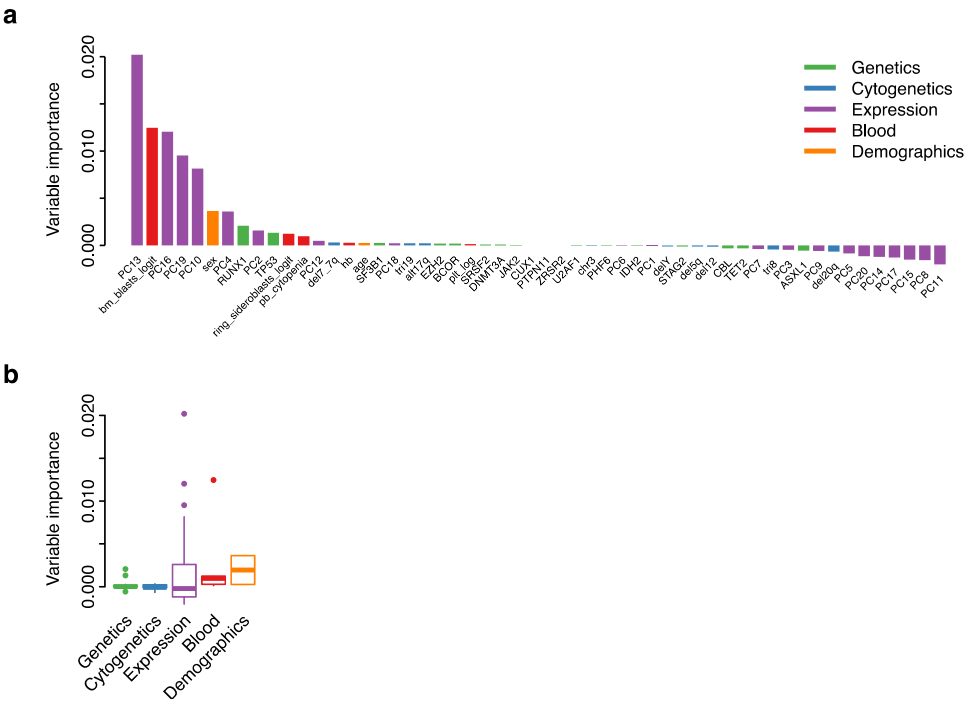

Random survival forest analysis. (a) Variable importance (increase in prediction error after permutation) for all covariates included in the model. Colors indicate the type of variable. Boxplot of the variable importance shown in (a), grouped by variable type.
