# Supplementary material for: Combining gene mutation with gene expression data improves outcome prediction in myelodysplastic syndromes
Source: Nat Commun. 2015 Jan 9;6:5901. doi: 10.1038/ncomms6901 (PMC4338540; doi:10.1038/ncomms6901)
Supplement: Supplementary Data 2 — MDS analysis report [file ncomms6901-s3.zip › ncomms6901-s3.html]

Supplementary Data 2


# Supplementary Data 2

### Supplementary code and figures accompanying *Combining gene mutation with gene expression data improves outcome prediction in myelodysplastic syndromes*

This document contains the complete code used in the analysis. It is purely written in `R` using a series of `R` and `Bioconductor` packages.
This report has been generated using the `knitr` R package (http://yihui.name/knitr/).
For a complete list of packages and their versions please have a look at the end of this document.

### Libraries

Load necessary libraries

```
library(limma)
library(org.Hs.eg.db)
```

```
## Loading required package: AnnotationDbi
## Loading required package: BiocGenerics
## Loading required package: parallel
## 
## Attaching package: 'BiocGenerics'
## 
## The following objects are masked from 'package:parallel':
## 
##     clusterApply, clusterApplyLB, clusterCall, clusterEvalQ, clusterExport, clusterMap, parApply,
##     parCapply, parLapply, parLapplyLB, parRapply, parSapply, parSapplyLB
## 
## The following object is masked from 'package:limma':
## 
##     plotMA
## 
## The following object is masked from 'package:stats':
## 
##     xtabs
## 
## The following objects are masked from 'package:base':
## 
##     anyDuplicated, append, as.data.frame, as.vector, cbind, colnames, duplicated, eval, evalq, Filter,
##     Find, get, intersect, is.unsorted, lapply, Map, mapply, match, mget, order, paste, pmax, pmax.int,
##     pmin, pmin.int, Position, rank, rbind, Reduce, rep.int, rownames, sapply, setdiff, sort, table, tapply,
##     union, unique, unlist
## 
## Loading required package: Biobase
## Welcome to Bioconductor
## 
##     Vignettes contain introductory material; view with 'browseVignettes()'. To cite Bioconductor, see
##     'citation("Biobase")', and for packages 'citation("pkgname")'.
## 
## Loading required package: DBI
```

```
library(RColorBrewer)
library(AnnotationDbi)
library(affy)
library(gcrma)
library(hgu133plus2.db )
```

```
##
```

```
library(VennDiagram)
```

```
## Loading required package: grid
```

```
library(org.Hs.eg.db)
library(GenomicRanges)
```

```
## Loading required package: IRanges
## Loading required package: XVector
```

```
library(GenomicFeatures)
library(rtracklayer)
library(biomaRt)
library(glmnet)
```

```
## Loading required package: Matrix
## 
## Attaching package: 'Matrix'
## 
## The following object is masked from 'package:IRanges':
## 
##     expand
## 
## Loaded glmnet 1.9-8
```

```
library(survival)
```

```
## Loading required package: splines
```

```
library(Hmisc)
```

```
## Loading required package: lattice
## Loading required package: Formula
## 
## Attaching package: 'Hmisc'
## 
## The following object is masked from 'package:AnnotationDbi':
## 
##     contents
## 
## The following objects are masked from 'package:Biobase':
## 
##     combine, contents
## 
## The following object is masked from 'package:BiocGenerics':
## 
##     combine
## 
## The following objects are masked from 'package:base':
## 
##     format.pval, round.POSIXt, trunc.POSIXt, units
```

```
library(randomForestSRC)
```

```
## 
##  randomForestSRC 1.4 
##  
##  Type rfsrc.news() to see new features, changes, and bug fixes. 
##
```

```
set1 = c(brewer.pal(9,"Set1"), brewer.pal(8, "Dark2"))
source("suppData/mg14.R")
```

## 1. Data preprocessing

### 1. Normalisation of expression data

This uses the `affy` package and `gcrma` correction. Pretty standard
We assume you have downloaded the complete tarball GSE58831\_RAW.tar from GEO http://www.ncbi.nlm.nih.gov/geo/query/acc.cgi?acc=GSE58831
and untared the archive and gunzipped all .CEL files into directory `GSE58831`.

```
celFiles <- dir("GSE58831", pattern = ".CEL", full.names = T)
celFiles
```

```
##   [1] "GSE58831/GSM1420393_MDS009.CEL" "GSE58831/GSM1420394_MDS011.CEL" "GSE58831/GSM1420395_MDS012.CEL"
##   [4] "GSE58831/GSM1420396_MDS014.CEL" "GSE58831/GSM1420397_MDS015.CEL" "GSE58831/GSM1420398_MDS025.CEL"
##   [7] "GSE58831/GSM1420399_MDS027.CEL" "GSE58831/GSM1420400_MDS029.CEL" "GSE58831/GSM1420401_MDS184.CEL"
##  [10] "GSE58831/GSM1420402_MDS185.CEL" "GSE58831/GSM1420403_MDS186.CEL" "GSE58831/GSM1420404_MDS187.CEL"
##  [13] "GSE58831/GSM1420405_MDS188.CEL" "GSE58831/GSM1420406_MDS189.CEL" "GSE58831/GSM1420407_MDS033.CEL"
##  [16] "GSE58831/GSM1420408_MDS043.CEL" "GSE58831/GSM1420409_MDS050.CEL" "GSE58831/GSM1420410_MDS052.CEL"
##  [19] "GSE58831/GSM1420411_MDS053.CEL" "GSE58831/GSM1420412_MDS190.CEL" "GSE58831/GSM1420413_MDS054.CEL"
##  [22] "GSE58831/GSM1420414_MDS055.CEL" "GSE58831/GSM1420415_MDS057.CEL" "GSE58831/GSM1420416_MDS058.CEL"
##  [25] "GSE58831/GSM1420417_MDS059.CEL" "GSE58831/GSM1420418_MDS191.CEL" "GSE58831/GSM1420419_MDS061.CEL"
##  [28] "GSE58831/GSM1420420_MDS062.CEL" "GSE58831/GSM1420421_MDS063.CEL" "GSE58831/GSM1420422_MDS066.CEL"
##  [31] "GSE58831/GSM1420423_MDS067.CEL" "GSE58831/GSM1420424_MDS068.CEL" "GSE58831/GSM1420425_MDS069.CEL"
##  [34] "GSE58831/GSM1420426_MDS071.CEL" "GSE58831/GSM1420427_MDS072.CEL" "GSE58831/GSM1420428_MDS074.CEL"
##  [37] "GSE58831/GSM1420429_MDS075.CEL" "GSE58831/GSM1420430_MDS076.CEL" "GSE58831/GSM1420431_MDS079.CEL"
##  [40] "GSE58831/GSM1420432_MDS080.CEL" "GSE58831/GSM1420433_MDS081.CEL" "GSE58831/GSM1420434_MDS082.CEL"
##  [43] "GSE58831/GSM1420435_MDS084.CEL" "GSE58831/GSM1420436_MDS085.CEL" "GSE58831/GSM1420437_MDS087.CEL"
##  [46] "GSE58831/GSM1420438_MDS088.CEL" "GSE58831/GSM1420439_MDS089.CEL" "GSE58831/GSM1420440_MDS090.CEL"
##  [49] "GSE58831/GSM1420441_MDS091.CEL" "GSE58831/GSM1420442_MDS092.CEL" "GSE58831/GSM1420443_MDS094.CEL"
##  [52] "GSE58831/GSM1420444_MDS096.CEL" "GSE58831/GSM1420445_MDS097.CEL" "GSE58831/GSM1420446_MDS098.CEL"
##  [55] "GSE58831/GSM1420447_MDS099.CEL" "GSE58831/GSM1420448_MDS101.CEL" "GSE58831/GSM1420449_MDS102.CEL"
##  [58] "GSE58831/GSM1420450_MDS103.CEL" "GSE58831/GSM1420451_MDS104.CEL" "GSE58831/GSM1420452_MDS106.CEL"
##  [61] "GSE58831/GSM1420453_MDS107.CEL" "GSE58831/GSM1420454_MDS108.CEL" "GSE58831/GSM1420455_MDS113.CEL"
##  [64] "GSE58831/GSM1420456_MDS114.CEL" "GSE58831/GSM1420457_MDS115.CEL" "GSE58831/GSM1420458_MDS116.CEL"
##  [67] "GSE58831/GSM1420459_MDS117.CEL" "GSE58831/GSM1420460_MDS118.CEL" "GSE58831/GSM1420461_MDS120.CEL"
##  [70] "GSE58831/GSM1420462_MDS121.CEL" "GSE58831/GSM1420463_MDS123.CEL" "GSE58831/GSM1420464_MDS124.CEL"
##  [73] "GSE58831/GSM1420465_MDS192.CEL" "GSE58831/GSM1420466_MDS193.CEL" "GSE58831/GSM1420467_MDS194.CEL"
##  [76] "GSE58831/GSM1420468_MDS195.CEL" "GSE58831/GSM1420469_MDS196.CEL" "GSE58831/GSM1420470_MDS197.CEL"
##  [79] "GSE58831/GSM1420471_MDS198.CEL" "GSE58831/GSM1420472_MDS199.CEL" "GSE58831/GSM1420473_MDS200.CEL"
##  [82] "GSE58831/GSM1420474_MDS201.CEL" "GSE58831/GSM1420475_MDS202.CEL" "GSE58831/GSM1420476_MDS203.CEL"
##  [85] "GSE58831/GSM1420477_MDS204.CEL" "GSE58831/GSM1420478_MDS125.CEL" "GSE58831/GSM1420479_MDS205.CEL"
##  [88] "GSE58831/GSM1420480_MDS206.CEL" "GSE58831/GSM1420481_MDS207.CEL" "GSE58831/GSM1420482_MDS208.CEL"
##  [91] "GSE58831/GSM1420483_MDS127.CEL" "GSE58831/GSM1420484_MDS209.CEL" "GSE58831/GSM1420485_MDS128.CEL"
##  [94] "GSE58831/GSM1420486_MDS129.CEL" "GSE58831/GSM1420487_MDS130.CEL" "GSE58831/GSM1420488_MDS131.CEL"
##  [97] "GSE58831/GSM1420489_MDS132.CEL" "GSE58831/GSM1420490_MDS133.CEL" "GSE58831/GSM1420491_MDS210.CEL"
## [100] "GSE58831/GSM1420492_MDS134.CEL" "GSE58831/GSM1420493_MDS135.CEL" "GSE58831/GSM1420494_MDS136.CEL"
## [103] "GSE58831/GSM1420495_MDS138.CEL" "GSE58831/GSM1420496_MDS139.CEL" "GSE58831/GSM1420497_MDS140.CEL"
## [106] "GSE58831/GSM1420498_MDS141.CEL" "GSE58831/GSM1420499_MDS142.CEL" "GSE58831/GSM1420500_MDS143.CEL"
## [109] "GSE58831/GSM1420501_MDS144.CEL" "GSE58831/GSM1420502_MDS211.CEL" "GSE58831/GSM1420503_MDS212.CEL"
## [112] "GSE58831/GSM1420504_MDS213.CEL" "GSE58831/GSM1420505_MDS214.CEL" "GSE58831/GSM1420506_MDS215.CEL"
## [115] "GSE58831/GSM1420507_MDS216.CEL" "GSE58831/GSM1420508_MDS217.CEL" "GSE58831/GSM1420509_MDS218.CEL"
## [118] "GSE58831/GSM1420510_MDS219.CEL" "GSE58831/GSM1420511_MDS220.CEL" "GSE58831/GSM1420512_MDS146.CEL"
## [121] "GSE58831/GSM1420513_MDS147.CEL" "GSE58831/GSM1420514_MDS148.CEL" "GSE58831/GSM1420515_MDS149.CEL"
## [124] "GSE58831/GSM1420516_MDS150.CEL" "GSE58831/GSM1420517_MDS152.CEL" "GSE58831/GSM1420518_MDS151.CEL"
## [127] "GSE58831/GSM1420519_MDS153.CEL" "GSE58831/GSM1420520_MDS221.CEL" "GSE58831/GSM1420521_MDS222.CEL"
## [130] "GSE58831/GSM1420522_MDS155.CEL" "GSE58831/GSM1420523_MDS156.CEL" "GSE58831/GSM1420524_MDS157.CEL"
## [133] "GSE58831/GSM1420525_MDS223.CEL" "GSE58831/GSM1420526_MDS158.CEL" "GSE58831/GSM1420527_MDS159.CEL"
## [136] "GSE58831/GSM1420528_MDS160.CEL" "GSE58831/GSM1420529_MDS161.CEL" "GSE58831/GSM1420530_MDS162.CEL"
## [139] "GSE58831/GSM1420531_MDS163.CEL" "GSE58831/GSM1420532_MDS224.CEL" "GSE58831/GSM1420533_MDS225.CEL"
## [142] "GSE58831/GSM1420534_MDS164.CEL" "GSE58831/GSM1420535_MDS165.CEL" "GSE58831/GSM1420536_MDS166.CEL"
## [145] "GSE58831/GSM1420537_MDS167.CEL" "GSE58831/GSM1420538_MDS168.CEL" "GSE58831/GSM1420539_MDS169.CEL"
## [148] "GSE58831/GSM1420540_MDS170.CEL" "GSE58831/GSM1420541_MDS171.CEL" "GSE58831/GSM1420542_MDS172.CEL"
## [151] "GSE58831/GSM1420543_MDS173.CEL" "GSE58831/GSM1420544_MDS174.CEL" "GSE58831/GSM1420545_MDS176.CEL"
## [154] "GSE58831/GSM1420546_MDS226.CEL" "GSE58831/GSM1420547_MDS178.CEL" "GSE58831/GSM1420548_MDS179.CEL"
## [157] "GSE58831/GSM1420549_MDS180.CEL" "GSE58831/GSM1420550_MDS181.CEL" "GSE58831/GSM1420551_MDS183.CEL"
## [160] "GSE58831/GSM1420552_NBM01.CEL"  "GSE58831/GSM1420553_NBM02.CEL"  "GSE58831/GSM1420554_NBM03.CEL" 
## [163] "GSE58831/GSM1420555_NBM04.CEL"  "GSE58831/GSM1420556_NBM05.CEL"  "GSE58831/GSM1420557_NBM06.CEL" 
## [166] "GSE58831/GSM1420558_NBM07.CEL"  "GSE58831/GSM1420559_NBM08.CEL"  "GSE58831/GSM1420560_NBM09.CEL" 
## [169] "GSE58831/GSM1420561_NBM10.CEL"  "GSE58831/GSM1420562_NBM11.CEL"  "GSE58831/GSM1420563_NBM12.CEL" 
## [172] "GSE58831/GSM1420564_NBM13.CEL"  "GSE58831/GSM1420565_NBM14.CEL"  "GSE58831/GSM1420566_NBM15.CEL" 
## [175] "GSE58831/GSM1420567_NBM16.CEL"  "GSE58831/GSM1420568_NBM17.CEL"
```

```
affyBatch <- read.affybatch(filenames = celFiles)
```

```
## Warning: Incompatible phenoData object. Created a new one.
```

```
gset = gcrma(affyBatch)
```

```
## Adjusting for optical effect................................................................................................................................................................................Done.
## Computing affinities.Done.
## Adjusting for non-specific binding................................................................................................................................................................................Done.
## Normalizing
## Calculating Expression
```

```
samples = sub("_.+","", sampleNames(gset))
sampleNames(gset) = samples
```

Now merge probes to genes by the means of all probes mapping to a particular entrez id

```
tab <- select(hgu133plus2.db, keys = keys(hgu133plus2.db), columns = c("ENTREZID"))
```

```
## Warning: 'select' resulted in 1:many mapping between keys and return rows
```

```
e <- exprs(gset)
geneExpr <- t(sapply(split(tab[,1], tab[,2]), function(ids){
                    colMeans(e[ids,,drop=FALSE])
                }))
rm(tab,e)
```

### 2. Load mutation and clinical data

Load clinical data for 159 MDS patients and 17 normals from Supplementary Table S1.

```
mdsData <- read.table("suppData/SuppTableS1GEO.txt", sep="\t", header=TRUE, check.names=FALSE) ## A tab-delimited version of Supplementary Table S1
head(mdsData)
```

```
##      PDID  GEPID      GEOID                  File Type Gender Age WHO_category Survival_days Status
## 1 PD6175a  GER20 GSM1420393 GSM1420393_MDS009.CEL  MDS      1  76           RA          2575      0
## 2 PD6173a  GER24 GSM1420394 GSM1420394_MDS011.CEL  MDS      1  61           RA           674      0
## 3 PD6185a  GER25 GSM1420395 GSM1420395_MDS012.CEL  MDS      1  50         RAEB          2514      0
## 4 PD6184a   GER4 GSM1420396 GSM1420396_MDS014.CEL  MDS      1  42         RAEB          2550      0
## 5 PD6183a   GER5 GSM1420397 GSM1420397_MDS015.CEL  MDS      1  51         RAEB          2549      0
## 6 PD6198a MDS135 GSM1420398 GSM1420398_MDS025.CEL  MDS      0  61      RCMD-RS          1057      1
##   AML_progression_days AML_status                                                 Karyotype Cytogenetic_risk  IPSS WPSS
## 1                   NA         NA 46, XX, t(1;3)(p33:p14), del(5)(q14;q34)[21] / 46, XX [4]                1 int-1 <NA>
## 2                   NA         NA               46, XX, del(5)(q14;q34) [21], inv9(q11;q12)                1 int-1 <NA>
## 3                   NA         NA                                   46, XX, del(5)(q14;q34)                0 int-1 <NA>
## 4                   NA         NA                                   46, XX, del(5)(q14;q34)                0 int-1 <NA>
## 5                   NA         NA                 46, XX, del(5)(q14;q34) [20] / 46, XX [5]                0 int-1 <NA>
## 6                   NA          0                                                    46, XY                0   low <NA>
##   Transfusion_dep Serum_ferritin PB_cytopenia Haemoglobin Absoulte_neutrophile_count Platelet_count BM_blasts_pct
## 1              NA             NA           NA         9.4                        1.2            331            NA
## 2              NA             NA           NA        10.7                        2.4            169            NA
## 3              NA             NA           NA        10.9                        1.3            445            NA
## 4              NA             NA           NA         6.4                        7.4           1042            NA
## 5              NA             NA           NA         9.0                        2.1            349            NA
## 6               1             NA           NA         7.9                        4.4            244            NA
##   ME_ratio Ring_sideroblasts_pct SF3B1 TET2 SRSF2 ASXL1 DNMT3A RUNX1 U2AF1 TP53 EZH2 IDH2 STAG2 ZRSR2 CBL BCOR NRAS
## 1       NA                  <NA>     0    0     0     0      0     0     0    0    0    0     0     0   0    0    0
## 2       NA                  <NA>     0    0     0     0      0     0     1    0    0    0     0     0   0    0    0
## 3       NA                  <NA>     0    0     0     0      0     0     0    0    0    0     0     0   0    0    0
## 4       NA                  <NA>     0    0     0     0      0     0     0    0    0    0     0     0   0    0    0
## 5       NA                  <NA>     0    0     0     1      0     0     0    0    0    0     0     0   0    0    0
## 6       NA                  <NA>     0    0     0     0      0     0     0    0    0    0     0     0   0    0    0
##   JAK2 CUX1 IDH1 KRAS PHF6 EP300 GATA2 NPM1 MLL2 PTPN11 CREBBP KIT MPL NF1 WT1 IRF1 RAD21 ATRX CDKN2A ETV6 KDM6A CEBPA
## 1    0    0    0    0    0     0     0    0    0      0      0   0   0   0   0    0     0    0      0    0     1     0
## 2    0    0    0    0    0     0     0    0    0      0      0   0   0   0   0    0     0    0      0    0     0     0
## 3    0    0    0    0    0     0     0    0    0      0      0   0   0   0   0    0     0    0      0    0     0     0
## 4    1    0    0    0    0     0     0    0    0      0      0   0   0   0   0    0     0    0      0    0     0     0
## 5    0    0    0    0    0     0     0    0    0      0      0   0   0   0   0    0     0    0      0    0     0     0
## 6    0    0    0    0    0     0     0    0    0      0      0   0   0   0   0    0     0    0      0    0     0     0
##   FLT3 GNAS PTEN SH2B3 BRAF CTNNA1 rearr.3q del(5q) -7/del(7q) tri(8) del(11q) del(12p) abn.17 tri(19) del(20q) del(Y)
## 1    0    0    0     0    0      0        1       1          0      0        0        0      0       0        0      0
## 2    0    0    0     0    0      0        0       1          0      0        0        0      0       0        0      0
## 3    0    0    0     0    0      0        0       1          0      0        0        0      0       0        0      0
## 4    0    0    0     0    0      0        0       1          0      0        0        0      0       0        0      0
## 5    1    0    0     0    0      0        0       1          0      0        0        0      0       0        0      0
## 6    0    0    0     0    0      0        0       0          0      0        0        0      0       0        0      0
##   other complex   date pb_cytopenia   hb anc_log plt_log bm_blasts_logit ring_sideroblasts_logit ipss age_imp
## 1     0       0 1.1627           NA  9.4  0.1832   5.802              NA                      NA    2      76
## 2     0       0 1.2326           NA 10.7  0.8759   5.130              NA                      NA    2      61
## 3     0       0 0.7091           NA 10.9  0.2631   6.098              NA                      NA    2      50
## 4     0       0 1.0988           NA  6.4  2.0016   6.949              NA                      NA    2      42
## 5     0       0 1.0408           NA  9.0  0.7424   5.855              NA                      NA    2      51
## 6     0       0 1.2460           NA  7.9  1.4818   5.497              NA                      NA    1      61
```

```
ix <- setdiff(na.omit(match(samples, mdsData$GEOID)), which(is.na(mdsData$PDID))) ## All MDS samples with expression and seq data
normalSamples <- as.character(mdsData$GEOID[mdsData$Type=="Normal"])
```

Define mappings

```
GEO2PD <- as.character(mdsData$PDID) 
names(GEO2PD) <- mdsData$GEOID
PD2GEO <- as.character(mdsData$GEOID)
names(PD2GEO) <- mdsData$PDID
```

#### Clinical summary statistics

```
f <- function(x) cat(paste(median(x, na.rm=TRUE), " median; ", min(x, na.rm=TRUE),"-",max(x, na.rm=TRUE), " range; ", sum(is.na(x)), " missing", sep=""),"\n")
mdsIx <- !is.na(mdsData$GEOID[mdsData$Type=="MDS"]) ## All MDS samples with expression data
table(mdsData$Gender[mdsIx])
```

```
## 
##   0   1 
## 102  57
```

```
f(mdsData$Age[mdsIx])
```

```
## 67 median; 19-87 range; 22 missing
```

```
table(mdsData$WHO_category[mdsIx])
```

```
## 
##     5q- AML-MDS    CMML      RA    RAEB  RAEB 1  RAEB 2   RAEB1   RAEB2    RARS  RARS-T    RCMD RCMD-RS 
##       6       7       7      13      14      13      23       1       5      14       6      27      22
```

```
f(as.numeric(as.character(mdsData$BM_blasts_pct[mdsIx])))
```

```
## 4 median; 0-63 range; 34 missing
```

```
f(as.numeric(as.character(mdsData$Ring_sideroblasts_pct[mdsIx])))
```

```
## 0.5 median; 0-94 range; 50 missing
```

```
f(mdsData$Haemoglobin[mdsIx])
```

```
## 9.5 median; 4.5-14.6 range; 32 missing
```

```
f(mdsData$Absoulte_neutrophile_count[mdsIx])
```

```
## 1.848 median; 0.08-920 range; 37 missing
```

```
f(mdsData$Platelet_count[mdsIx])
```

```
## 154 median; 10-45000 range; 25 missing
```

```
table(mdsData$PB_cytopenia[mdsIx])
```

```
## 
##  0  1 
## 52 72
```

### 3. Match expression and clinical data

Incrementally construct the design matrix

```
design = cbind(offset=1,mdsData[ix, grep("SF3B1|TET2|SRSF2|ASXL1|DNMT3A|RUNX1|U2AF1|TP53|EZH2|IDH2|STAG2|ZRSR2|CBL|BCOR|NRAS|JAK2|CUX1|IDH1|KRAS|PHF6|EP300|GATA2|NPM1|MLL2|PTPN11|CREBBP|KIT|MPL|NF1|WT1|IRF1|RAD21|ATRX|CDKN2A|ETV6|KDM6A|CEBPA|FLT3|GNAS|PTEN|SH2B3|BRAF|CTNNA1", colnames(mdsData))]) # oncogenic mutations
minF=5 ## Minimal number of alterations
design = design[,colSums(design)>=minF]
rownames(design) <- mdsData$GEOID[ix]
```

Cytogenetics

```
cytoImputed <- mdsData[ix, grep("rearr|del|tri|abn|complex|other", colnames(mdsData), value=TRUE)[-12:-11]]
cytoImputed <- cytoImputed[,colSums(cytoImputed, na.rm=TRUE)>0]
design <- cbind(design,  cytoImputed[,colSums(cytoImputed, na.rm=TRUE)>=minF], Gender=mdsData[ix,"Gender"], Age=scale(mdsData[ix,"age_imp"], center=TRUE, scale=FALSE))
```

Include 17 normal samples

```
n <- nrow(design)
design <- rbind(design, matrix(0, nrow=17, ncol=ncol(design), dimnames = list(NULL,colnames(design))))
design <- cbind(design, Normal=c(rep(0,n), rep(1,17)))
design[,1] <- 1
design[n+1:17,"Age"] <- NA #mean(design[1:n,"Age"])
design[n+1:17,"Gender"] <- geneExpr["7503",normalSamples] > 5 ## XIST expression
rownames(design)[n+1:17] <- normalSamples
design <- design[,c(1:17,20,18:19)]
```

Impute missing by mean

```
design0 <- design
for(j in 1:ncol(design))
    design[is.na(design[,j]),j] <- mean(design[,j], na.rm=TRUE)

head(design)
```

```
##            offset SF3B1 TET2 SRSF2 ASXL1 DNMT3A RUNX1 U2AF1 TP53 EZH2 STAG2 ZRSR2 JAK2 del(5q) tri(8) del(12p) del(20q)
## GSM1420393      1     0    0     0     0      0     0     0    0    0     0     0    0       1      0        0        0
## GSM1420394      1     0    0     0     0      0     0     1    0    0     0     0    0       1      0        0        0
## GSM1420395      1     0    0     0     0      0     0     0    0    0     0     0    0       1      0        0        0
## GSM1420396      1     0    0     0     0      0     0     0    0    0     0     0    1       1      0        0        0
## GSM1420397      1     0    0     0     1      0     0     0    0    0     0     0    0       1      0        0        0
## GSM1420398      1     0    0     0     0      0     0     0    0    0     0     0    0       0      0        0        0
##            Normal Gender     Age
## GSM1420393      0      1  10.589
## GSM1420394      0      1  -4.411
## GSM1420395      0      1 -15.411
## GSM1420396      0      1 -23.411
## GSM1420397      0      1 -14.411
## GSM1420398      0      0  -4.411
```

Define colors

```
colMutations = c(brewer.pal(8,"Set1")[-6], rev(brewer.pal(8,"Dark2")), brewer.pal(7,"Set2"))[c(1:12,16:19,13:15)]
o <- order(apply(col2rgb(colMutations),2,rgb2hsv)[1,])
colMutations <- colMutations[rev(o)][(4*1:19 +15) %% 19 + 1]
names(colMutations) <- colnames(design)[-1]
```

Show a venn diagram with the overlap

```
par(bty="n", mgp = c(2,.33,0), mar=c(3,3,1,0)+.1, las=1, tcl=-.25)
grid.newpage()
pushViewport(viewport(w = .9, h = .9))
grid.draw(venn.diagram(list(Sequenced=mdsData$GEOID[!is.na(mdsData$PDID)], Expression = samples, Normal=normalSamples), filename=NULL, lty=1, 
                col=colMutations[1:3], fill=colMutations[1:3], alpha=0.05, euler.d=TRUE, fontfamily="Helvetica", cat.fontfamily="Helvetica", cat.fontface="italic", euler.diagram=TRUE))
```

## 2. Model fitting

We use the lmFit function from the `limma` package. This comes with a whole series of powerful and reliable tests.

```
glm = lmFit(geneExpr[,rownames(design)], design = design ) 
glm = eBayes(glm)
```

### 1. F-statistic for all but offset

Here we want to determine all genes which are associated with any covariate. This will be based on an F-statistic.
lmFit also tests wether the offset is different from zero (trivially true).

```
F.stat <- classifyTestsF(glm[,-1],fstat.only=TRUE) # remove offset
glm$F <- as.vector(F.stat)
df1 <- attr(F.stat,"df1")
df2 <- attr(F.stat,"df2")
if(df2[1] > 1e6){ # Work around bug in R 2.1
    glm$F.p.value <- pchisq(df1*glm$F,df1,lower.tail=FALSE)
}else
    glm$F.p.value <- pf(glm$F,df1,df2,lower.tail=FALSE)
```

#### Random model

Compare to a model where all values of the covariates are randomly permuted. If all model assumptions were correct, this wouldn't be needed.

```
set.seed(42)
rlm <- lmFit(geneExpr[,rownames(design)], apply(design, 2, sample))
rlm <- eBayes(rlm)
F.stat <- classifyTestsF(rlm[,-1],fstat.only=TRUE)
rlm$F <- as.vector(F.stat)
df1 <- attr(F.stat,"df1")
df2 <- attr(F.stat,"df2")
if(df2[1] > 1e6){ # Work around bug in R 2.1
    rlm$F.p.value <- pchisq(df1*rlm$F,df1,lower.tail=FALSE)
}else
    rlm$F.p.value <- pf(rlm$F,df1,df2,lower.tail=FALSE)
```

#### Explained variance by different categories

The F-statistic is directly related to the R2.

```
F.stat <- classifyTestsF(glm[,2:16],fstat.only=TRUE) ## All genetics & cytogenetics
df1 <- attr(F.stat,"df1")
df2 <- attr(F.stat,"df2")
F.p.value <- pchisq(df1*F.stat,df1,lower.tail=FALSE)

R.stat <- classifyTestsF(rlm[,2:16],fstat.only=TRUE) ## Random

Rall = 1 - 1/(1 + glm$F * (ncol(design)-1)/(nrow(design)-ncol(design)))
Rgenetics = 1 - 1/(1 + F.stat * 15/(nrow(design)-ncol(design)))
Pgenetics = 1 - 1/(1 + R.stat * 15/(nrow(design)-ncol(design)))
names(Rgenetics) <- names(Pgenetics) <- names(Rall) <-  rownames(geneExpr)
```

Plot the variance explained by genetics

```
par(bty="n", mgp = c(2,.33,0), mar=c(3,2.5,1,1)+.1, las=1, tcl=-.25, xpd=NA)
d <- density(Pgenetics,bw=1e-3)
f <- 1#nrow(gexpr)/512
plot(d$x, d$y * f, col='grey', xlab=expression(paste("Explained variance per gene ", R^2)), main="", lwd=2, type="l", ylab="", xlim=c(0,0.7))
title(ylab="Density", line=1.5)
d <- density(Rgenetics, bw=1e-3)
r <- min(Rgenetics[p.adjust(F.p.value,"BH")<0.05])
x0 <- which(d$x>r)
polygon(d$x[c(x0[1],x0)], c(0,d$y[x0])* f, col=paste(set1[1],"44",sep=""), border=NA)
lines(d$x, d$y* f, col=set1[1], lwd=2)
#points(d$x[x0[1]], d$y[x0[1]]*f, col=set1[1], pch=16)
text(d$x[x0[1]], d$y[x0[1]]*f, pos=4, paste(sum(Rgenetics > r), "genes q < 0.05"))
arrows(Rgenetics["22"], par("usr")[4]/7, Rgenetics["22"], par("usr")[4]/50, length=0.05)
text(Rgenetics["22"], par("usr")[4]/8, "ABCB7", font=3, pos=3)
legend("topright", bty="n", col=c(set1[1], "grey"), lty=1, c("Observed","Random"), lwd=2)
```

#### Predictions

```
glmPrediction <- glm$coefficients %*% t(design)
rlmPrediction <- rlm$coefficients %*% t(design)
```

#### ABCB7 expression

```
par(bty="n", mgp = c(1.5,.33,0), mar=c(2.5,2.5,1,1)+.1, las=1, tcl=-.25)
plot(glmPrediction["22",], geneExpr["22",rownames(design)], ylab=expression(paste("Observed ",italic("ABCB7"), " expression")), xlab=expression(paste("Predicted ",italic("ABCB7"), " expression")), pch=16, cex=.8)
abline(0,1)
u <- par("usr")
par(xpd=NA)
y <- glm$coefficients["22",-1]+glm$coefficients["22",1]
u <- par("usr")
x0 <- rep(u[3]+1,ncol(design)-1)
y0 <- u[4] + 0.05*(u[4]-u[3]) - rank(-y)/length(y) * (u[4]-u[3])/1.2
d <- density(y)
lines(d$x, d$y/5+1+u[3], col="grey")
lines(d$x, -d$y/5+1+u[3], col="grey")
points(x=y, y=x0+violinJitter(y, magnitude=0.25)$y, col=colMutations, pch=16)
text(x=glm$coefficients["22",1], y= 1.5 +u[3], "Model coefficients", cex=0.8)
w <- glm$p.value["22",-1] < 0.01
rotatedLabel(y[w], x0[w]+0.1, labels=colnames(design)[-1][w], font=ifelse(grepl("[[:lower:]]", colnames(design)[-1]),1,3)[w], cex=.66, pos=1, col=colMutations[w])
axis(at=-1:1 + glm$coefficients["22",1], labels=-1:1, side=1, cex.axis=.8, line=-1, mgp = c(1.5,.05,0), tcl=-.15)
#mtext(at=l$coefficients[1], line=-2, side=1, "Coefficients", cex=.8)
text(u[1],u[4], substitute(paste(R^2==r),list(r=round(Rgenetics["22"],2))), pos=4)
```

```
#text(u[1],u[4]-(u[4]-u[3])*0.1, substitute(paste(R["Genetics"]^2==r),list(r=round(Rgenetics["22"],2))), pos=4)
```

### 2. Significant effects per covariate

Prepare the test results using a hierarchical procedure:
1. Adjust down transcripts using F-stat
2. Adjust along covariates

```
testResults <- decideTests(glm, method="hierarchical",adjust.method="BH", p.value=0.05)[,-1]
significantGenes <- sapply(1:ncol(testResults), function(j){
            c <- glm$coefficients[testResults[,j]!=0,j+1]
            table(cut(c, breaks=c(-5,seq(-1.5,1.5,l=7),5)))
        })
colnames(significantGenes) <- colnames(testResults)
```

#### Barplot

```
par(bty="n", mgp = c(2.5,.33,0), mar=c(3,3.3,2,0)+.1, las=2, tcl=-.25)
b <- barplot(significantGenes, las=2, ylab = "Differentially expressed genes", col=brewer.pal(8,"RdYlBu"), legend.text=FALSE , border=0, xaxt="n")#, col = set1[simple.annot[names(n)]], border=NA)
rotatedLabel(x0=b, y0=rep(10, ncol(significantGenes)), labels=colnames(significantGenes), cex=.7, srt=45, font=ifelse(grepl("[[:lower:]]", colnames(design))[-1], 1,3), col=colMutations)
clip(0,30,0,1000)
#text(b+0.2, colSums(n)+50, colSums(n), pos=3, cex=.7, srt=90)
x0 <- 21.5
image(x=x0+c(0,0.8), y=par("usr")[4]+seq(-100,100,l=9), z=matrix(1:8, ncol=8), col=brewer.pal(8,"RdYlBu"), add=TRUE)
text(x=x0+1.5, y=par("usr")[4]+seq(-50,50,l=3), format(seq(-1,1,l=3),2), cex=0.66)
lines(x=rep(x0+.8,2), y=par("usr")[4]+c(-75,75))
segments(x0+.8,par("usr")[4]+seq(-75,75,l=7),x0+.9,par("usr")[4]+seq(-75,75,l=7))
text(x0+.8, par("usr")[4]+125, "log2 FC", cex=.66)
rotatedLabel(b-0.1, colSums(significantGenes), colSums(significantGenes), pos=3, cex=, srt=45)
```

Associated mutations per transcript:

```
par(bty="n", mgp = c(2.5,.33,0), mar=c(3,3.3,3,0)+.1, las=1, tcl=-.25)
t <- table(rowSums(abs(testResults[,1:16])))
b <- barplot(t[-1],ylab="Differentially expressed genes", col=rev(brewer.pal(7, "Spectral")[-(4:5)]), border=NA)
rotatedLabel(b-0.1, t[-1], t[-1], pos=3, cex=1, srt=45)
title(xlab="Associated drivers", line=2)
```

#### Venn diagrams for significant effects

```
v <- apply(testResults!=0,2, which)
par(bty="n", mgp = c(2,.33,0), mar=c(3,3,1,0)+.1, las=1, tcl=-.25)
grid.newpage()
pushViewport(viewport(w = .9, h = .9))
w <- c("SF3B1","SRSF2","U2AF1","ZRSR2")
grid.draw(venn.diagram(v[w], filename=NULL, lty=1, col=colMutations[w][c(1,3,4,2)], fill=colMutations[w], alpha=0.05, euler.d=TRUE, fontfamily="Helvetica", cat.fontfamily="Helvetica", cat.fontface="italic"))
```

```
grid.newpage()
pushViewport(viewport(w = .9, h = .9))
w <- names(sort(colSums(testResults!=0), decreasing = TRUE))[1:4]
grid.draw(venn.diagram(v[w], filename=NULL, lty=1, col=colMutations[w], fill=colMutations[w], alpha=0.05, euler.d=TRUE, fontfamily="Helvetica", cat.fontfamily="Helvetica", cat.fontface="italic"))
```

#### Chromosomal distribution

```
chr = factor(sapply(AnnotationDbi::mget(rownames(geneExpr), org.Hs.egCHR, ifnotfound=NA), `[`,1), levels=c(1:22, "X","Y","MT"))
chromTable <- apply(testResults,2, function(x) table(chr[x!=0]))
```

```
par(bty="n", mgp = c(0.5,0.5,0), las=1, tcl=-.25, font.main=3, mfrow=c(4,5), xpd=NA, mar=c(0,0,1.5,0))
for(j in 1:ncol(testResults)){
    n <- sum(testResults[,j]!=0)
    pie(chromTable[,j], col=colorRampPalette(brewer.pal(11,'Spectral'))(24), border="white",  radius=0.8, init.angle=90, labels=ifelse(chromTable[,j]/sum(chromTable[,j]) > 0.02, paste("",rownames(chromTable), "(" ,chromTable[,j], ")",sep=""),""))
    title(main = colnames(chromTable)[j], font.main = ifelse(grepl("[[:lower:]]", colnames(chromTable)[j]), 1,3), cex.main=1.33)
    symbols(0,0,circles=.3, inches=FALSE, col="white", bg="white", lty=0, add=TRUE)
    #symbols(0,0,circles=.8*(1-sqrt(n/max(colSums(testResults!=0)))), col="white", add=TRUE, lty=0, bg="white", inches=FALSE)
    #cat(n,"\n")
}
t <- table(chr)
pie(t, col=colorRampPalette(brewer.pal(11,'Spectral'))(24), border="white",  radius=0.8, cex.main=1.33, labels=ifelse(t/sum(t) > 0.02, names(t),""), init.angle=90)
symbols(0,0,circles=.3, inches=FALSE, col="white", bg="white", lty=0, add=TRUE)
title(main = "# Genes", font.main = ifelse(grepl("[[:lower:]]", colnames(chromTable)[j]), 1,3))
```

### 3. Heatmaps for Figure 1b

```
par(bty="n", mgp = c(2,.33,0), mar=rep(0,4), las=1, tcl=-.25, xpd=NA)
plot(NA,NA, xlim=c(0,ncol(design)-1), ylim=c(0,nrow(design)), xaxt="n", yaxt="n", xlab="",ylab="", xaxs="i", yaxs="i")
z <- design[,-1]
h <- hclust(dist(z[,1:16]))
j <- hclust(dist(t(z)))
rasterImage(sapply(1:ncol(z), function(i) ifelse(z[,i]>0, colMutations[i-1], "#FFFFFF"))[h$order,j$order], 0, 0, ncol(design)-1, nrow(design), interpolate=FALSE)
```

```
par(bty="n", mgp = c(2,.33,0), mar=rep(0,4), las=1, tcl=-.25, xpd=NA)
w <- names(sort(Rgenetics, decreasing = TRUE)[1:1000])
z <- geneExpr[w,]-rowMeans(geneExpr[w,])
h <- hclust(dist(z))
i <- hclust(dist(t(z)))
plot(NA,NA, xlim=c(0,ncol(geneExpr)), ylim=c(0,1000), xaxt="n", yaxt="n", xlab="",ylab="", xaxs="i", yaxs="i")
rasterImage(matrix(brewer.pal(11,"RdBu")[cut(z[h$order,i$order], 12)], ncol=ncol(geneExpr)), 0,0,ncol(geneExpr),1000, interpolate=FALSE)
```

```
par(bty="n", mgp = c(2,.33,0), mar=rep(0,4), las=1, tcl=-.25, xpd=NA)
plot(NA,NA, xlim=c(0,ncol(design)-1), ylim=c(0,1000), xaxt="n", yaxt="n", xlab="",ylab="", xaxs="i", yaxs="i")
rasterImage(matrix(brewer.pal(11,"RdBu")[cut(glm$coefficients[w,-1][h$order,j$order], seq(-3,3,l=12))], ncol=ncol(design)-1), 0,0,ncol(design)-1,1000, interpolate=FALSE)
```

### 4. Test for gene and cytogenetic interactions

Here we want to compare how mutational co-occurrence and the sets of transcriptional changes are interlinked

```
genomicData = mdsData[,colnames(design)[2:17]]
interactions <- interactionsGenes <- sapply(1:ncol(genomicData), function(i) sapply(1:ncol(genomicData), function(j) {f<- try(fisher.test(genomicData[,i], genomicData[,j]), silent=TRUE); if(class(f)=="try-error") 0 else ifelse(f$estimate>1, -log10(f$p.val),log10(f$p.val))} ))
oddsRatio <- oddsGenes <- sapply(1:ncol(genomicData), function(i) sapply(1:ncol(genomicData), function(j) {f<- try(fisher.test(genomicData[,i] + .5, genomicData[,j] +.5), silent=TRUE); if(class(f)=="try-error") f=NA else f$estimate} ))
w <- p.adjust(glm$F.p.value,"BH")<0.05
oddsExpression <- sapply(1:ncol(genomicData), function(i) sapply(1:ncol(genomicData), function(j) {f<- try(fisher.test(abs(testResults[w,i]), abs(testResults[w,j])), silent=TRUE); if(class(f)=="try-error") f=NA else f$estimate} ))
interactionsExpression <- sapply(1:ncol(genomicData), function(i) sapply(1:ncol(genomicData), function(j) {f<- try(fisher.test(abs(testResults[w,i]), abs(testResults[w,j])), silent=TRUE); if(class(f)=="try-error") 0 else ifelse(f$estimate>1, -log10(f$p.val),log10(f$p.val))} ))
oddsRatio[lower.tri(oddsRatio)] <- oddsExpression[lower.tri(oddsExpression)]
interactions[lower.tri(interactions)] <- interactionsExpression[lower.tri(interactions)]

diag(interactions) <- NA
diag(oddsRatio) <- NA
colnames(oddsRatio) <- rownames(oddsRatio) <- colnames(interactions) <- rownames(interactions) <- colnames(genomicData)
oddsRatio[10^-abs(interactions) > 0.05] = 1
oddsRatio[oddsRatio<1e-3] = 1e-4
oddsRatio[oddsRatio>1e3] = 1e4
logOdds=log10(oddsRatio)

reorder <- function(M, o){
    u <- M
    u[lower.tri(u)] <- t(M)[lower.tri(M)]
    u <- u[o,o]
    l <- M
    l[upper.tri(u)] <- t(M)[upper.tri(M)]
    l <- l[o,o]
    R <- u
    R[lower.tri(R)] <- l[lower.tri(R)]
    return(R)
}
```

Plot (*Note: This plot looks a little different as in the paper, where mutation data for all 738 patients is used on the upper panel*).

```
par(bty="n", mgp = c(2,.5,0), mar=rep(4,4)+.1, las=2, tcl=-.33)
m <- nrow(oddsRatio)
n <- ncol(oddsRatio)
o = c(1,11,7,3,4,9,6,10,2,5,8,12:m)#h$order#c(h$order,(length(h$order) +1):ncol(interactions))
r <- reorder(log10(oddsRatio),o)
r[lower.tri(r)] <- NA
image(x=1:n, y=1:m, r, col=brewer.pal(9,"PiYG"), breaks = c(-4:0-.Machine$double.eps,0:4), xaxt="n", yaxt="n", xlab="",ylab="", xlim=c(0, n+4), ylim=c(0, n+4))
r <- reorder(log10(oddsRatio),o)
r[upper.tri(r)] <- NA
image(x=1:n, y=1:m, r, col=brewer.pal(9,"RdBu"), breaks = c(-4:0-.Machine$double.eps,0:4), add=TRUE)
mtext(side=2, at=1:n, colnames(oddsRatio)[o], font=ifelse(grepl('[[:lower:]]',colnames(oddsRatio)[o]),1,3), col=colMutations[1:16][o])
rotatedLabel(x0=1:n, y0=rep(0.5, n), colnames(oddsRatio)[o], font=ifelse(grepl('[[:lower:]]',colnames(oddsRatio)[o]),1,3), srt=45, cex=.9, col=colMutations[1:16][o])
abline(h = length(h$order)+.5, col="white", lwd=1)
abline(v = length(h$order)+.5, col="white", lwd=1)
abline(h=0:n+.5, col="white", lwd=.5)
abline(v=0:n+.5, col="white", lwd=.5)
text(x=n/2, y=m+.5, "Genetic interactions", pos=3)
text(x=n+1, y=m/2, "Overlap of expression targets", pos=3, srt=270)
q <- p.adjust(10^-abs(reorder(interactions,o)), method="BH")
p <- p.adjust(10^-abs(reorder(interactions,o)), method="holm")
w = arrayInd(which(q < .1), rep(m,2))
points(w, pch=".", col="white", cex=1.5)
w = arrayInd(which(p < .05), rep(m,2))
points(w, pch="*", col="white")
image(y = 1:8 +6, x=rep(n,2)+c(2,2.5)+1, z=matrix(c(1:8), nrow=1), col=brewer.pal(8,"PiYG"), add=TRUE)
image(y = 1:8 +6, x=rep(n,2)+c(2.5,3)+1, z=matrix(c(1:8), nrow=1), col=brewer.pal(8,"RdBu"), add=TRUE)
axis(side = 4, at = seq(1,7) + 6.5,  tcl=-.15, label=10^seq(-3,3), las=1, lwd=.5)
mtext(side=4, at=10, "Odds ratio", las=3, line=3)
par(xpd=NA)
text(x=n+2.2, y=15, "Correlated", pos=4)
text(x=n+2.2, y=6-.2, "Exclusive", pos=4)
points(x=rep(n,2)+3.5, y=1:2, pch=c("*","."))
image(x=rep(n,2)+c(2,3)+1, y=(3:4) -0.5, z=matrix(1), col=brewer.pal(3,"BrBG"), add=TRUE)
mtext(side=4, at=1:3, c("Not sig.", "Q < 0.1", "P < 0.05"), line=0.2)
```

### 5. Write output

```
t= topTable(glm, number=Inf)
annot <- select(org.Hs.eg.db, rownames(t), c("SYMBOL","GENENAME","CHR","CHRLOC"))
```

```
## Warning: 'select' resulted in 1:many mapping between keys and return rows
```

```
t <- cbind(annot[!duplicated(annot$ENTREZID),1:5],signif(cbind(t, R2.genetics=Rgenetics[rownames(t)], P.R2.genetics=F.p.value[rownames(t)]), 3), P = signif(glm$p.value[rownames(t),],2),Test=testResults[rownames(t),])
write.table(t[order(-t$R2.genetics),], file=paste(Sys.Date(),"-SuppTable2.txt",sep=""), sep="\t", row.names = FALSE, quote=FALSE)
```

### Contrast against normals

```
normalContrast <- rbind(rep(0,18), diag(1,18), rep(-1, 18))[c(1:17,20,18:19),]
colnames(normalContrast) <- setdiff(colnames(design)[2:20],"Normal")
normalContrast <- contrasts.fit(glm, normalContrast)
normalContrast <- eBayes(normalContrast)
```

### Expression of mutated genes

The next part will analyse how mutated genes themselves are expressed

```
par(bty="n", mgp = c(2.5,0.5,0), las=1, tcl=-.25, font.main=3)
layout(matrix(1:16, ncol=4, byrow = TRUE), heights=c(1,1,1,0.2))
w <- grep("[[:lower:]]", colnames(design), invert = TRUE)
p <- sapply(colnames(design)[w], function(i){
            geneId <- AnnotationDbi::get(i, revmap(org.Hs.egSYMBOL))

            c(  p = glm$p.value[geneId,c("Normal",i)],
            q = normalContrast$p.value[geneId,i])
        })
p[] <-  sig2star(p.adjust(p, "BH"), breaks=10^c(-.Machine$double.max.exp,-3:0),labels=c("***","**","*",""))
for(i in colnames(design)[w]){
    o <- colSums(p!="")[i]*.5 + (colSums(p!="")[i]>0)*.5
    par(mar=c(1.2,2.7, o +2,0.5)+.1)
    x <- design[,i]+1 -design[,"Normal"]
    geneId <- AnnotationDbi::get(i, revmap(org.Hs.egSYMBOL))
    v <- do.call("rbind", lapply(split(geneExpr[geneId, rownames(design)], x), violinJitter, magnitude=0.5))
    plot( sort(x) + v$y, v$x, ylab="", xaxt="n", xlab="", 
            col=paste(c(colMutations[c('Normal',i)],"#AAAAAA"),"DD", sep="")[c(1,3,2)][sort(x)+1], 
            xlim=c(-0.5,2.5), cex.main=1, pch = 16, cex=0.5, lwd=0)#genotypes[match(rownames(design), rownames(genotypes)),i]+1)
    title(ylab="Expression", line=2)
    title(main=i, line=o +.5)
    par(xpd=NA)
    boxplot(at=0:2, geneExpr[geneId, rownames(design)] ~ factor(design[,i] - design[,"Normal"]), add=TRUE, xaxt="n", boxwex=0.5, outline=FALSE, lty=1, outwex=0, staplewex=0, border=c(colMutations[c('Normal',i)],"#777777")[c(1,3,2)])
    l=0
    a = list(0:1,1:2, c(0,2))
    for(j in 3:1){
        if(p[j,i] != ""){
        axis(3, at=a[[j]], labels=NA, tcl=0.25, line=l+.5)
        mtext(side=3, at=a[[j]][1]+.5, p[j,i], line = l)
        l <- l+.5
    }
    }
    u <- par("usr")
    rotatedLabel(0:2, rep((u[3]+u[4])/2 - 0.4 * (u[4]-u[3])), paste(table(x),c("Normal", "wt", "mut")))
    y <- glm$coefficients[AnnotationDbi::get(i, revmap(org.Hs.egSYMBOL)),c("offset",i, "Normal")]
    lines(0:2, y[1] + c(y[3],0,y[2]))#, pch=21, bg="white",col=c(colMutations[c('normal',i)],"#AAAAAA")[c(1,3,2)], type="b", cex=1.25)
}
```

Volcano plot

```
par(bty="n", mgp = c(2,.33,0), mar=c(3,3,1,1)+.1,  tcl=-.25)
set.seed(42)
s <- sample(1:nrow(glm),500)
plot(glm$coefficients[s,-1], (1/glm$p.value[s,-1]), log="y", xlab="Expression logFC", ylab="1/P-value", col="grey", pch=16)
x <- sapply(colnames(design)[w], function(i) glm$coefficients[AnnotationDbi::get(i, revmap(org.Hs.egSYMBOL)),i])
y <-  (1/sapply(colnames(design)[w], function(i) glm$p.value[AnnotationDbi::get(i, revmap(org.Hs.egSYMBOL)),i]))
points(x, y, pch=16)
text(x,y,ifelse(y>1e2,names(x),""), pos=3, font=3)
```

```
par(bty="n", mgp = c(2.5,.5,0), mar=c(1,4,4,2)+.1,  tcl=-.25)
z <- glm$coefficients[unlist(AnnotationDbi::mget(colnames(design)[w], revmap(org.Hs.egSYMBOL))),-1]
n <- nrow(z)
m <- ncol(z)
image(x=1:n,y=1:m, z, col=colorRampPalette(brewer.pal(9,"RdBu"))(19), breaks=seq(-2,2,l=20), xaxt="n", yaxt="n", xlab="",ylab="", xlim=range(0:n)+c(0.5,2.5))
mtext(side=3, at=1:n, colnames(design)[w], las=2, font=3, line=0.2)
mtext(side=2, at=1:m, colnames(z), las=2, font=ifelse(grepl("[[:upper:]]", colnames(design))[-1], 3,1), line=0.2)
text(rep(1:n, m), rep(1:m,each=n), sig2star(p.adjust(glm$p.value[unlist(AnnotationDbi::mget(colnames(design)[w], revmap(org.Hs.egSYMBOL))),-1], method="BH")))
image(x=n+2 + c(-0.5,0.5), y=1:4, z=matrix(1:4, ncol=4), col=brewer.pal(5,"RdBu")[-5], add=TRUE)
mtext(side=4, at=1:4, text=-2:1, las=2, adj=1, line=1)
```

## 2. Principal components analysis

```
pca <- prcomp(t(geneExpr))
```

### 1. Explained variance by principal components

```
par(bty="n", mgp = c(2.5,.5,0), mar=c(3,4,1,2)+.1, tcl=-.25, las=1)
plot(pca$sdev^2/sum(pca$sdev^2), type="h", col=set1[1], xlab="", ylab=expression(paste("Explained variance ", Rgenetics^2)) , ylim=c(0,0.15), yaxs="i")
mtext(side=1, "Principal component", line=2)
c <- cumsum(pca$sdev^2)/sum(pca$sdev^2)* pca$sdev[1]^2/sum(pca$sdev^2)
lines(c  , type="s")
axis(4, at = pretty(c(0,1))* pca$sdev[1]^2/sum(pca$sdev^2), labels=pretty(c(0,1)))
legend("bottomright", col=c(set1[1],"black"), lty=1, c("Per PC","Cumulative"), bty="n")
lines(c(180,20,20),c(c[20],c[20],0), lty=3)
```

### 2. Spatial clustering

We test wether samples with a given mutations cluster in the PCA world

```
x = pca$x[rownames(design),1:20, drop=FALSE]
d = apply(design[,-1], 2, function(y) mean(sqrt(rowSums((x[y>0,,drop=FALSE] - rep(colMeans(x[y>0,]),each=sum(y>0)))^2)*rep(pca$sdev[1:20]^2/sum(pca$sdev[1:20]^2), each=sum(y>0)))))
d0 = apply(design[,-1], 2, function(y) sapply(1:1000, function(i) mean(sqrt(rowSums((x[sample(y)>0,,drop=FALSE] - rep(colMeans(x[sample(y)>0,]),each=sum(y>0)))^2)*rep(pca$sdev[1:20]^2/sum(pca$sdev[1:20]^2), each=sum(y>0))))))
```

```
par(bty="n", mgp = c(2,.33,0), mar=c(4,3,1,1)+.1, las=2, tcl=-.25, xpd=NA)
boxplot(d0, las=2, pch=16, cex=.5, staplewex=0, lty=1, border="grey", ylab="Average distance", xaxt="n", ylim=range(d0))
rotatedLabel(x0=1:length(d), y0 = par("usr")[3], names(d), font = ifelse(grepl("[[:upper:]]", names(d)), 3,1), cex=0.8, srt=45)
points(d, col=set1[1], pch=19, cex=1.3)
legend("topleft", bty="n", legend=c("Observed","Random"), pch=c(19,19), col=c(set1[1],"grey"), pt.cex=c(1,0.5))
p <-  colMeans(d0 < rep(d, each=1000)) + 1/1000
rotatedLabel(x0=1:length(d), y0 = par("usr")[3]-1.5, sig2star(p.adjust(p, method="BH")), cex=0.8, srt=45, pos=3)
```

### 3. PCA overview

Now plot a large panel with small multiples of the first two PCs overlaid with the mutation status of each gene.

```
par(bty="n", mgp = c(0,0.5,0), mar=c(1,1,1.5,0)+.1, las=1, tcl=-.25, font.main=3, mfrow=c(4,5), xpd=NA)
i<-0
for (geneId in colnames(design)[2:20]){
    i<-i+1
    plot(pca$x[rownames(design),], cex=0.5, 
            pch=NA, 
            xlab=ifelse(i==1,"PC1",""), ylab=ifelse(i==1,"PC2",""),main=geneId, font.main=ifelse(grepl("[[:lower:]]",geneId),1,3), cex.main=1.33, cex.lab=1.2,xaxt="n", yaxt="n", ylim=c(-55,55))
    if(geneId != "Age")
        w <- rownames(design)[design[,geneId] == 1]
    else
        w <- rownames(design)[which(design0[,geneId] > median(design0[,geneId], na.rm=TRUE))]
    points(pca$x[!rownames(pca$x) %in% w,], pch=ifelse(is.na(design0[!rownames(pca$x) %in% w,geneId]),1,19), cex=0.5, col="grey", lwd=0.5)
    points(pca$x[w,], pch=16, cex=0.85, col=colMutations[i], lwd=0.05)
    u <- matrix(par("usr"), ncol=2)
    if(i==1){
        arrows(u[1,1],u[1,2], u[2,1],u[1,2],length=0.02)
        arrows(u[1,1],u[1,2], u[1,1],u[2,2],length=0.02)
    }
    text(u[2],u[4]*.8, labels=paste("n=",length(w), sep=""), bty="n", cex=1.2, pos=2)
}
plot.new()
legend("center",c("Missing","Wildtype","Mutant","Normal","Female","Age>median"), pch=c(1,19,19,19,19,19), col=c("grey","grey","black", colMutations[c("Normal","Gender","Age")]), bty="n", pt.cex=c(0.5,0.5,rep(0.85,4)),cex=1.2, pt.lwd=0.5)
```

### 4. Write PCA output

```
annot <- select(org.Hs.eg.db, rownames(glm), c("SYMBOL","GENENAME","CHR","CHRLOC"))
```

```
## Warning: 'select' resulted in 1:many mapping between keys and return rows
```

```
t <- cbind(annot[!duplicated(annot$ENTREZID),1:5], signif(cbind(pca$rotation, 3)))
write.table(t[order(t[,"PC1"]),1:20], file=paste(Sys.Date(),"-PCA-rotation.txt",sep=""), sep="\t", row.names = FALSE, quote=FALSE)
```

### 5. GLM projection

We can project the predicted expression levels for each sample back to the first PCs

```
par(bty="n", mgp = c(2,.33,0), mar=c(2,2.5,1,1)+.1, las=1, tcl=-.25, xpd=NA)
glmProjection <-  t(glm$coefficients[,-1] ) %*% pca$rotation[,1:20]
for(i in seq(1,19,2)){
    plot(pca$x[,i + 0:1], col="grey", cex=0.25, pch=19, xlim=range(pca$x[,i])*0.8, ylim=range(pca$x[,i+1])*0.8, xlab="", ylab="")
    u <- matrix(par("usr"), ncol=2)
    r <- sqrt(rowSums(glmProjection[,i + 0:1]^2))
    w <- order(r, decreasing=TRUE)[1:6]
    arrows(0,0,glmProjection[w,i], glmProjection[w,i+1], col=colMutations[w], pch=19, length = 0.05)
    text(glmProjection[w,i+0:1] + (glmProjection[w,i+0:1]/r[w]) * rep(diff(u)*c(0.15,0.1)*1.33, each=6), col=colMutations[w], pch=19, rownames(glmProjection)[w], font=ifelse(grepl("[[:upper:]]",rownames(glmProjection)[w]), 3,1))
    w <- TRUE
    points(glmProjection[w,i+0:1] , col=paste(colMutations[w],"88", sep=""), pch=16, cex=sqrt(colSums(significantGenes)/100)+.5)
    mtext(side=1, at=u[2,1], paste("PC",i), line=1, font=2)
    mtext(side=2, at=u[2,2], paste("PC",i+1), line=.5, font=2)
}
```

### 6. Enrichment of GO Terms in PC1&2

Here we test for an enrichments of GO terms using t-statistics

```
k = AnnotationDbi::as.list(org.Hs.egGO2EG)
k = k[sapply(k, length)>=10]
c <- pca$rotation[,1:2] *  sqrt(glm$s2.post)
n <- rownames(pca$rotation)
t = mclapply(k, function(p){
            ids = n %in% p
            sapply(1:ncol(c), function(j){
                        if(sum(ids)>2){
                            t <- t.test(c[ids,j], c[!ids,j], alternative="two.sided")
                            v <- var.test(c[ids,j], c[!ids,j], alternative="greater")
                            c(t$p.value, -diff(t$estimate),
                                    v$p.value, v$estimate)
                        }else 
                            rep(NA,4)
                    })
        }, mc.cores=1)
GO.pca = matrix(unlist(t), byrow = TRUE, nrow=length(t), dimnames=list(names(t),as.vector(outer(c("pval.t","shift","pval.F","var.odds"),paste(colnames(c), sep=""), paste))))

Term(names(sort(GO.pca[p.adjust(GO.pca[,1],"BH")<0.1,2])[1:20]))
```

```
##                                GO:0005833                                GO:0005344 
##                      "hemoglobin complex"             "oxygen transporter activity" 
##                                GO:0050832                                GO:0019864 
##              "defense response to fungus"                             "IgG binding" 
##                                GO:0008009                                GO:0006956 
##                      "chemokine activity"                   "complement activation" 
##                                GO:0030593                                GO:0071294 
##                   "neutrophil chemotaxis"           "cellular response to zinc ion" 
##                                GO:0042742                                GO:0006935 
##           "defense response to bacterium"                              "chemotaxis" 
##                                GO:0090382                                GO:0006968 
##                    "phagosome maturation"               "cellular defense response" 
##                                GO:0003823                                GO:0006955 
##                         "antigen binding"                         "immune response" 
##                                GO:0006874                                GO:0031100 
##        "cellular calcium ion homeostasis"                      "organ regeneration" 
##                                GO:0006783                                GO:0006954 
##               "heme biosynthetic process"                   "inflammatory response" 
##                                GO:0043202                                GO:0071222 
##                         "lysosomal lumen" "cellular response to lipopolysaccharide"
```

```
Term(names(sort(GO.pca[p.adjust(GO.pca[,1],"BH")<0.1,2], decreasing = TRUE)[1:20]))
```

```
##                                                          GO:0000042 
##                                        "protein targeting to Golgi" 
##                                                          GO:0030878 
##                                         "thyroid gland development" 
##                                                          GO:0046854 
##                              "phosphatidylinositol phosphorylation" 
##                                                          GO:0048853 
##                                           "forebrain morphogenesis" 
##                                                          GO:0048704 
##                           "embryonic skeletal system morphogenesis" 
##                                                          GO:0004709 
##                                 "MAP kinase kinase kinase activity" 
##                                                          GO:0009953 
##                                  "dorsal/ventral pattern formation" 
##                                                          GO:0007409 
##                                                      "axonogenesis" 
##                                                          GO:0007276 
##                                                 "gamete generation" 
##                                                          GO:2000463 
## "positive regulation of excitatory postsynaptic membrane potential" 
##                                                          GO:0004535 
##                            "poly(A)-specific ribonuclease activity" 
##                                                          GO:0019083 
##                                               "viral transcription" 
##                                                          GO:0006415 
##                                         "translational termination" 
##                                                          GO:0005088 
##                    "Ras guanyl-nucleotide exchange factor activity" 
##                                                          GO:0009451 
##                                                  "RNA modification" 
##                                                          GO:0001736 
##                                  "establishment of planar polarity" 
##                                                          GO:0080182 
##                                      "histone H3-K4 trimethylation" 
##                                                          GO:0000979 
##     "RNA polymerase II core promoter sequence-specific DNA binding" 
##                                                          GO:0009952 
##                          "anterior/posterior pattern specification" 
##                                                          GO:0070528 
##                                "protein kinase C signaling cascade"
```

```
Term(names(sort(GO.pca[p.adjust(GO.pca[,5],"BH")<0.1,6])[1:20]))
```

```
##                                         GO:0008009                                         GO:0030593 
##                               "chemokine activity"                            "neutrophil chemotaxis" 
##                                         GO:0031663                                         GO:0006935 
##    "lipopolysaccharide-mediated signaling pathway"                                       "chemotaxis" 
##                                         GO:0050729                                         GO:0006954 
##     "positive regulation of inflammatory response"                            "inflammatory response" 
##                                         GO:0006968                                         GO:0060333 
##                        "cellular defense response"      "interferon-gamma-mediated signaling pathway" 
##                                         GO:0045071                                         GO:0071222 
##  "negative regulation of viral genome replication"          "cellular response to lipopolysaccharide" 
##                                         GO:0010875                                         GO:0006955 
##        "positive regulation of cholesterol efflux"                                  "immune response" 
##                                         GO:0050830                                         GO:0006928 
##      "defense response to Gram-positive bacterium"                      "cellular component movement" 
##                                         GO:0030316                                         GO:0042088 
##                       "osteoclast differentiation"                  "T-helper 1 type immune response" 
##                                         GO:0032695                                         GO:0032715 
## "negative regulation of interleukin-12 production"  "negative regulation of interleukin-6 production" 
##                                         GO:0090382                                         GO:0009615 
##                             "phagosome maturation"                                "response to virus"
```

```
Term(names(sort(GO.pca[p.adjust(GO.pca[,5],"BH")<0.1,6], decreasing=TRUE)[1:20]))
```

```
##                                                                 GO:0007080 
##                                      "mitotic metaphase plate congression" 
##                                                                 GO:0007051 
##                                                     "spindle organization" 
##                                                                 GO:0000940 
##                                   "condensed chromosome outer kinetochore" 
##                                                                 GO:0000070 
##                                     "mitotic sister chromatid segregation" 
##                                                                 GO:0000083 
## "regulation of transcription involved in G1/S phase of mitotic cell cycle" 
##                                                                 GO:0006270 
##                                               "DNA replication initiation" 
##                                                                 GO:0007076 
##                                          "mitotic chromosome condensation" 
##                                                                 GO:0042555 
##                                                              "MCM complex" 
##                                                                 GO:0006271 
##                        "DNA strand elongation involved in DNA replication" 
##                                                                 GO:0032201 
##                   "telomere maintenance via semi-conservative replication" 
##                                                                 GO:0000777 
##                                         "condensed chromosome kinetochore" 
##                                                                 GO:0009982 
##                                          "pseudouridine synthase activity" 
##                                                                 GO:0000722 
##                                   "telomere maintenance via recombination" 
##                                                                 GO:0045120 
##                                                               "pronucleus" 
##                                                                 GO:0034080 
##                      "CENP-A containing nucleosome assembly at centromere" 
##                                                                 GO:0000775 
##                                           "chromosome, centromeric region" 
##                                                                 GO:0006297 
##                              "nucleotide-excision repair, DNA gap filling" 
##                                                                 GO:0005876 
##                                                      "spindle microtubule" 
##                                                                 GO:0017056 
##                                   "structural constituent of nuclear pore" 
##                                                                 GO:0007094 
##                                      "mitotic spindle assembly checkpoint"
```

```
par(bty="n", mgp = c(2,.33,0), mar=c(3,3,1,1)+.1, tcl=-.25, xpd=NA)
for(i in 0:1 * 2)
    plot(GO.pca[GO.pca[,1+i]<0.05,2:1+i], log="y", cex=sqrt(sapply(k[GO.pca[,1+i]<0.05], length)/100), pch=19, col=paste(ifelse(p.adjust(GO.pca[,1+i],"BH")[GO.pca[,1+i]<0.05]<0.1,set1[3],set1[1]),"33", sep=""), lwd=0)
```

```
## Warning: 226 y values <= 0 omitted from logarithmic plot
```

```
write.table(data.frame(GO.id=rownames(GO.pca), 
                GO.Term = Term(names(k)) , 
                n.genes = sapply(k, length),
                GO.pca),
        file=paste(Sys.Date(),"-PCA-GO.txt", sep=""), sep="\t", row.names=FALSE, quote=FALSE)
```

```
## Loading required package: GO.db
```

### 7. PC rotations

The rotations of the PCs reveal the more influential genes in each component

```
x <- pca$rotation[,1] * sqrt(glm$s2.post)
y <- pca$rotation[,2] * sqrt(glm$s2.post)
```

```
par(bty="n", mgp = c(2,.33,0), mar=c(3,3,2,2)+.1, las=1, tcl=-.25, xpd=NA)
w <- sqrt(x^2 + y^2) > 0.01
plot(x[w],y[w], pch=19, lwd=0, col=paste(set1[2],"22", sep=""), xlab="PC1", ylab="PC2")
points(0,0,pch=19, col=set1[2], cex=8)
up1 <- names(sort(GO.pca[p.adjust(GO.pca[,1],"BH")<0.1,2], decreasing=TRUE)[1])
up2 <- names(sort(GO.pca[p.adjust(GO.pca[,5],"BH")<0.1,6], decreasing=TRUE)[1])
down1 <- names(sort(GO.pca[p.adjust(GO.pca[,1],"BH")<0.1,2])[1])
down2 <- names(sort(GO.pca[p.adjust(GO.pca[,5],"BH")<0.1,6])[1])
rug(x[rownames(pca$rotation) %in% org.Hs.egGO2EG[[down1]]], col=set1[1], lwd=1, side=3)
rug(x[rownames(pca$rotation) %in% AnnotationDbi::mget(up1, org.Hs.egGO2EG)], col=set1[3], lwd=1, side=3)
rug(y[rownames(pca$rotation) %in% org.Hs.egGO2EG[[down2]]], col=set1[1], side=4, lwd=1)
rug(y[rownames(pca$rotation) %in% org.Hs.egGO2EG[[up2]]], col=set1[3], side=4, lwd=1)
w <- rownames(pca$rotation) %in% unlist(AnnotationDbi::mget(c(down1,up1,down2,up2),org.Hs.egGO2EG))
text(x[w], y[w] ,  unlist(AnnotationDbi::mget(rownames(glm)[w], org.Hs.egSYMBOL)), font=3, cex=0.66)
mtext(side=3, at=mean(x[rownames(pca$rotation) %in% org.Hs.egGO2EG[[down1]]]), Term(down1), col=set1[1] , line=-1.5)
mtext(side=3, at=mean(x[rownames(pca$rotation) %in% org.Hs.egGO2EG[[up1]]]), Term(up1), col=set1[3] , line=0.2)
par(las=3)
mtext(side=4, at=mean(y[rownames(pca$rotation) %in% org.Hs.egGO2EG[[down2]]]), Term(down2), col=set1[1] , line=-1.5)
mtext(side=4, at=mean(y[rownames(pca$rotation) %in% org.Hs.egGO2EG[[up2]]]), Term(up2), col=set1[3] , line=0.2)
```

### 8. F and GO

Here we test wether any GO category is associated with higher F-statistics, which would indicate a stronger association of these gene sets with our mutations.

```
k = AnnotationDbi::as.list(org.Hs.egGO2EG)
k = k[sapply(k, length)>=10]
n <- rownames(pca$rotation)
c <- glm$F
t = sapply(k, function(p){
            ids = n %in% p
            if(sum(ids)>2){
                t <- wilcox.test(c[ids], c[!ids], alternative="greater")
                c(t$p.value, t$statistic/sum(ids)/sum(!ids))
            }else 
                rep(NA,2)
        })
GO.F = matrix(t(t), ncol=2, dimnames=list(colnames(t),as.vector(outer(c("pval.U","U"),paste(colnames(c), sep=""), paste))))
colnames(GO.F) <- c("pval.U","U")
sum(p.adjust(GO.F[,1],"BH")<0.1)
```

```
## [1] 349
```

```
Term(names(tail(sort(GO.F[p.adjust(GO.F[,1],"BH")<0.05,2]), 20)))
```

```
##                                                                 GO:0051537 
##                                         "2 iron, 2 sulfur cluster binding" 
##                                                                 GO:0048037 
##                                                         "cofactor binding" 
##                                                                 GO:0004697 
##                                                "protein kinase C activity" 
##                                                                 GO:0004114 
##                       "3',5'-cyclic-nucleotide phosphodiesterase activity" 
##                                                                 GO:0035371 
##                                                     "microtubule plus end" 
##                                                                 GO:0006783 
##                                                "heme biosynthetic process" 
##                                                                 GO:0071397 
##                                         "cellular response to cholesterol" 
##                                                                 GO:0005763 
##                                    "mitochondrial small ribosomal subunit" 
##                                                                 GO:0043220 
##                                               "Schmidt-Lanterman incisure" 
##                                                                 GO:0000075 
##                                                    "cell cycle checkpoint" 
##                                                                 GO:0000722 
##                                   "telomere maintenance via recombination" 
##                                                                 GO:0006271 
##                        "DNA strand elongation involved in DNA replication" 
##                                                                 GO:0005344 
##                                              "oxygen transporter activity" 
##                                                                 GO:0051571 
##                         "positive regulation of histone H3-K4 methylation" 
##                                                                 GO:0000083 
## "regulation of transcription involved in G1/S phase of mitotic cell cycle" 
##                                                                 GO:0032201 
##                   "telomere maintenance via semi-conservative replication" 
##                                                                 GO:0006297 
##                              "nucleotide-excision repair, DNA gap filling" 
##                                                                 GO:0007051 
##                                                     "spindle organization" 
##                                                                 GO:0006270 
##                                               "DNA replication initiation" 
##                                                                 GO:0005833 
##                                                       "hemoglobin complex"
```

```
Term(names(head(sort(GO.F[p.adjust(GO.F[,1],"BH")<0.05,1]), 20)))
```

```
##                         GO:0005515                         GO:0005737                         GO:0005634 
##                  "protein binding"                        "cytoplasm"                          "nucleus" 
##                         GO:0005829                         GO:0005739                         GO:0005654 
##                          "cytosol"                    "mitochondrion"                      "nucleoplasm" 
##                         GO:0000278                         GO:0005524                         GO:0010467 
##               "mitotic cell cycle"                      "ATP binding"                  "gene expression" 
##                         GO:0006260                         GO:0006281                         GO:0005813 
##                  "DNA replication"                       "DNA repair"                       "centrosome" 
##                         GO:0007067                         GO:0051301                         GO:0044281 
##                          "mitosis"                    "cell division" "small molecule metabolic process" 
##                         GO:0003723                         GO:0005759                         GO:0005743 
##                      "RNA binding"             "mitochondrial matrix"     "mitochondrial inner membrane" 
##                         GO:0005730                         GO:0006915 
##                        "nucleolus"                "apoptotic process"
```

```
write.table(data.frame(GO.id=rownames(GO.F), 
                GO.Term = Term(names(k)) , 
                n.genes = sapply(k, length),
                GO.F,
                adj.P.Val=p.adjust(GO.F[,1], "BH")),
        file=paste(Sys.Date(),"-GO-F.txt", sep=""), sep="\t", row.names=FALSE, quote=FALSE)
```

### 9. PCA stability

Here we assess the stability of the PCA components using subsamples

```
set.seed(42)
s <- seq(20, ncol(geneExpr), 20)
c <- sapply(s, function(i) {
            rowMeans(abs(sapply(1:20, function(b){
                        y <-  prcomp(t(geneExpr[,sample(1:ncol(geneExpr),i)]))$rotation[,1:20]; 
                        diag(cor(pca$rotation[,1:20],y))})))
})
plot(c(s,176), c(abs(c[1,]),1), type='l', col=set1[1], xlab="Samples", ylab="Abs. correlation with PC", ylim=c(0,1))
j <- 1
for(i in c(2,5,10,15,20)){
    j <- j+1
    lines(c(s,176), c(abs(c[i,]),1), type='l', col=set1[j])
}
legend("bottomright", c("PC1","PC2","PC5","PC10","PC15","PC20"), col= set1[1:6], lty=1, bty="n")
```

## 3. Functional annotatition

In this section we will analyse the ENCODE annotation of those genomic regions that change in expression.

### 1. Load chromHMM data

```
tmpf <- tempfile()
utils::download.file("http://hgdownload.cse.ucsc.edu/goldenpath/hg19/encodeDCC/wgEncodeBroadHmm/wgEncodeBroadHmmK562HMM.bed.gz", tmpf)
tmp <- read.table(tmpf, skip=1, sep="\t")
chromHMMK562 <- GRanges(sub("","",tmp$V1), IRanges(tmp$V2, tmp$V3), class=tmp$V4, col=factor(as.numeric(tmp$V9), labels=sapply(levels(tmp$V9), function(x) do.call("rgb", as.list(c(sapply(strsplit(x,","), as.numeric), maxColorValue = 255))))))
tmpf <- tempfile()
utils::download.file("http://hgdownload.cse.ucsc.edu/goldenpath/hg19/encodeDCC/wgEncodeBroadHmm/wgEncodeBroadHmmGm12878HMM.bed.gz", tmpf)
tmp <- read.table(tmpf, skip=1, sep="\t")
chromHMMGm12878 <- GRanges(sub("","",tmp$V1), IRanges(tmp$V2, tmp$V3), class=tmp$V4, col=factor(as.numeric(tmp$V9), labels=sapply(levels(tmp$V9), function(x) do.call("rgb", as.list(c(sapply(strsplit(x,","), as.numeric), maxColorValue = 255))))))

colChromHmm <- as.character(chromHMMK562$col[!duplicated(chromHMMK562$class)])
names(colChromHmm) <- (unique(chromHMMK562$class))
colChromHmm <- colChromHmm[order(as.numeric(sub("([0-9]+)_.+","\\1", names(colChromHmm))))]
colChromHmm[13] <- "#BBBBBB"
colChromHmm[14:15] <- "#DDDDDD"
```

### 2. Target genes as GRanges

```
targetGenes <- GRangesList(sapply(colnames(testResults), function(j){
                    tmp <- na.omit(AnnotationDbi::select(org.Hs.eg.db, names(which(testResults[,j]<0)), c("ENTREZID","CHR","CHRLOC", "CHRLOCEND")))
                    GRanges(paste("chr",tmp$CHR,sep=""), shift(IRanges(ifelse(tmp$CHRLOC >0 , tmp$CHRLOC, -tmp$CHRLOCEND), width=10000),-5000), entrezid=tmp$ENTREZID)
                }))
targetGenesUp <- GRangesList(sapply(colnames(testResults), function(j){
                    tmp <- na.omit(AnnotationDbi::select(org.Hs.eg.db, names(which(testResults[,j]>0)), c("ENTREZID","CHR","CHRLOC", "CHRLOCEND")))
                    GRanges(paste("chr",tmp$CHR,sep=""), IRanges(abs(tmp$CHRLOC), abs(tmp$CHRLOCEND)), strand=ifelse(tmp$CHRLOC>0,"+","-"), entrezid=tmp$ENTREZID)
                }))
targetGenesDown <- GRangesList(sapply(colnames(testResults), function(j){
                    tmp <- na.omit(AnnotationDbi::select(org.Hs.eg.db, names(which(testResults[,j]<0)), c("ENTREZID","CHR","CHRLOC", "CHRLOCEND")))
                    GRanges(paste("chr",tmp$CHR,sep=""), IRanges(abs(tmp$CHRLOC), abs(tmp$CHRLOCEND)), strand=ifelse(tmp$CHRLOC>0,"+","-"), entrezid=tmp$ENTREZID)
                }))

sapply(targetGenesUp, function(x){
            y <- subsetByOverlaps(chromHMMK562,reduce(x),type="within")
            sapply(split(y, y$class), function(z) sum(width(z)))
        }) -> chromHmmUp
sapply(targetGenesDown, function(x){
            y <- subsetByOverlaps(chromHMMK562,reduce(x),type="within")
            sapply(split(y, y$class), function(z) sum(width(z)))
        }) -> chromHmmDown

tmp <- na.omit(AnnotationDbi::select(org.Hs.eg.db, rownames(geneExpr), c("CHR","CHRLOC","CHRLOCEND","ENTREZID")))
tmp <- tmp[tmp$CHR != "Un",]
tss <- sort(GRanges(paste("chr",tmp$CHR,sep=""), shift(IRanges(ifelse(tmp$CHRLOC >0 , tmp$CHRLOC, -tmp$CHRLOCEND), width=1000),-500), entrezid=tmp$ENTREZID))

t0 <- table(chromHMMK562[chromHMMK562 %over% reduce(tss)]$class)
o <- order(as.numeric(sub("_.+","",names(t0))))
```

```
par(bty="n", mgp = c(1.7,.33,0), mar=c(2.5,2.7,1,0)+.1, las=2, tcl=-.25)
plot(c(0, max(b)+1),c(0,0),xlim=c(0.5,33), ylab="Megabases",las=2, ylim=c(-20,15),xlab="", xaxt="n", type="l")
b <- barplot(chromHmmUp[o,]/1e6, col=colChromHmm, border=0.1,names.arg=rep("", ncol(chromHmmUp)), add=TRUE, yaxt="n")
barplot(-chromHmmDown[o,]/1e6, col=colChromHmm,add=TRUE, names.arg=rep("", ncol(chromHmmUp)), yaxt="n", border=NA)
rotatedLabel(x0=b, y0=rep(-20, ncol(chromHmmUp)), labels=colnames(chromHmmDown), cex=.7, srt=45, font=ifelse(grepl("[[:lower:]]", colnames(design))[-1], 1,3), col=colMutations)
u <- par("usr")
par(xpd=NA)
text(u[1]+2,u[4], "upregulated", pos=4, cex=.7)
text(u[1]+2,u[3]+5, "downregulated", pos=4, cex=.7)
n <- sub("[0-9]+ ","",gsub("_"," ",rownames(chromHmmDown)[o]))
legend("right", bty="n", fill=c(NA,colChromHmm[!duplicated(n)]), c("Chromatin state:",unique(n)), cex=.8, border=NA, y.intersp=.8, x.intersp=0.2 )
```

### 3. NIH roadmap histone modification data

We need the following bigwig files from GEO

```
files <- dir("GSE19465", pattern="GSM48670", full.names = TRUE)
files
```

```
## [1] "GSE19465/GSM486702_BI.CD34_Primary_Cells.Input.CD34_39661.bw"   
## [2] "GSE19465/GSM486704_BI.CD34_Primary_Cells.H3K27me3.CD34_39661.bw"
## [3] "GSE19465/GSM486706_BI.CD34_Primary_Cells.H3K27me3.CD34_39804.bw"
```

The above files have been downloaded from GEO (http://www.ncbi.nlm.nih.gov/geo/) into the directory `GSE19465` as wiggle tracks and converted into bigwig using wigToBigWig.pl for fast random access.
The GSM identifiers will help find them.

```
getTssEnrichment <- function(files, tss){
    sapply(files, function(f){
                wig <- import(f,"bigwig", which=tss)
                o <- findOverlaps(tss, wig)
                sapply(1:length(tss), function(h)
                            sum(wig[subjectHits(o)[queryHits(o)==h]]$score))
            })
}
tmp <- na.omit(AnnotationDbi::select(org.Hs.eg.db, rownames(geneExpr), c("CHR","CHRLOC","CHRLOCEND","ENTREZID")))
tmp <- tmp[tmp$CHR != "Un",]
tss <- sort(GRanges(paste("chr",tmp$CHR,sep=""), shift(IRanges(ifelse(tmp$CHRLOC >0 , tmp$CHRLOC, -tmp$CHRLOCEND), width=1000),-500), entrezid=tmp$ENTREZID)) ## All TSS of mapped genes +/- 500bp
h3k27 <- list(random = getTssEnrichment(files, tss[sample(length(tss), 1000)]),
        EZH2 = getTssEnrichment(files, tss[tss$entrezid %in% rownames(glm)[testResults[,"EZH2"]!=0]]),
        ASXL1 = getTssEnrichment(files, tss[tss$entrezid %in% rownames(glm)[testResults[,"ASXL1"]!=0]]))
```

Compute enrichment

```
h3k27Enrichment <- sapply(h3k27, function(x)
            log10(rowSums(x[,2:3])/50 + .5)-log10(x[,1]/50+.5))
```

```
par(bty="n", mgp = c(2,0.5,0), las=1, tcl=-.25, font.main=3, mar=c(3,3,1,0.5)+.1)
boxplot(h3k27Enrichment, yaxt="n",xaxt="n", boxwex=0.66, outline=FALSE, lty=1, outwex=0, staplewex=0, border=c("grey", colMutations[c("EZH2","ASXL1")]), log="", ylab="H3K27me3 TSS signal", ylim=c(-1,1.5))
a <- axTicks(side=2, axp=c(0.1,1,3),log=TRUE)
axis(side=2, at = log10(a), labels=a)
u <- par("usr")
rotatedLabel(1:3, rep(-1,3), c("Random", "EZH2", "ASXL1"), font=c(1,3,3))
p <- c(t.test(h3k27Enrichment$random, h3k27Enrichment$EZH2)$p.value,
        t.test(h3k27Enrichment$random, h3k27Enrichment$ASXL1)$p.value)
axis(3, at=c(1,2), labels=NA, tcl=0.25, line=-.5)
mtext(side=3, at=1.5,  sig2star(p[1]), line=-.85)
axis(3, at=c(1,3), labels=NA, tcl=0.25, line=0)
mtext(side=3, at=2,  sig2star(p[2]), line=-.35)
```

```
par(bty="n", mgp = c(1.75,0.5,0), las=1, tcl=-.25, font.main=3, mar=c(1,2.5,1,0.5)+.1)
boxplot(glm$coefficients[,1],
        glm$coefficients[testResults[,"EZH2"]!=0,1],
        glm$coefficients[testResults[,"EZH2"]!=0,1]+glm$coefficients[testResults[,"EZH2"]!=0,"EZH2"],
        glm$coefficients[testResults[,"ASXL1"]!=0,1], 
        glm$coefficients[testResults[,"ASXL1"]!=0,1] +  glm$coefficients[testResults[,"ASXL1"]!=0,"ASXL1"], 
        xaxt="n", boxwex=0.66, outline=TRUE, lty=1, outwex=1, staplewex=0, pch=16, cex=.5,
        border=c("grey", colMutations[rep(c("EZH2","ASXL1"), each=2)]), log="", ylab="Average target expression", ylim=c(1,16))
segments(2,     glm$coefficients[testResults[,"EZH2"]!=0,1],
        3, glm$coefficients[testResults[,"EZH2"]!=0,1]+glm$coefficients[testResults[,"EZH2"]!=0,"EZH2"],
        col=paste(colMutations["EZH2"],"22",sep=""), lwd=.5,
)
segments(4,     glm$coefficients[testResults[,"ASXL1"]!=0,1],
        5, glm$coefficients[testResults[,"ASXL1"]!=0,1]+glm$coefficients[testResults[,"ASXL1"]!=0,"ASXL1"],
        col=paste(colMutations["ASXL1"],"22",sep=""), lwd=.5,
)
u <- par("usr")
axis(1, at=c(2,3), labels=c("wt","mt"), tcl=-0.25, line=-9, cex=.7)
axis(1, at=c(4,5), labels=c("wt","mt"), tcl=-0.25, line=-9, cex.lab=.3)
mtext(side=1,at=c(1, 2.5, 4.5), c("Any","EZH2","ASXL1"), line=-.5, font=c(1,3,3))
p <- c(t.test(glm$coefficients[,1],glm$coefficients[testResults[,"EZH2"]!=0,1])$p.value,
        t.test(glm$coefficients[,1],glm$coefficients[testResults[,"ASXL1"]!=0,1])$p.value)
axis(3, at=c(1,2), labels=NA, tcl=0.25, line=-.5)
s <- sig2star(p, breaks=10^c(-Inf,-3:0), labels=c("***","**","*",""))
mtext(side=3, at=1.5,  s[1], line=-.85, col=)
axis(3, at=c(1,4), labels=NA, tcl=0.25, line=0)
mtext(side=3, at=3,  s[2], line=-.35)
```

### 4. HB expression

The HGA and HGB loci show strong differences associated with mutations in SF3B1 and STAG2

```
par(bty="n", mgp = c(1.5,0.33,0), las=1, tcl=-.25, font.main=3, mar=c(3,2.5,1,3.5)+.1)
w <- intersect(unlist(AnnotationDbi::mget( grep("^hemoglobin,", Rkeys(org.Hs.egGENENAME), value=TRUE),revmap(org.Hs.egGENENAME))), rownames(glm))
x <- c(glmProjection[c("SF3B1","Normal","STAG2"),1],0)[c(1,4,2,3)]
y <- cbind(glm$coefficients[w,c("SF3B1","Normal","STAG2")], 0)[,c(1,4,2,3)] + glm$coefficients[w,"offset"]
plot(x,y[1,], pch=NA, xlab="", ylab="Predicted expression", ylim=range(y), xaxt="n")
u <- par("usr")
axis(side=1, at=x, labels=NA)
rotatedLabel(x, rep(u[3],4), c("SF3B1","MDS","Normal", "STAG2"), font=c(3,1,1,3), col=c(colMutations["SF3B1"],"#AAAAAA", colMutations[c("Normal","STAG2")]))
for(i in 1:nrow(y)){

    lines(x, y[i,], col=set1[1])
    o <- sqrt(c(glm$stdev.unscaled[w[i],c("SF3B1","Normal","STAG2")],0)[c(1,4,2,3)]^2 + glm$stdev.unscaled[w[i],1]^2)
    polygon(c(x,rev(x)), c(y[i,] + 2*o, rev(y[i,]) - 2*rev(o)), border=NA, col=paste(set1[1], "22",sep=""))
}
par(xpd=NA)
segments(x[4] + (u[2]-u[1])*.05, y[,4], x[4] + (u[2]-u[1])*.15, rank(y[,4]))
mtext(side=4, at=rank(y[,4]), text=select(org.Hs.eg.db, w, "SYMBOL")$SYMBOL, font=ifelse(select(org.Hs.eg.db, w, "CHR")$CHR==16, 4,3), las=2, cex=.7, line=1)
```

## 4. Clinical regression

```
mdsSamples <- mdsData$GEOID[ix]
subs <- mdsData[ix, grep("SF3B1|TET2|SRSF2|ASXL1|DNMT3A|RUNX1|U2AF1|TP53|EZH2|IDH2|STAG2|ZRSR2|CBL|BCOR|NRAS|JAK2|CUX1|IDH1|KRAS|PHF6|EP300|GATA2|NPM1|MLL2|PTPN11|CREBBP|KIT|MPL|NF1|WT1|IRF1|RAD21|ATRX|CDKN2A|ETV6|KDM6A|CEBPA|FLT3|GNAS|PTEN|SH2B3|BRAF|CTNNA1", colnames(mdsData))] # oncogenic mutations
subs <- subs[,colSums(subs)>2]
```

### 1. Define variable categories

```
Z <-  list()
Z$expression = scale(pca$x[mdsSamples,1:20])
Z$genetics = scale(subs+0, scale=FALSE)
Z$cytogenetics = scale(cytoImputed, scale=FALSE) #data[match(rownames(design), mds_clin$PDID),c("chr3","del5q","del7_7q","tri8","del11","del12","alt17q","tri19","del20q","complex")],
Z$geneticsExpression = cbind(Z$genetics,Z$expression)
Z$blood = scale(mdsData[ix,c("PB_cytopenia","plt_log","Haemoglobin","bm_blasts_logit","ring_sideroblasts_logit")])
Z$demographics = scale(mdsData[ix,c( "Gender", "Age")])
Z$clinical = cbind(Z$blood, Z$demographics)
Z$geneticsCytogenetics = cbind(Z$genetics, Z$cytogenetics)
Z$geneticsCytogeneticsExpression = cbind(Z$geneticsCytogenetics, Z$expression)
Z$expressionClinical = cbind(Z$expression, Z$clinical)
Z$geneticsClinical = cbind(Z$genetics, Z$clinical)
Z$geneticsExpressionClinical = cbind(Z$geneticsExpression, Z$clinical)
Z$all = cbind(Z$geneticsCytogenetics, Z$expression, Z$clinical)
```

### 2. Compute LASSO regression

```
Y <- cbind(mdsData[ix,c("PB_cytopenia","plt_log","Haemoglobin","bm_blasts_logit","ring_sideroblasts_logit", "Gender", "Age")],
                (mdsData[ix,c("anc_log","ME_ratio", "Serum_ferritin" )]))
colnames(Y) <- c("Cytopenia", "Platelets","Haemoglobin","Bonemarrow blasts","Ringed sideroblasts","Gender","Age","Absolute neutrophil count","M:E ratio","Serum ferritin")
Y <- Y[,order(colnames(Y))]
X <- scale(Z$geneticsCytogeneticsExpression, scale=TRUE)
for(j in 1:ncol(X))
    X[is.na(X[,j]),j] <- mean(X[,j], na.rm=TRUE)
set.seed(42)
clinModels = lapply(Y, function(y){
            if (class(y) %in% c("numeric","integer")){
                if(all(y %in% c(0,1,NA)))
                    cv.glmnet(X[!is.na(y),], na.omit(y), family = "binomial", alpha=1, standardize=FALSE, nfolds=5)
                else if(all(y %in% c(0,20,NA)))
                    cv.glmnet(X[!is.na(y),], na.omit(y), family = "poisson", alpha=1, standardize=FALSE, nfolds=5)
                else
                    cv.glmnet(X[!is.na(y),], na.omit(y), family = "gaussian", alpha=1, standardize=FALSE, nfolds=5)
            }
            else if (class(y)=="factor")
                cv.glmnet(X[!is.na(y),], na.omit(y), family="multinomial",  alpha=1, standardize=FALSE, nfolds=5)
        })
```

#### Plot

```
par(bty="n", mgp = c(2.5,.5,0), mar=c(2.5,4,2,4)+.1, las=2, tcl=-.25)
i = 1
n <- colnames(Z$geneticsCytogeneticsExpression)
annot <- 1 + grepl("^[A-Z]",n) + grepl("PC",n)
names(annot) <- n
for(m in clinModels){
    plotcvnet(m, Z$geneticsCytogeneticsExpression, main=names(clinModels)[i],  col0="black", cex=1, simple.annot = annot, col=set1[c(3,2,4)])
    i = i+1
    legend("topright", col=c(set1[c(1,3)],"black")[c(1,3,2)], c(expression(paste("Explained variance ",Rgenetics^2)), expression(paste("Lasso penalty ",lambda)), expression(paste("Model coefficient ", beta))), box.lty=0, bg="#FFFFFF33", pch=c(NA,NA,19), lty=c(1,1,NA), cex=.8, pt.cex = 1)
}
```

#### Heatmap of GLMs

Compute a matrix with all coefficients as well as the R2

```
j <- 0
z <- sapply(clinModels,function(x){
            j <<- j+1
            w <- which.min(x$cvm)
            c <- x$glmnet.fit$beta[,w]
            yj <- sapply(c("genetics","cytogenetics","expression"), function(i){
                        w <- colnames(Z[[i]])
                        X[,w] %*% c[w] 
                    })
            cj <- rowSums(cov(yj))
            y <- Y[,j] - x$glmnet.fit$a0[w]
            covj <- colMeans((y-mean(y))*(yj - rep(colMeans(yj), each=nrow(yj))))
            r2 <- cj
            R2 <- 1 - x$cvm[w]/x$cvm[1]
            c(c, NA,  r2/sum(r2), R2=R2)
        })
m <- nrow(z) #ncol(Z$geneticsCytogeneticsExpression)
z[1:m,] <- pmin(z[1:m,],1)
z[1:m,] <- pmax(z[1:m,],-.999)
z[z==0] <- NA
```

These are the ranks

```
r <- sapply(clinModels, function(x) rank(apply(x$glmnet.fit$beta,1, function(y) which(y!=0)[1]), ties="min"))
s <- sapply(clinModels, function(x) {
            w = which.min(x$cvm)
            w <- rev(which(x$cvm[1:w] > x$cvup[w]))[1] +1
            if(!is.na(w))
                sum(x$glmnet.fit$beta[,w]!=0)
            else
                0
        })
p <- sapply(clinModels, function(x) {w <- which.min(x$cvm); (1 - x$cvup[w]/x$cvm[1]) > 0 })
```

The explained variance R2

```
R2 <- z[nrow(z) - 3:0,]
R2[is.na(R2)] <- 0
z <- z[-(nrow(z) - 0:4),]
z <- z[,ncol(z):1]
r <- r[,ncol(r):1]
```

Plot

```
layout(matrix(c(1,2,3),1,3), c(6.5,.75,.75), 2, TRUE)
par(bty="n", mgp = c(3,.5,0), mar=c(4,10,2,0)+.1, las=1, tcl=-.25, cex=1)
w <- TRUE#rev(c("ring_sideroblasts_logit","bm_blasts_logit","hb", "plt_log", "pb_cytopenia", "age","sex"))
image(y=1:ncol(z)-.5, x=1:nrow(z), z[,w], breaks=c(-2,seq(-1,1,l=51)), col=c("grey",colorRampPalette(brewer.pal(9,"RdYlBu"))(50)), xaxt="n", yaxt="n", xlab="", ylab="", ylim=c(0,10))
abline(v=c(18.5,27.5), lwd=0.5)
rotatedLabel(y0=rep(0.5,nrow(z)), labels=sub("_","/",rownames(z)), x0=1:nrow(z), font=c(rep(3,18),rep(1,nrow(z)-18)), col = set1[c(3,2,4)][annot], cex=0.9)
mtext(side=2, line=.2, text=colnames(z), las=2, at=1:ncol(z)-.5)
text(y=rep(1:ncol(z)-.5, each=nrow(r)), x=rep(1:nrow(r), ncol(z)), r[,w] * (0!=(z[1:nrow(r),w])), cex=0.66, font=ifelse(r <= rep(s[ncol(r):1], each=nrow(r)), 2,1))
points(y=rep(1:ncol(z)-.5, each=nrow(r)), x=rep(1:nrow(r), ncol(z)), pch=ifelse(is.na(z) | z==0, ".",NA))
mtext(side=1, at=9, "Genetics", col=set1[2], line=2.5 )
mtext(side=1, at=23, "Cytogenetics", col=set1[3], line=2.5 )
mtext(side=1, at=38, "Expression", col=set1[4], line=2.5 )
mtext(side=3, "Model coefficients", at = 14, line=0.5)
clip(-10,50,0,15)
image(y=dim(z)[2] + c(0.5,1.5), x=1+ 1:7 , matrix(seq(-0.99,1,l=7), ncol=1), breaks=c(-2,seq(-1,1,l=51)), col=c("grey",colorRampPalette(brewer.pal(9,"RdYlBu"))(50)), xaxt="n", yaxt="n", xlab="", ylab="", add=TRUE)
text(y=dim(z)[2]+1, x=c(1,9), c(-1,1))
points(y=11,x=5, pch=".")
rect(19.5,10.5,20.5,11.5, lwd=0.5)
text(20,11,1, cex=0.66)
text(21, 11, "LASSO rank", pos=4)
u <- par("usr")
par(bty="n", mgp = c(3,.5,0), mar=c(4,1,2,0)+.1, las=1, tcl=-.25)
plot(NA,NA, xlab="", ylab="", xaxt="n", yaxt="n", ylim=c(0,10), xlim=c(0,1), yaxs="i")
barplot(R2[1:3,ncol(R2):1], border=NA, col=paste(set1[c(2,3,4)],"88",sep=""), horiz=TRUE, names.arg=rep(NA,ncol(R2)), width=0.95, space=0.0525, add=TRUE)
mtext(side=3, "Variance components", line=.5)
par(bty="n", mgp = c(3,.5,0), mar=c(4,1,2,1)+.1, las=1, tcl=-.25)
plot(NA,NA, xlab="", ylab="", xaxt="n", yaxt="n", ylim=c(0,10), xlim=c(0,.9), yaxs="i")
barplot(R2[4,ncol(R2):1], border=NA, col="grey", horiz=TRUE, names.arg=rep(NA,ncol(R2)), width=0.95, space=0.0525, add=TRUE) -> b
points(R2[4,ncol(R2):1]+0.1,b, pch=ifelse(rev(p),"*",NA))
mtext(side=1, expression(paste("Explained variance ",R^2)), line=2.5)
```

#### Prediction of ringed sideroblasts

As an example of the LASSO fits, show the prediction vs. observations of the proportion of ringed sideroblasts.

```
par(bty="n", mgp = c(1.5,.33,0), mar=c(2.5,3.5,2,1)+.1, las=1, tcl=-.25)
w <- which(clinModels$`Ringed sideroblasts`$glmnet.fit$beta[,which.min(clinModels$`Ringed sideroblasts`$cvm)]!=0)
X <- scale(Z$geneticsCytogeneticsExpression, scale=TRUE)
for(j in 1:ncol(X))
    X[is.na(X[,j]),j] <- mean(X[,j], na.rm=TRUE)
set.seed(42)
#l <- lm(Y$`Ringed sideroblasts` ~ X[,w])
#y <- na.omit(Y$`Ringed sideroblasts`)
#x <- predict(l)
c <- clinModels$`Ringed sideroblasts`$glmnet.fit$beta[,which.min(clinModels$`Ringed sideroblasts`$cvm)]
y <- na.omit(Y$`Ringed sideroblasts`)
x <- X[-na.action(y),] %*% c + mean(y)
invlogit <- function(x) 1/(1+exp(-x))
plot(x, y, xaxt="n", yaxt="n", pch=16, cex=0.8, xlab="", ylab="", xlim=car::logit(c(0.01,0.95)), ylim=car::logit(c(0.025,0.95)))
title(xlab="Predicted ringed sideroblasts")
title(ylab="Observed ringed sideroblasts", line=2.5)
abline(0,1)
u <- par("usr")
par(xpd=NA)
#y <- l$coefficients[-1]+ l$coefficients[1]
y <- c[w] / apply(Z$geneticsCytogeneticsExpression,2,sd, na.rm=TRUE)[w] + mean(y)
u <- par("usr")
x0 <- rep(u[4]-0.5,length(y))
names(x0) <- names(y)
y0 <- u[4] + 0.05*(u[4]-u[3]) - rank(-y)/length(y) * (u[4]-u[3])/1.2
d <- density(y)
lines(d$x, d$y/2-0.5 +u[4], col="grey")
lines(d$x, -d$y/2-0.5+u[4], col="grey")
points(x=y, y=x0+violinJitter(y, magnitude=1)$y, pch=16, col=set1[c(3,2,4)][annot][w]) #annot[w]/10-0.2
w <- r[w,"Ringed sideroblasts"]
w <- "SF3B1"#names(w[w<=5])
rotatedLabel(y[w], x0[w], labels=(w), font=ifelse(grepl("PC", w),1,3), cex=.66, pos=3, col=set1[c(3,2,4)][annot[(w)]])
axis(at=-1:1 + mean(y), labels=-1:1, side=3, cex.axis=.8)
mtext(at=mean(y), line=1, side=3, "Coefficients", cex=.8)
text(u[2],u[3]+1, expression(paste(R^2==0.55)), pos=2)
par(xpd=FALSE)
car::probabilityAxis(side="left", axis.title = "")
car::probabilityAxis(side="below", axis.title = "")
```

## 5. Survival analysis

Here we analyse the influence of mutations, expression and blood counts on survival

### 1. Prepare data

```
amlFreeSurvival <- Surv(time=mdsData$Survival_days, event=mdsData$Status)
amlFreeSurvival[!is.na(mdsData$AML_progression_days)] <- Surv(time=mdsData$AML_progression_days, event=mdsData$AML_status)[!is.na(mdsData$AML_progression_days)]
amlFreeSurvival[mdsData$AML_progression_days < 0 | mdsData$Survival_days < 0] <- NA
amlFreeSurvival <- amlFreeSurvival[ix]
amlFreeSurvival[,1] <- amlFreeSurvival[,1] /365*12 # Convert to months
```

#### Model

We use the following variant of the Cox proportional hazards model to estimate the survial models

```
ecoxph <- function(X, surv, tol=1e-3, max.iter=50){
    if(class(X)=="data.frame")
        X = as.matrix(X)
    beta0 = rep(0,ncol(X))
    beta1 = rep(1,ncol(X))
    sigma2 = 1
    iter = 1
    while(max(abs(beta1-beta0))>tol& iter < max.iter){
        fit = coxph(surv ~ ridge(X, theta=1/sigma2, scale=FALSE))
        sigma2 = (1 + sum((fit$coefficients-mean(fit$coefficients))^2))/(ncol(X))   
        beta0 = beta1
        beta1 = fit$coefficients
        #cat(beta1,"\n")
        #cat(sigma,"\n")
        iter = iter+1
    }
    fit$sigma2 = sigma2
    names(fit$coefficients) = colnames(X)
    return(fit)
}
```

This model uses a Gaussian prior on the coefficients. The variance of the prior is estimate by empirical Bayes.
The shared prior introduces a ridge penalty on the parameters, which help stabilise the estimates.

#### Impute missing Z by mean..

```
for(i in names(Z)){
    for(j in 1:ncol(Z[[i]])){
        Z[[i]][is.na(Z[[i]][,j]),j] <- mean(Z[[i]][,j], na.rm=TRUE)
    }
}
```

#### Concordance of models based on different covariates

Use five-fold cross validation

```
set.seed(42)
concordanceCV = data.frame()
cv_ix = sample(1:5, length(mdsSamples), replace=TRUE)
for(i in unique(cv_ix)){
    c = lapply(Z, function(x) ecoxph(x[cv_ix!=i,], amlFreeSurvival[cv_ix!=i] ))
    p = mapply(function(x,y) as.matrix(x[cv_ix==i,]) %*% coef(y), Z, c)
    concordanceCV = rbind(concordanceCV, apply(-p,2, rcorr.cens, amlFreeSurvival[cv_ix==i])[1,])
}
colnames(concordanceCV) = names(Z)
colMeans(concordanceCV)
```

```
##                     expression                       genetics                   cytogenetics 
##                         0.7561                         0.6770                         0.5039 
##             geneticsExpression                          blood                   demographics 
##                         0.7761                         0.6918                         0.6501 
##                       clinical           geneticsCytogenetics geneticsCytogeneticsExpression 
##                         0.7061                         0.6439                         0.7716 
##             expressionClinical               geneticsClinical     geneticsExpressionClinical 
##                         0.7578                         0.7080                         0.7605 
##                            all 
##                         0.7639
```

```
ipss <- mdsData$ipss[ix]
hIpss <- rcorr.cens(-ipss,amlFreeSurvival)[1]
```

Barplots

```
par(bty="n", mgp = c(2,.33,0), mar=c(5,3,1,1)+.1, las=1, tcl=-.25, xpd=NA)
h <- concordanceCV[,c("genetics","cytogenetics","expression","blood","demographics","all")]
v <- sapply(h, sd)/sqrt(5)
colnames(h) <- ""
b <- barplot(c(colMeans(h),hIpss), border=NA, col=paste(c(set1[c(2,3,4,1,5,7)],"#BBBBBB"),"88", sep=""), las=2, ylim=c(0,0.8), ylab="Harrel's C", names.arg=rep("",7))
points(rep(b[-7], each=5) + seq(-.1,.1,l=5), unlist(h), pch=16, cex=.5, col="darkgrey")
segments(x0=b[-7], y0=colMeans(h)-v, y1=colMeans(h)+v)
rotatedLabel(b, rep(0,7), c("Genetics","Cytogenetics","Expression","Blood counts","Demographics","All","IPSS"))
```

Kaplan-Meier plots of risk terciles

```
par(bty="n", mgp = c(1.9,.33,0), mar=c(2.3,3,2,2.1)+.1, las=1, tcl=-.25)
j <- 0
for(x in Z){
    j <- j +1
    c = sapply(1:5, function(i) coef(ecoxph(x[cv_ix!=i,],amlFreeSurvival[cv_ix!=i])))
    r = as.matrix(x) %*% rowMeans(c) 
    plot(survfit(amlFreeSurvival ~ cut(r, quantile(r+runif(length(r), -0.01,0.01), seq(0,1,l=4), na.rm=TRUE), right=FALSE)), col = set1[3:1], xlab="", ylab="AML-free survival")
    title(xlab="Months", line=1.5)
    mtext(capitalize(names(Z)[j]), font=2, at=40, side=3, line=0.5)
    par(xpd=TRUE)
    legend(x=60, y=1.2, bty="n", "Risk", cex=0.9, text.font=2)
    legend(x=60, y=1.1, bty="n", text.col = set1[3:1], c("Low","Intermediate","High"), cex=0.9)
    text(x=0, y=0.05, paste("C =", round(colMeans(concordanceCV)[j],2)), cex=1, pos=4)
}
```

### 2. Risk contributions

Covariates once more

```
X <-  data.frame(
        Genetics = Z$genetics,
        Cytogenetics = Z$cytogenetics,
        Expression= Z$expression,
        `Blood counts` = Z$blood,
        Demographics =  Z$demographics
)
```

#### Fit single model

```
groups <- sub("\\..+","",colnames(X))
model <- ecoxph(X, amlFreeSurvival)
index <- TRUE
r <- sapply(unique(groups[index]), function(x) {
            ix <- groups[index] == x
            as.matrix(X[,index][,ix, drop=FALSE]) %*% coef(model)[ix] #+ fullModel$sumX[,x] * fullModel$mu[x]
        })

c <- cov(r, use="complete")
x <-  colSums(c)/sum(c)#diag(c / sum(diag(c))) #
x <- x - sum(x[x < 0])
col0 <- c(paste(set1, "88", sep="")[c(3,2,4,1,5)], "grey")
pie(x, col=col0, border=NA, labels = paste(names(x), " (",round(100*x),"%)", sep=""))
polygon(cos(seq(0,2*pi,l=100))*.5, sin(seq(0,2*pi,l=100))*.5, col="white", border=NA)

C <- rcorr.cens(-rowSums(r), amlFreeSurvival)[1]
polygon(c(sin(seq(0,2*pi *C,l=100) +(1-C)*pi)*.4,0), c(cos(seq(0,2*pi *C,l=100)+(1-C)*pi)*.4,0), col="grey", border=NA)
text(0,-0.1, paste("C=",round(C,2), sep=""), col="white", pos=1)
```

#### Five-fold cross validation

It may be better to use cross-validation, especially for the estimate of concordance

```
C <- NULL
y <- NULL
set.seed(42)
cv_ix = sample(1:5, length(mdsSamples), replace=TRUE)

for(i in 1:5){
    fit = ecoxph(X[cv_ix!=i,index],amlFreeSurvival[cv_ix!=i], tol=1e-6)
    c = coef(fit)
    C = c(C,rcorr.cens(-as.matrix(X[cv_ix==i,index][,groups!="Nuisance"]) %*% c[groups!="Nuisance"] , amlFreeSurvival[cv_ix==i])[1])
    r <- sapply(unique(groups[index]), function(x) {
                ix <- groups[index] == x
                as.matrix(X[cv_ix!=i,index][,ix, drop=FALSE]) %*% coef(fit)[ix] #+ fullModel$sumX[,x] * fullModel$mu[x]
            })

    c <- cov(r, use="complete")
    x <-  colSums(c)/sum(c)#diag(c / sum(diag(c))) #
    y <- rbind(y,x)
}
meanC <- mean(C)
x <- colMeans(y)
x <- colSums(c)/sum(c)
x <- x - sum(x[x<0])
pi <- base::pi
```

```
par(bty="n", mgp = c(2,.33,0), mar=c(1,3,1,3)+.1, las=2, tcl=-.25)
pie(x, col=col0, border=NA, labels = paste(names(x), " (",round(100*x),"%)", sep=""), radius=.8, init.angle=179)
title(main="Survival risk contributions", font.main=1, cex.main=1)
polygon(cos(seq(0,2*pi,l=100))*.5, sin(seq(0,2*pi,l=100))*.5, col="white", border=NA)
polygon(c(sin(seq(0,2*pi *meanC,l=100) +(1-meanC)*pi)*.4,0), c(cos(seq(0,2*pi *meanC,l=100)+(1-meanC)*pi)*.4,0), col="grey", border=NA)
text(0,0, paste("C=",round(meanC,2), sep=""), col="white", pos=1)
```

### 3. Random Survival Forest

Here we compare the results of the previous section to a random survival forest

#### Fit model

```
rsf <- rfsrc(Surv(time,status) ~ ., data=cbind(time=amlFreeSurvival[,1], status=amlFreeSurvival[,2], X) )
col=set1[c(3,2,4,1,5)]#  c(brewer.pal(8, "Dark2")[1:3], brewer.pal(8, "Set1")[2:1])
```

#### Variable importance

```
par(bty="n", mgp = c(2,.33,0), mar=c(4,3,1,0.5)+.1, las=2, tcl=-.25, las=3, xpd=NA)
boxplot(rsf$importance ~ factor(groups, levels=unique(groups)), border= col, staplewex=0, pch=16, cex=0.75, ylab="Variable importance", lty=1, xaxt="n")
rotatedLabel(x0=1:5, y0=rep(-0.002,5), labels=unique(groups), srt=45)
```

The plot confirms the result that expression, blood counts, and clinical variables are most influental.
Another plot

```
par(bty="n", mgp = c(2,.33,0), mar=c(3,3,1,0.5)+.1, las=2, tcl=-.25, las=3, lend=1, xpd=NA)
o <- order(rsf$importance, decreasing = TRUE)
plot(rsf$importance[o], type="h", col=col[factor(groups, levels=unique(groups))[o]], ylab="Variable importance", xaxt="n", xlab="", lwd=7)
legend("topright", col=col, lty=1, unique(groups), bty="n", lwd=3)
```

```
par(bty="n", mgp = c(2,.33,0), mar=c(1,3,1,3)+.1, las=2, tcl=-.25)
plot.variable(rsf)
```

```
par(cex=0.75)
plot(rsf)
```

```
## 
##                                        Importance   Relative Imp
## Expression.PC13                            0.0229         1.0000
## Blood.counts.bm_blasts_logit               0.0091         0.3968
## Expression.PC16                            0.0090         0.3916
## Expression.PC19                            0.0053         0.2310
## Demographics.Gender                        0.0047         0.2059
## Expression.PC10                            0.0046         0.1995
## Blood.counts.plt_log                       0.0034         0.1490
## Genetics.RUNX1                             0.0032         0.1398
## Blood.counts.PB_cytopenia                  0.0030         0.1326
## Genetics.TP53                              0.0019         0.0833
## Expression.PC1                             0.0019         0.0824
## Expression.PC18                            0.0014         0.0628
## Expression.PC4                             0.0013         0.0581
## Demographics.Age                           0.0007         0.0318
## Genetics.TET2                              0.0007         0.0298
## Expression.PC6                             0.0006         0.0283
## Expression.PC7                             0.0006         0.0273
## Genetics.EZH2                              0.0005         0.0202
## Expression.PC2                             0.0004         0.0193
## Genetics.SRSF2                             0.0004         0.0192
## Genetics.BCOR                              0.0002         0.0094
## Genetics.DNMT3A                            0.0001         0.0061
## Cytogenetics..7.del.7q.                    0.0001         0.0031
## Cytogenetics.rearr.3q                      0.0000         0.0010
## Genetics.IDH2                              0.0000         0.0006
## Genetics.CUX1                              0.0000         0.0006
## Genetics.JAK2                              0.0000         0.0003
## Genetics.ZRSR2                             0.0000         0.0002
## Cytogenetics.del.Y.                        0.0000        -0.0011
## Genetics.PTPN11                            0.0000        -0.0019
## Cytogenetics.abn.17                       -0.0001        -0.0042
## Genetics.STAG2                            -0.0001        -0.0052
## Blood.counts.ring_sideroblasts_logit      -0.0001        -0.0055
## Genetics.PHF6                             -0.0001        -0.0057
## Cytogenetics.del.5q.                      -0.0001        -0.0059
## Cytogenetics.tri.19.                      -0.0001        -0.0063
## Expression.PC5                            -0.0002        -0.0067
## Cytogenetics.del.12p.                     -0.0002        -0.0074
## Expression.PC15                           -0.0002        -0.0075
## Genetics.CBL                              -0.0002        -0.0076
## Cytogenetics.del.20q.                     -0.0003        -0.0124
## Genetics.SF3B1                            -0.0003        -0.0136
## Genetics.U2AF1                            -0.0003        -0.0139
## Genetics.ASXL1                            -0.0005        -0.0237
## Cytogenetics.tri.8.                       -0.0005        -0.0238
## Expression.PC3                            -0.0008        -0.0364
## Expression.PC12                           -0.0009        -0.0403
## Expression.PC14                           -0.0010        -0.0453
## Blood.counts.Haemoglobin                  -0.0011        -0.0461
## Expression.PC8                            -0.0011        -0.0482
## Expression.PC11                           -0.0015        -0.0642
## Expression.PC9                            -0.0018        -0.0807
## Expression.PC20                           -0.0020        -0.0885
## Expression.PC17                           -0.0031        -0.1358
```

## Session

```
sessionInfo()
```

```
## R version 3.0.2 Patched (2013-11-29 r64352)
## Platform: x86_64-unknown-linux-gnu (64-bit)
## 
## locale:
##  [1] LC_CTYPE=en_GB.UTF-8       LC_NUMERIC=C               LC_TIME=en_GB.UTF-8        LC_COLLATE=en_GB.UTF-8    
##  [5] LC_MONETARY=en_GB.UTF-8    LC_MESSAGES=en_GB.UTF-8    LC_PAPER=en_GB.UTF-8       LC_NAME=C                 
##  [9] LC_ADDRESS=C               LC_TELEPHONE=C             LC_MEASUREMENT=en_GB.UTF-8 LC_IDENTIFICATION=C       
## 
## attached base packages:
##  [1] splines   grid      parallel  stats     graphics  grDevices utils     datasets  methods   base     
## 
## other attached packages:
##  [1] GO.db_2.10.1           randomForestSRC_1.4    Hmisc_3.14-4           Formula_1.1-1          lattice_0.20-29       
##  [6] survival_2.37-7        glmnet_1.9-8           Matrix_1.1-4           biomaRt_2.18.0         rtracklayer_1.22.7    
## [11] GenomicFeatures_1.14.5 GenomicRanges_1.14.4   XVector_0.2.0          IRanges_1.20.7         VennDiagram_1.6.7     
## [16] hgu133plus2.db_2.10.1  gcrma_2.34.0           affy_1.40.0            RColorBrewer_1.0-5     org.Hs.eg.db_2.10.1   
## [21] RSQLite_0.11.4         DBI_0.2-7              AnnotationDbi_1.24.0   Biobase_2.22.0         BiocGenerics_0.8.0    
## [26] limma_3.18.13          knitr_1.6             
## 
## loaded via a namespace (and not attached):
##  [1] affyio_1.30.0         BiocInstaller_1.12.1  Biostrings_2.30.1     bitops_1.0-6          BSgenome_1.30.0      
##  [6] car_2.0-20            cluster_1.15.2        digest_0.6.4          evaluate_0.5.5        formatR_0.10         
## [11] latticeExtra_0.6-26   MASS_7.3-33           nnet_7.3-8            preprocessCore_1.24.0 RCurl_1.95-4.1       
## [16] Rsamtools_1.14.3      stats4_3.0.2          stringr_0.6.2         tools_3.0.2           XML_3.98-1.1         
## [21] zlibbioc_1.8.0
```
