# Supplementary material for: Combining gene mutation with gene expression data improves outcome prediction in myelodysplastic syndromes
Source: Nat Commun. 2015 Jan 9;6:5901. doi: 10.1038/ncomms6901 (PMC4338540; doi:10.1038/ncomms6901)
Supplement: Supplementary Data 4 — TCGA AML analysis report [file ncomms6901-s5.zip › ncomms6901-s5.html]

Supplementary Data 4

# Supplementary Data 4

### Supplementary code and figures accompanying *Combining gene mutation with gene expression data improves outcome prediction in myelodysplastic syndromes*


This document contains the complete code used in the TCGA AML analysis. It is purely written in `R` using a series of `R` and `Bioconductor` packages.
This report has been generated using the `knitr` R package (http://yihui.name/knitr/).
For a complete list of packages and their versions please have a look at the end of this document.

#### Libraries

```
library(limma)
library(org.Hs.eg.db)
```

```
## Loading required package: AnnotationDbi
## Loading required package: BiocGenerics
## Loading required package: parallel
## 
## Attaching package: 'BiocGenerics'
## 
## The following objects are masked from 'package:parallel':
## 
##     clusterApply, clusterApplyLB, clusterCall, clusterEvalQ, clusterExport, clusterMap, parApply,
##     parCapply, parLapply, parLapplyLB, parRapply, parSapply, parSapplyLB
## 
## The following object is masked from 'package:stats':
## 
##     xtabs
## 
## The following objects are masked from 'package:base':
## 
##     anyDuplicated, append, as.data.frame, as.vector, cbind, colnames, duplicated, eval, Filter, Find, get,
##     intersect, lapply, Map, mapply, match, mget, order, paste, pmax, pmax.int, pmin, pmin.int, Position,
##     rank, rbind, Reduce, rep.int, rownames, sapply, setdiff, sort, table, tapply, union, unique, unlist
## 
## Loading required package: Biobase
## Welcome to Bioconductor
## 
##     Vignettes contain introductory material; view with 'browseVignettes()'. To cite Bioconductor, see
##     'citation("Biobase")', and for packages 'citation("pkgname")'.
## 
## Loading required package: DBI
```

```
library(hgu133plus2.db )
```

```
##
```

```
library(RColorBrewer)
set1 <- brewer.pal(9,"Set1")
library(cgdsr)
library(CoxHD) ## From github.com/mg14/CoxHD
```

```
## Loading required package: glmnet
## Loading required package: Matrix
## Loading required package: lattice
## Loaded glmnet 1.9-3
## 
## Loading required package: survival
## Loading required package: splines
## Loading required package: MASS
## 
## Attaching package: 'MASS'
## 
## The following object is masked from 'package:AnnotationDbi':
## 
##     select
```

```
library(mg14) ## From github.com/mg14/mg14
library(xtable)
library(Hmisc)
```

```
## Loading required package: Formula
## Hmisc library by Frank E Harrell Jr
## 
## Type library(help='Hmisc'), ?Overview, or ?Hmisc.Overview')
## to see overall documentation.
## 
## 
## Attaching package: 'Hmisc'
## 
## The following object is masked from 'package:xtable':
## 
##     label, label<-
## 
## The following object is masked from 'package:survival':
## 
##     survfitKM, untangle.specials
## 
## The following object is masked from 'package:AnnotationDbi':
## 
##     contents
## 
## The following object is masked from 'package:Biobase':
## 
##     combine, contents
## 
## The following object is masked from 'package:BiocGenerics':
## 
##     combine
## 
## The following object is masked from 'package:base':
## 
##     format.pval, round.POSIXt, trunc.POSIXt, units
```

## 1. Differential expression analysis

### 1.1 Load data

#### Get entrez ids for genes of interest, take all ids from the hg133plus array to have comparable data.

```
entrez <-  unique(AnnotationDbi::select(hgu133plus2.db, keys = keys(hgu133plus2.db), columns = c("ENTREZID"))$ENTREZID)
```

#### Load expression data from cBio portal

```
mycgds <-  CGDS("http://www.cbioportal.org/public-portal/")
tcgaAML <- getCancerStudies(mycgds)[1,1]
cases <- getCaseLists(mycgds,tcgaAML)[8,1]
g <-  lapply(split(as.numeric(entrez), seq_along(entrez)%/%500), function(genes) getProfileData(mycgds,genes,getGeneticProfiles(mycgds,tcgaAML)[2,1],cases)) ## load in batches of 500
g <- do.call("cbind", g)
tcgaExpr <- log(t(g)+.5)
rownames(tcgaExpr) <- sub("\\.","-",sub("^[0-9]+\\.","",rownames(tcgaExpr))) ## Fix rownames
#rm(e,s,g)
```

The query will have dropped a few genes. Match back to entrezids…

```
s <- AnnotationDbi::select(org.Hs.eg.db, rownames(tcgaExpr), "ENTREZID", "SYMBOL")
```

```
## Warning: 'select' and duplicate query keys resulted in 1:many mapping between keys and return rows
```

```
m <- match(rownames(tcgaExpr), s$SYMBOL)
sum(is.na(m))
```

```
## [1] 0
```

```
rownames(tcgaExpr) <- s$ENTREZID[m]
colnames(tcgaExpr) <- gsub("\\.","-",colnames(tcgaExpr))
tcgaExpr <- tcgaExpr[rowSums(is.na(tcgaExpr))==0 & !is.na(rownames(tcgaExpr)),]
```

Check the log2 FPKM densities across samples

```
plot(density(tcgaExpr[,1]))
for(j in 1:ncol(tcgaExpr)) lines(density(tcgaExpr[,j]))
```

#### Load curated clinical and mutation data

```
tcgaClinical <- read.table("SuppTableS3_Clinical.txt", sep="\t", header=TRUE, comment.char = "#") ## tab-delimietd version of Supplementary Table S3, Clinical sheet.
colnames(tcgaClinical)[3:12] <- capitalize(colnames(tcgaClinical)[3:12])
rownames(tcgaClinical) <- as.character(tcgaClinical$TCGA_ID)
tcgaGenetic <- read.table("SuppTableS3_Genetic.txt", sep="\t", header=TRUE, comment.char = "#") ## tab-delimietd version of Supplementary Table S3, Genetics sheet.
tcgaGenetic$TCGA_ID <- factor(as.character(tcgaGenetic$TCGA_ID), levels = levels(tcgaClinical$TCGA_ID))
g <- as.character(tcgaGenetic$Hugo_Symbol)
g[tcgaGenetic$Hugo_Symbol=="FLT3" & tcgaGenetic$Variant_Type == 'INS'] <- "FLT3_ITD"
g[tcgaGenetic$Hugo_Symbol=="FLT3" & tcgaGenetic$Variant_Type == 'SNP'] <- "FLT3_TKD"
tcgaMutation <- (table(tcgaGenetic$TCGA_ID,g)) > 0
```

### 1.2 Compute PCA

```
tcgaPca <- prcomp(t(tcgaExpr))
```

#### Covariates

```
t <- cbind(tcgaMutation +0, tcgaClinical[,14:24])
t <- t[,colSums(t[colnames(tcgaExpr),],na.rm=TRUE)>=5]
tcgaCovariates <- as.matrix(cbind(Offset=1,t, Gender=tcgaClinical$Gender, Age=tcgaClinical$AOD/10))[colnames(tcgaExpr),]
groups <- factor(c("Offset", rep("Genetics",22), "Translocations",rep("CNA", 5), rep("Translocations",2), rep("Demographics",2)), levels=c("Offset","Genetics","CNA","Translocations","Demographics"))
tcgaCovariates <- tcgaCovariates[,order(groups)]
groups <- groups[order(groups)]
col1 <- c("grey",set1[c(3,5,2,7)])
names(col1) <- levels(groups)
```

#### PCA overview

Now plot a large panel with small multiples of the first two PCs overlaid with the mutation status of each gene.

```
par(bty="n", mgp = c(0,0.5,0), mar=c(1,1,1.5,0)+.1, las=1, tcl=-.25, font.main=3, mfrow=c(6,6), xpd=NA)
i<-0
for (geneId in colnames(tcgaCovariates)[-1]){
    i<-i+1
    plot(tcgaPca$x[rownames(tcgaCovariates),], cex=0.5, 
            pch=NA, 
            xlab=ifelse(i==1,"PC1",""), ylab=ifelse(i==1,"PC2",""),main=geneId, font.main=ifelse(grepl("[[:lower:]]",geneId),1,3), cex.main=1.33, cex.lab=1.2,xaxt="n", yaxt="n", ylim=c(-65,65))
    if(geneId != "Age")
        w <- rownames(tcgaCovariates)[which(tcgaCovariates[,geneId] == 1)]
    else
        w <- rownames(tcgaCovariates)[which(tcgaCovariates[,geneId] > median(tcgaCovariates[,geneId], na.rm=TRUE))]
    points(tcgaPca$x[!rownames(tcgaPca$x) %in% w,], pch=ifelse(is.na(tcgaCovariates[!rownames(tcgaPca$x) %in% w,geneId]),1,19), cex=0.5, col="grey", lwd=0.5)
    points(tcgaPca$x[w,], pch=16, cex=0.85, col=col1[groups[i+1]], lwd=0.05)
    u <- matrix(par("usr"), ncol=2)
    if(i==1){
        arrows(u[1,1],u[1,2], u[2,1],u[1,2],length=0.02)
        arrows(u[1,1],u[1,2], u[1,1],u[2,2],length=0.02)
    }
    text(u[2],u[3] + (u[4]-u[3])*.9, labels=paste("n=",length(w), sep=""), bty="n", cex=1.2, pos=2)
}
plot.new()
legend("center",c("Missing","Wildtype","Mutant"), pch=c(1,19,19), col=c("grey","grey","black"), bty="n", pt.cex=c(0.5,0.5,rep(0.85,4)),cex=1.2, pt.lwd=0.5)
```

### 1.2 Explained variance R2/F-statistic

Here we want to determine all genes which are associated with any covariate. This will be based on an F-statistic.
lmFit also tests wether the offset is different from zero (trivially true).
Create design matrix of covariates; impute missing values by mean.

```
poorMansImpute <- function(x) {x[is.na(x)] <- mean(x, na.rm=TRUE); return(x)}
tcgaDesign <- apply(tcgaCovariates,2,poorMansImpute)
```

#### Fit the linear model

```
tcgaGlm = lmFit(as.matrix(tcgaExpr), design = tcgaDesign) 
tcgaGlm = eBayes(tcgaGlm)
```

#### Random model

Compare to a model where all values of the covariates are randomly permuted. This helps reassure FDR estimates.

```
set.seed(42)
tcgaRlm <- lmFit(tcgaExpr[,rownames(tcgaDesign)], apply(tcgaDesign, 2, sample))
tcgaRlm <- eBayes(tcgaRlm)
F.stat <- classifyTestsF(tcgaRlm[,-1],fstat.only=TRUE)
tcgaRlm$F <- as.vector(F.stat)
df1 <- attr(F.stat,"df1")
df2 <- attr(F.stat,"df2")
if(df2[1] > 1e6){ # Work around bug in R 2.1
    tcgaRlm$F.p.value <- pchisq(df1*tcgaRlm$F,df1,lower.tail=FALSE)
}else
    tcgaRlm$F.p.value <- pf(tcgaRlm$F,df1,df2,lower.tail=FALSE)
```

#### Explained variance by different categories

The F-statistic is directly related to the R2.

```
F.stat <- classifyTestsF(tcgaGlm[,2:22],fstat.only=TRUE) ## All genetics & cytogenetics
df1 <- attr(F.stat,"df1")
df2 <- attr(F.stat,"df2")
F.p.value <- pchisq(df1*F.stat,df1,lower.tail=FALSE)

R.stat <- classifyTestsF(tcgaRlm[,2:22],fstat.only=TRUE) ## Random

Rall = 1 - 1/(1 + tcgaGlm$F * (ncol(tcgaDesign)-1)/(nrow(tcgaDesign)-ncol(tcgaDesign)))
Rgenetics = 1 - 1/(1 + F.stat * 21/(nrow(tcgaDesign)-ncol(tcgaDesign)))
Pgenetics = 1 - 1/(1 + R.stat * 21/(nrow(tcgaDesign)-ncol(tcgaDesign)))
names(Rgenetics) <- names(Pgenetics) <- names(Rall) <-  rownames(tcgaExpr)
```

#### Plot the variance explained by genetics

```
par(bty="n", mgp = c(2,.33,0), mar=c(3,2.5,1,1)+.1, las=1, tcl=-.25, xpd=NA)
d <- density(Pgenetics,bw=1e-3)
f <- 1#nrow(gexpr)/512
plot(d$x, d$y * f, col='grey', xlab=expression(paste("Explained variance per gene ", R^2)), main="", lwd=2, type="l", ylab="", xlim=c(0,0.7))
title(ylab="Density", line=1.5)
d <- density(Rgenetics, bw=1e-3)
r <- min(Rgenetics[p.adjust(F.p.value,"BH")<0.05])
x0 <- which(d$x>r)
polygon(d$x[c(x0[1],x0)], c(0,d$y[x0])* f, col=paste(set1[1],"44",sep=""), border=NA)
lines(d$x, d$y* f, col=set1[1], lwd=2)
#points(d$x[x0[1]], d$y[x0[1]]*f, col=set1[1], pch=16)
text(d$x[x0[1]], d$y[x0[1]]*f, pos=4, paste(sum(Rgenetics > r), "genes q < 0.05"))
legend("topright", bty="n", col=c(set1[1], "grey"), lty=1, c("Observed","Random"), lwd=2)
```

#### Predictions

```
glmPrediction <- tcgaGlm$coefficients %*% t(tcgaDesign)
rlmPrediction <- tcgaRlm$coefficients %*% t(tcgaDesign)
```

### 1.4. Test results

Prepare the test results using a hierarchical procedure:
1. Adjust down transcripts using F-stat
2. Adjust along covariates

```
testResults <- decideTests(tcgaGlm, method="hierarchical",adjust.method="BH", p.value=0.05)[,-1]
significantGenes <- sapply(1:ncol(testResults), function(j){
            c <- tcgaGlm$coefficients[testResults[,j]!=0,j+1]
            table(cut(c, breaks=c(-5,seq(-1.5,1.5,l=7),5)))
        })
colnames(significantGenes) <- colnames(testResults)
```

#### Table of top 50 deregulated genes contains many HOX genes

```
t <- head(sort(Rgenetics, d=TRUE), 50)
g <- apply(testResults[names(t),], 1, function(x) paste(colnames(testResults)[x!=0], collapse=", "))
print(xtable(cbind(AnnotationDbi::select(org.Hs.eg.db, names(t), c("SYMBOL","GENENAME"), "ENTREZID"),`R^2`=t, `Mutations`=g)), type="html", )
```


|  | ENTREZID | SYMBOL | GENENAME | R^2 | Mutations |
| --- | --- | --- | --- | --- | --- |
| 7477 | 7477 | WNT7B | wingless-type MMTV integration site family, member 7B | 0.69 | NPM1 |
| 113802 | 113802 | HENMT1 | HEN1 methyltransferase homolog 1 (Arabidopsis) | 0.62 | CEBPA, DNMT3A, IDH1, IDH2, NPM1 |
| 4010 | 4010 | LMX1B | LIM homeobox transcription factor 1, beta | 0.56 | NPM1, TET2 |
| 7200 | 7200 | TRH | thyrotropin-releasing hormone | 0.56 | CEBPA, IDH1, NPM1, t\_15\_17, t\_8\_21, inv16\_t16\_16 |
| 3213 | 3213 | HOXB3 | homeobox B3 | 0.55 | CEBPA, NPM1, RUNX1, TP53, minus5\_5q, t\_15\_17, t\_8\_21 |
| 3214 | 3214 | HOXB4 | homeobox B4 | 0.55 | CEBPA, NPM1, RUNX1, TP53, minus5\_5q, plus8\_8q, t\_15\_17, t\_8\_21 |
| 3205 | 3205 | HOXA9 | homeobox A9 | 0.54 | CEBPA, KIT, NPM1, TP53, t\_15\_17, t\_8\_21, inv16\_t16\_16 |
| 159296 | 159296 | NKX2-3 | NK2 homeobox 3 | 0.54 | CEBPA, NPM1, t\_15\_17, t\_8\_21, inv16\_t16\_16 |
| 6678 | 6678 | SPARC | secreted protein, acidic, cysteine-rich (osteonectin) | 0.53 | FLT3\_TKD, NPM1, RUNX1, minus7q, t\_15\_17, inv16\_t16\_16 |
| 7102 | 7102 | TSPAN7 | tetraspanin 7 | 0.53 | CEBPA, FLT3\_TKD, NPM1, RUNX1, plus8\_8q, t\_8\_21, inv16\_t16\_16 |
| 3203 | 3203 | HOXA6 | homeobox A6 | 0.53 | CEBPA, NPM1, complex, t\_15\_17, t\_8\_21, inv16\_t16\_16 |
| 189 | 189 | AGXT | alanine-glyoxylate aminotransferase | 0.53 | FLT3\_ITD, IDH1, KIT, NPM1, plus8\_8q, t\_15\_17, t\_8\_21, inv16\_t16\_16 |
| 3206 | 3206 | HOXA10 | homeobox A10 | 0.53 | CEBPA, KIT, NPM1, t\_15\_17, t\_8\_21, inv16\_t16\_16 |
| 3212 | 3212 | HOXB2 | homeobox B2 | 0.53 | CEBPA, NPM1, RUNX1, TP53, minus5\_5q, minus7q, plus8\_8q, t\_15\_17, t\_8\_21 |
| 10882 | 10882 | C1QL1 | complement component 1, q subcomponent-like 1 | 0.52 | NPM1 |
| 3216 | 3216 | HOXB6 | homeobox B6 | 0.52 | CEBPA, NPM1, t\_15\_17, t\_8\_21 |
| 3215 | 3215 | HOXB5 | homeobox B5 | 0.52 | CEBPA, NPM1, t\_15\_17 |
| 3219 | 3219 | HOXB9 | homeobox B9 | 0.51 | NPM1 |
| 2788 | 2788 | GNG7 | guanine nucleotide binding protein (G protein), gamma 7 | 0.51 | NPM1, RUNX1 |
| 26040 | 26040 | SETBP1 | SET binding protein 1 | 0.50 | RUNX1 |
| 126259 | 126259 | TMIGD2 | transmembrane and immunoglobulin domain containing 2 | 0.50 | CEBPA, FLT3\_TKD, NPM1, STAG2, t\_15\_17, t\_8\_21, inv16\_t16\_16 |
| 4063 | 4063 | LY9 | lymphocyte antigen 9 | 0.50 | FLT3\_TKD, NPM1, RUNX1 |
| 4211 | 4211 | MEIS1 | Meis homeobox 1 | 0.50 | CEBPA, NPM1, minus7q, t\_15\_17, t\_8\_21 |
| 3204 | 3204 | HOXA7 | homeobox A7 | 0.49 | CEBPA, NPM1, t\_15\_17, t\_8\_21, inv16\_t16\_16 |
| 91752 | 91752 | ZNF804A | zinc finger protein 804A | 0.49 | KRAS, NPM1, PHF6, RUNX1 |
| 54491 | 54491 | FAM105A | family with sequence similarity 105, member A | 0.49 | NPM1 |
| 4300 | 4300 | MLLT3 | myeloid/lymphoid or mixed-lineage leukemia (trithorax homolog, Drosophila); translocated to, 3 | 0.49 | NPM1, RUNX1, minus7q, t\_15\_17 |
| 2255 | 2255 | FGF10 | fibroblast growth factor 10 | 0.49 | NPM1 |
| 4345 | 4345 | CD200 | CD200 molecule | 0.49 | NPM1, RUNX1, t\_8\_21 |
| 3202 | 3202 | HOXA5 | homeobox A5 | 0.49 | CEBPA, NPM1, complex, t\_15\_17, t\_8\_21, inv16\_t16\_16 |
| 84557 | 84557 | MAP1LC3A | microtubule-associated protein 1 light chain 3 alpha | 0.49 | CEBPA, IDH1, IDH2, NPM1, RUNX1 |
| 3200 | 3200 | HOXA3 | homeobox A3 | 0.49 | CEBPA, NPM1, t\_15\_17, t\_8\_21, inv16\_t16\_16 |
| 3959 | 3959 | LGALS3BP | lectin, galactoside-binding, soluble, 3 binding protein | 0.49 | CEBPA, FLT3\_ITD, IDH1, NPM1, complex, minus5\_5q, minus7q |
| 54549 | 54549 | SDK2 | sidekick cell adhesion molecule 2 | 0.49 | NPM1, RUNX1, t\_15\_17 |
| 387640 | 387640 | SKIDA1 | SKI/DACH domain containing 1 | 0.48 | CEBPA, DNMT3A, NPM1, inv16\_t16\_16 |
| 92235 | 92235 | DUSP27 | dual specificity phosphatase 27 (putative) | 0.48 | CEBPA, NPM1, RUNX1, TP53, inv16\_t16\_16 |
| 6196 | 6196 | RPS6KA2 | ribosomal protein S6 kinase, 90kDa, polypeptide 2 | 0.48 | CEBPA, NPM1, RUNX1, t\_15\_17, inv16\_t16\_16 |
| 5533 | 5533 | PPP3CC | protein phosphatase 3, catalytic subunit, gamma isozyme | 0.48 | FLT3\_TKD, NPM1, RUNX1, minus7q, plus8\_8q, t\_15\_17 |
| 780 | 780 | DDR1 | discoidin domain receptor tyrosine kinase 1 | 0.48 | CEBPA, KIT, RAD21, RUNX1, minus7q |
| 55614 | 55614 | KIF16B | kinesin family member 16B | 0.48 | CEBPA, IDH1, RUNX1, Age |
| 91010 | 91010 | FMNL3 | formin-like 3 | 0.48 | NPM1, RAD21, RUNX1, SMC1A |
| 221883 | 221883 | HOXA11-AS | HOXA11 antisense RNA | 0.48 | CEBPA, NPM1, RUNX1, TET2, complex, t\_15\_17, inv16\_t16\_16 |
| 100271722 | 100271722 | LINC00899 | long intergenic non-protein coding RNA 899 | 0.47 | CEBPA, NPM1, STAG2, plus8\_8q, t\_15\_17, inv16\_t16\_16, Age |
| 100129583 | 100129583 | FAM47E | family with sequence similarity 47, member E | 0.47 | FLT3\_ITD |
| 8412 | 8412 | BCAR3 | breast cancer anti-estrogen resistance 3 | 0.47 | FLT3\_TKD, NPM1, RUNX1, t\_8\_21 |
| 5463 | 5463 | POU6F1 | POU class 6 homeobox 1 | 0.47 | FLT3\_ITD, NPM1, t\_15\_17 |
| 3201 | 3201 | HOXA4 | homeobox A4 | 0.47 | CEBPA, NPM1, t\_15\_17, t\_8\_21, inv16\_t16\_16 |
| 9899 | 9899 | SV2B | synaptic vesicle glycoprotein 2B | 0.47 | IDH1, IDH2, NPM1 |
| 642938 | 642938 | FAM196A | family with sequence similarity 196, member A | 0.47 | CEBPA, FLT3\_ITD, IDH1, KRAS, RUNX1, minus7q, t\_15\_17, t\_8\_21 |
| 5746 | 5746 | PTH2R | parathyroid hormone 2 receptor | 0.47 | IDH1, IDH2, PHF6, RUNX1 |

#### Top genes

```
par(bty="n", mgp = c(1.5,.33,0), mar=c(2.5,2.5,1,1)+.1, las=1, tcl=-.25)
for(w in names(head(sort(Rgenetics, decreasing=TRUE),4))){
gene <- AnnotationDbi::select(org.Hs.eg.db, w, "SYMBOL", "ENTREZID")$SYMBOL
plot(glmPrediction[w,], tcgaExpr[w,rownames(tcgaDesign)], ylab=parse(text=paste("Observed  ~ italic(",gene,") ~ expression")), xlab=parse(text=paste("Predicted  ~ italic(",gene,") ~ expression")), pch=16, cex=.8)
par(xpd=FALSE)
abline(0,1)
u <- par("usr")
par(xpd=NA)
y <- tcgaGlm$coefficients[w,-1]+tcgaGlm$coefficients[w,1]
u <- par("usr")
x0 <- rep(u[4]-(u[4]-u[3])/8,ncol(tcgaDesign)-1)
y0 <- u[4] + 0.05*(u[4]-u[3]) - rank(-y)/length(y) * (u[4]-u[3])/1.2
d <- density(y)
lines(d$x, d$y/5+u[4]-(u[4]-u[3])/8, col="grey")
lines(d$x, -d$y/5+u[4]-(u[4]-u[3])/8, col="grey")
points(x=y, y=x0+violinJitter(y, magnitude=0.25)$y,pch=19, col=col1[groups[-1]])
text(x=tcgaGlm$coefficients[w,1], y= u[4], "Model coefficients (logFC)", cex=0.8)
v <- tcgaGlm$p.value[w,-1] < 0.01
rotatedLabel(y[v], x0[v]+0.1, labels=colnames(tcgaDesign)[-1][v], font=ifelse(grepl("[[:lower:]]", colnames(tcgaDesign)[-1]),1,3)[v], cex=.66, pos=1)
axis(at=-1:1 + tcgaGlm$coefficients[w,1], labels=-1:1, side=3, cex.axis=.8, line=-1, mgp = c(1.5,.05,0), tcl=-.15)
text(u[2],u[3] + (u[4]-u[3])/10, substitute(paste(R^2==r),list(r=round(Rgenetics[w],2))), pos=2)
}
```

#### Barplot

```
par(bty="n", mgp = c(2.5,.33,0), mar=c(3.5,3.3,2,0)+.1, las=2, tcl=-.25)
b <- barplot(significantGenes, las=2, ylab = "Differentially expressed genes", col=brewer.pal(8,"RdYlBu"), legend.text=FALSE , border=0, xaxt="n")#, col = set1[simple.annot[names(n)]], border=NA)
rotatedLabel(x0=b, y0=rep(10, ncol(significantGenes)), labels=colnames(significantGenes), cex=.7, srt=45, font=ifelse(grepl("[[:lower:]]", colnames(tcgaDesign))[-1], 1,3), col=col1[groups[-1]])
#text(b+0.2, colSums(n)+50, colSums(n), pos=3, cex=.7, srt=90)
x0 <-par("usr")[1] + 0.05 * (par("usr")[2]-par("usr")[1])
dy <- 0.1*(par("usr")[4] - par("usr")[3])
y0 <- par("usr")[4] - 1.5*dy
image(x=x0+c(0,0.8), y=y0+seq(-dy,dy,l=9), z=matrix(1:8, ncol=8), col=brewer.pal(8,"RdYlBu"), add=TRUE)
text(x=x0+1.5, y=y0+seq(-dy/2,dy/2,l=3), format(seq(-1,1,l=3),2), cex=0.66)
lines(x=rep(x0+.8,2), y=y0+c(-dy*.75,dy*.75))
segments(x0+.8,y0+seq(-dy*.75,dy*.75,l=7),x0+.9,y0+seq(-dy*.75,dy*.75,l=7))
text(x0+.8, y0+dy*1.1, "log2 FC", cex=.66)
rotatedLabel(b-0.1, colSums(significantGenes), colSums(significantGenes), pos=3, cex=, srt=45)
```

#### Associated mutations per transcript:

```
par(bty="n", mgp = c(2.5,.33,0), mar=c(3,3.3,3,0)+.1, las=1, tcl=-.25)
t <- table(rowSums(abs(testResults[,1:16])))
b <- barplot(t[-1],ylab="Differentially expressed genes", col=rev(brewer.pal(7, "Spectral")[-(4:5)]), border=NA)
rotatedLabel(b-0.1, t[-1], t[-1], pos=3, cex=1, srt=45)
title(xlab="Associated drivers", line=2)
```

#### Chromosomal distribution

```
chr = factor(sapply(AnnotationDbi::mget(rownames(tcgaExpr), org.Hs.egCHR, ifnotfound=NA), `[`,1), levels=c(1:22, "X","Y","MT"))
chromTable <- apply(testResults,2, function(x) table(chr[x!=0]))
```

```
par(bty="n", mgp = c(0.5,0.5,0), las=1, tcl=-.25, font.main=3, mfrow=c(6,6), xpd=NA, mar=c(0,0,1.5,0))
for(j in 1:ncol(testResults)){
    n <- sum(testResults[,j]!=0)
    pie(chromTable[,j], col=colorRampPalette(brewer.pal(11,'Spectral'))(24), border="white",  radius=0.8, init.angle=90, labels=ifelse(chromTable[,j]/sum(chromTable[,j]) > 0.02, paste("",rownames(chromTable), "(" ,chromTable[,j], ")",sep=""),""))
    title(main = colnames(chromTable)[j], font.main = ifelse(grepl("[[:lower:]]", colnames(chromTable)[j]), 1,3), cex.main=1.33)
    symbols(0,0,circles=.3, inches=FALSE, col="white", bg="white", lty=0, add=TRUE)
    #symbols(0,0,circles=.8*(1-sqrt(n/max(colSums(testResults!=0)))), col="white", add=TRUE, lty=0, bg="white", inches=FALSE)
    #cat(n,"\n")
}
t <- table(chr)
pie(t, col=colorRampPalette(brewer.pal(11,'Spectral'))(24), border="white",  radius=0.8, cex.main=1.33, labels=ifelse(t/sum(t) > 0.02, names(t),""), init.angle=90)
symbols(0,0,circles=.3, inches=FALSE, col="white", bg="white", lty=0, add=TRUE)
title(main = "# Genes", font.main = ifelse(grepl("[[:lower:]]", colnames(chromTable)[j]), 1,3))
```

## 2. Survival analyses

### 2.1 Prepare data

```
library(CoxHD)
tcgaData <- data.frame(tcgaMutation, tcgaClinical[14:24])
tcgaData <- tcgaData[colSums(tcgaData, na.rm=TRUE)>=5]
tcgaData <- ImputeXMissing(data.frame(tcgaData, Gender=tcgaClinical$Gender, scale(tcgaClinical[,c(7:12)])))
rownames(tcgaData) <- rownames(tcgaMutation)
tcgaData <- tcgaData[rowSums(is.na(tcgaData))==0 & rownames(tcgaData) %in% colnames(tcgaExpr),]
dataFrame <- data.frame(tcgaData, scale(tcgaPca$x[rownames(tcgaData),1:20]))
survivalGroups <- rep("Blood", ncol(dataFrame))
survivalGroups[colnames(tcgaData)%in%colnames(tcgaMutation)] <- "Genetics"
survivalGroups[grep("^PC", colnames(dataFrame))] <- "Expression"
survivalGroups[grep("^[a-z]", colnames(dataFrame))] <- "CNA"
survivalGroups[grep("(t_)|_t", colnames(dataFrame))] <- "Translocations"
survivalGroups[grep("(Gender)|(AOD)", colnames(dataFrame))] <- "Demographics"
survivalGroups <- factor(survivalGroups, levels=c("Genetics","CNA","Translocations","Expression","Demographics","Blood"))
survivalCol=set1[c(3,2,5,4,7,1)]#  c(brewer.pal(8, "Dark2")[1:3], brewer.pal(8, "Set1")[2:1])
names(survivalCol) <- levels(survivalGroups)

library(survival)
tcgaSurvival <- Surv(tcgaClinical$OS + .5, tcgaClinical$Status)[match(rownames(tcgaData),tcgaClinical$TCGA_ID)]
```

### 2.2 Fit model and compute variance components of the predicted log-hazard

```
coxRFX <- CoxRFX(dataFrame, tcgaSurvival, which.mu = NULL) ## Further developed version of ecoxph
VarianceComponents(coxRFX, groups=survivalGroups)
```

```
##       Genetics            CNA Translocations     Expression   Demographics          Blood       residual 
##      5.607e-03      1.105e-03      7.751e-05      4.030e-01      2.650e-01      7.343e-02      2.547e-01
```

```
PlotVarianceComponents(coxRFX, col=survivalCol, groups=survivalGroups)
points(0,0,pch=16, col="white", cex=15)
title(main="Variance components AML")
```

#### Stepwise model selection

```
c <- coxph(tcgaSurvival ~ 1, data=dataFrame)
scopeStep <- as.formula(paste("tcgaSurvival ~", paste(colnames(dataFrame), collapse="+")))
coxBIC <- step(c, scope=scopeStep, k = log(sum(!is.na(tcgaSurvival))), trace=0)
```

```
## Warning: is.na() applied to non-(list or vector) of type 'NULL'
## Warning: is.na() applied to non-(list or vector) of type 'NULL'
## Warning: is.na() applied to non-(list or vector) of type 'NULL'
## Warning: is.na() applied to non-(list or vector) of type 'NULL'
```

```
summary(coxBIC)
```

```
## Call:
## coxph(formula = tcgaSurvival ~ AOD + PC4 + PC5 + Wbc + TP53 + 
##     PC17, data = dataFrame)
## 
##   n= 161, number of events= 103 
##    (12 observations deleted due to missingness)
## 
##        coef exp(coef) se(coef)     z Pr(>|z|)    
## AOD   0.540     1.716    0.127  4.24  2.3e-05 ***
## PC4  -0.278     0.757    0.113 -2.47  0.01354 *  
## PC5   0.314     1.369    0.105  2.99  0.00279 ** 
## Wbc   0.342     1.408    0.116  2.95  0.00315 ** 
## TP53  1.186     3.274    0.340  3.49  0.00049 ***
## PC17 -0.238     0.788    0.104 -2.28  0.02235 *  
## ---
## Signif. codes:  0 '***' 0.001 '**' 0.01 '*' 0.05 '.' 0.1 ' ' 1
## 
##      exp(coef) exp(-coef) lower .95 upper .95
## AOD      1.716      0.583     1.337     2.203
## PC4      0.757      1.321     0.607     0.944
## PC5      1.369      0.730     1.114     1.683
## Wbc      1.408      0.710     1.122     1.766
## TP53     3.274      0.305     1.680     6.377
## PC17     0.788      1.269     0.642     0.967
## 
## Concordance= 0.728  (se = 0.032 )
## Rsquare= 0.344   (max possible= 0.996 )
## Likelihood ratio test= 68  on 6 df,   p=1.07e-12
## Wald test            = 64.4  on 6 df,   p=5.84e-12
## Score (logrank) test = 72.8  on 6 df,   p=1.09e-13
```

#### Random forests

```
library(randomForestSRC)
m <- match(rownames(tcgaPca$x),tcgaClinical$TCGA_ID)
rsf <- rfsrc(Surv(time, status) ~ ., data=data.frame(time=tcgaClinical$OS[m], status = tcgaClinical$Status[m], dataFrame ), ntree=100)
```

Variable importance

```
par(bty="n", mgp = c(2,.33,0), mar=c(4,3,1,0.5)+.1, las=2, tcl=-.25, las=3, xpd=NA)
boxplot(rsf$importance ~ survivalGroups, border= survivalCol, staplewex=0, pch=16, cex=0.75, ylab="Variable importance", lty=1, xaxt="n")
rotatedLabel(x0=1:nlevels(survivalGroups), y0=rep(-0.002,nlevels(survivalGroups)), labels=levels(survivalGroups), srt=45)
```

The plot confirms the result that expression, blood counts, and clinical variables are most influental.

#### Subset

```
set.seed(42)
cvIdx <- sample(1:5, nrow(dataFrame), replace=TRUE)
```

```
subsets <- list(Genetics="Genetics", Cytogenetics=c("Translocations","CNA"), Blood="Blood", Demographics="Demographics", Expression="Expression", `Gen+Cyt` = c("Genetics","Translocations","CNA"), `Gen+Cyt+Blo+Exp`=c("Genetics","Translocations","CNA","Blood","Expression"), All=unique(survivalGroups))
colSubsets <- set1
names(colSubsets) <- c("Blood","Cytogenetics", "Genetics","Expression","Gen+Cyt","Gen+Cyt+Blo+Exp","Demographics","All","Std. Risk")
concordance <- sapply(1:5, function(i){
            v <- cvIdx == i
            c(sapply(subsets, function(s,v){
                        w <- survivalGroups %in% s
                        fit <- CoxRFX(dataFrame[!v, w]+0, tcgaSurvival[!v], which.mu=NULL)
                        p <- as.matrix(dataFrame[v,w]) %*% coef(fit)
                        survConcordance(tcgaSurvival[v]~p)$concordance
                    }, v=v), 
            Std.Risk =  survConcordance(tcgaSurvival[v]~ c(3,1,2)[tcgaClinical$C_Risk[match(rownames(tcgaData),tcgaClinical$TCGA_ID)][v]])$concordance
            )
        })
rownames(concordance) <- c(names(subsets), "Std. Risk")
par(mar=c(5,4,1,1), mgp=c(3,0.5,0))
m <- rowMeans(concordance, na.rm=TRUE)
e <- apply(concordance,1,var)/ncol(concordance)
o <- order(m)
barplot(m[o], col=colSubsets[names(m[o])], names.arg=rep("", nrow(concordance)), ylim=c(0.5,0.75), xpd=FALSE, ylab="Concordance (5x CV)") -> b
points(jitter(rep(b,5)), concordance[o,], col="grey", pch=16, cex=.5)
rotatedLabel(b,rep(0.49, nrow(concordance)), rownames(concordance)[o])
segments(b, m[o]+sqrt(e)[o], b , m[o]-sqrt(e)[o])
```

#### PC predictions

The predicted PCs have more predictive power than pure genomics

```
par(mar=c(10,4,1,1))
pcPrediction <- tcgaDesign[,2:31] %*% t(tcgaGlm$coefficients[,2:31]) %*% tcgaPca$rotation ## Genomics only, no offset
set.seed(42)
concordancePrediction <- sapply(1:100, function(i){
            v <- sample(1:nrow(dataFrame) %% 5 + 1) == 1 ## 80:20 split for cross validation
            fit <- CoxRFX(pcPrediction[!v, 1:20], tcgaSurvival[!v], which.mu=NULL)
            p <- as.matrix(pcPrediction[v, 1:20]) %*% coef(fit)
            pcp <- survConcordance(tcgaSurvival[v]~p)$concordance[1]
            fit <- CoxRFX(tcgaPca$x[!v, 1:20], tcgaSurvival[!v], which.mu=NULL)
            p <- as.matrix(tcgaPca$x[v, 1:20]) %*% coef(fit)
            pc <- survConcordance(tcgaSurvival[v]~p)$concordance[1]
            fit <- CoxRFX(dataFrame[!v,survivalGroups %in% c("Genetics","Cytogenetics")], tcgaSurvival[!v], which.mu=NULL)
            p <- as.matrix(dataFrame[v, survivalGroups %in% c("Genetics","Cytogenetics")]) %*% coef(fit)
            gen <- survConcordance(tcgaSurvival[v]~p)$concordance[1]
            c(Expression=pc, Genomics=gen, `Expression (predicted)`=pcp)
        })
c <- set1[c(4,2,4)]
boxplot(t(concordancePrediction), notch=TRUE, ylab="Concordance", names=NA, lty=1, staplewex=0, pch=16, xaxt="n", at=1:3, border=c, col=c(NA,NA, brewer.pal(4,"Paired")[1]))
u <- par("usr")
rotatedLabel(1:3, rep(u[3] ,3), c("Expression","Genomics","Predicted expression"))
```

## Session

```
sessionInfo()
```

```
## R version 3.0.1 Patched (2013-07-31 r63471)
## Platform: x86_64-apple-darwin12.3.0 (64-bit)
## 
## locale:
## [1] en_GB.UTF-8/en_GB.UTF-8/en_GB.UTF-8/C/en_GB.UTF-8/en_GB.UTF-8
## 
## attached base packages:
##  [1] grid      splines   parallel  stats     graphics  grDevices utils     datasets  methods   base     
## 
## other attached packages:
##  [1] VennDiagram_1.6.4       rtracklayer_1.21.9      rj_1.1.3-1              randomForestSRC_1.3    
##  [5] markdown_0.7.4          GO.db_2.9.0             GenomicFeatures_1.13.25 GenomicRanges_1.13.36  
##  [9] XVector_0.1.0           IRanges_1.19.24         biomaRt_2.17.2          affy_1.39.2            
## [13] Hmisc_3.12-2            Formula_1.1-1           xtable_1.7-1            mg14_0.0.1             
## [17] CoxHD_0.0.27            MASS_7.3-26             survival_2.37-7         glmnet_1.9-3           
## [21] Matrix_1.0-12           lattice_0.20-14         cgdsr_1.1.30            RColorBrewer_1.0-5     
## [25] hgu133plus2.db_2.9.0    org.Hs.eg.db_2.10.1     RSQLite_0.11.4          DBI_0.2-7              
## [29] AnnotationDbi_1.24.0    Biobase_2.21.6          BiocGenerics_0.7.3      limma_3.17.21          
## [33] knitr_1.6              
## 
## loaded via a namespace (and not attached):
##  [1] affyio_1.29.0         BiocInstaller_1.12.1  Biostrings_2.29.15    bitops_1.0-5          BSgenome_1.29.1      
##  [6] cluster_1.14.3        codetools_0.2-8       digest_0.6.3          evaluate_0.5.5        formatR_0.10         
## [11] preprocessCore_1.23.0 R.methodsS3_1.6.1     R.oo_1.18.0           RCurl_1.95-4.1        rpart_4.1-1          
## [16] Rsamtools_1.13.29     stats4_3.0.1          stringr_0.6.2         tools_3.0.1           XML_3.98-1.1         
## [21] zlibbioc_1.7.0
```
